# Dopamine, Immunity, and Disease

**DOI:** 10.1124/pharmrev.122.000618

**Published:** 2023-01

**Authors:** Breana Channer, Stephanie M. Matt, Emily A. Nickoloff-Bybel, Vasiliki Pappa, Yash Agarwal, Jason Wickman, Peter J. Gaskill

**Affiliations:** Department of Pharmacology and Physiology, Drexel University College of Medicine, Philadelphia, Pennsylvania (B.C., S.M.M., E.A.N-B., Y.A., J.W., P.J.G.); and The Children’s Hospital of Philadelphia Research Institute, Philadelphia, Pennsylvania (V.P.)

## Abstract

The neurotransmitter dopamine is a key factor in central nervous system (CNS) function, regulating many processes including reward, movement, and cognition. Dopamine also regulates critical functions in peripheral organs, such as blood pressure, renal activity, and intestinal motility. Beyond these functions, a growing body of evidence indicates that dopamine is an important immunoregulatory factor. Most types of immune cells express dopamine receptors and other dopaminergic proteins, and many immune cells take up, produce, store, and/or release dopamine, suggesting that dopaminergic immunomodulation is important for immune function. Targeting these pathways could be a promising avenue for the treatment of inflammation and disease, but despite increasing research in this area, data on the specific effects of dopamine on many immune cells and disease processes remain inconsistent and poorly understood. Therefore, this review integrates the current knowledge of the role of dopamine in immune cell function and inflammatory signaling across systems. We also discuss the current understanding of dopaminergic regulation of immune signaling in the CNS and peripheral tissues, highlighting the role of dopaminergic immunomodulation in diseases such as Parkinson’s disease, several neuropsychiatric conditions, neurologic human immunodeficiency virus, inflammatory bowel disease, rheumatoid arthritis, and others. Careful consideration is given to the influence of experimental design on results, and we note a number of areas in need of further research. Overall, this review integrates our knowledge of dopaminergic immunology at the cellular, tissue, and disease level and prompts the development of therapeutics and strategies targeted toward ameliorating disease through dopaminergic regulation of immunity.

**Significance Statement:**

Canonically, dopamine is recognized as a neurotransmitter involved in the regulation of movement, cognition, and reward. However, dopamine also acts as an immune modulator in the central nervous system and periphery. This review comprehensively assesses the current knowledge of dopaminergic immunomodulation and the role of dopamine in disease pathogenesis at the cellular and tissue level. This will provide broad access to this information across fields, identify areas in need of further investigation, and drive the development of dopaminergic therapeutic strategies.

## I. Introduction

### A. Overview

Dopamine, or 3-hydroxytyramine, is an endogenous catecholamine that is important to both neuronal and nonneuronal processes. Dopamine was first synthesized in 1910, and initial studies examined its biologic effect as a weak sympathomimetic,although the mechanism of action was not clear (Barger and Dale, 1910). Neurotransmitters, initially acetylcholine, were defined as the chemical agents mediating communication in nerve pulses by Drs. Otto Loewi and Henry Dale in the first decades of the 20th century (Valenstein, 2002). The catecholamines norepinephrine and dopamine were determined to be neurotransmitters in the middle of the 20th century, norepinephrine by Drs. Ulf von Euler, Bernard Katz, and Julius Axelrod and dopamine by Drs. Arvid Carlsson and Paul Greengard (Benes, 2001; Snyder, 2006; Iversen and Iversen, 2007).

The biosynthetic pathways associated with the synthesis of dopamine were first hypothesized by Hermann Blaschko (Blaschko, 1957), and with the discovery of dopamine in the peripheral tissues of mammals (Goodall, 1951; Von Euler and Hellner, 1951), it was shown to be a precursor of the catecholamines norepinephrine and epinephrine. Carlsson and colleagues found that dopamine has a unique distribution pattern throughout the brain, plasma, and other tissues within the human body (Carlsson et al., 1957, 1958; Weil-Malherbe and Bone, 1957; Bertler and Rosengren, 1959; Imai et al., 1970). Early reports identified the largest amounts of dopamine in the striatum, particularly the caudate nucleus. Studies using both dopamine and dopaminergic drugs found that dopamine could inhibit neuronal discharge and was critical for extrapyramidal function, particularly motor function (Hornykiewicz, 1966). In 1960, Ehringer and Hornykiewicz demonstrated dopamine deficits in patients with parkinsonism, indicating that extrapyramidal activity made dopamine a central factor in Parkinson’s disease (PD) (Ehringer and Hornykiewicz, 1960, 1998). Today, central nervous system (CNS) dopamine has a well-established role in motor control, cognition, learning, and reward. In the periphery, dopamine regulates gastrointestinal (GI) motility, sodium levels, blood pressure maintenance, hormone release, and many other functions (Baines and Drangova, 1997; Fitzgerald and Dinan, 2008; Harris and Zhang, 2012; Bove et al., 2019). Reductions in dopamine underlie diseases such as PD and attention-deficit/hyperactivity disorder (ADHD), while elevated dopamine states have been implicated in schizophrenia (Birtwistle and Baldwin, 1998).

In addition, research over the past few decades has shown that dopamine can have a substantial impact on immune cell function in both the CNS and periphery. Dopaminergic immunomodulation affects both innate and adaptive immunity and has become increasingly important as a possible target for drug discovery and disease management. While there have been numerous reviews that detail individual parts of these topics (Levite et al., 2017; Pinoli et al., 2017; Matt and Gaskill, 2020; Thomas Broome et al., 2020; Vidal and Pacheco, 2020), this field is progressing rapidly, and there is still considerable controversy regarding the understanding of dopamine as an immunoregulatory factor. This review aims to explore, organize, and consolidate what is known about the immunoregulatory actions of dopamine, from the regulation of specific immune functions to its role in disease pathogenesis, providing a firm foundation on which to move these types of studies forward. Of note, while all substance use disorders (SUDs) dysregulate the dopaminergic system, the direct effects of addictive substances on the immune system, is an overlapping but distinct topic from the role of dopamine itself. There is a growing interest in the bidirectional interaction(s) between the immune system and SUDs, and this topic has been extensively covered in other reviews (Cui et al., 2014; Lacagnina et al., 2017; Namba et al., 2021). Therefore, it is only discussed briefly in reference to other topics.

### B. Dopamine Signaling

**Fig. 1:**
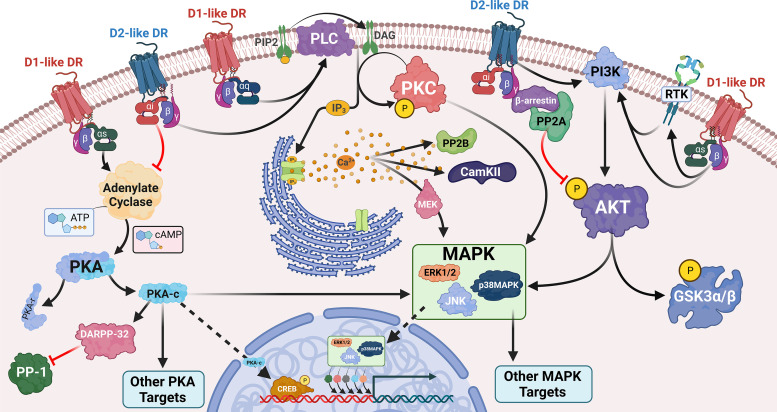
Dopamine signaling through cognate receptors. Dopamine signaling is mediated through its GPCRs. D1-like receptors (D1 and D5, red) classically couple to G_as_ to mediate activation of adenylate cyclase, leading to cAMP production, PKA activation, and downstream activation of PKA targets. The D2-like receptors (D2, D3, and D4, blue) couple to the G_ai_ pathway to inhibit adenylate cyclase production and oppose D1-like signaling. The D1-like receptors can also lead to activation of PLC*β*, thus enhancing calcium flux and PKC activation. The D2-like receptors can also activate this pathway via G_bg_. D2-like stimulation can additionally inhibit AKT phosphorylation through the formation of a b-arrestin/PP2A signaling complex. Both D1-like and D2-like stimulation leads to AKT phosphorylation through its activity on the phosphatidylinositol 3-kinase (PI3K)/Akt signaling axis, but the mechanisms behind this are not clear. Downstream, both receptors can activate members of the MAPK family. This occurs through various mechanisms including, but not limited to, cAMP activation, PKC and calcium signaling, and activation of the PI3K/Akt signaling cascade. Created with BioRender.com.

Dopamine primarily mediates its effects on different cell types by signaling through dopamine receptors, which are G-protein coupled receptors (GPCRs). An overview of these signaling cascades, delineating the pathways described in this section, is shown in Fig. 1. Paul Greengard and colleagues defined two distinct signaling pathways that activate or inhibit the ubiquitous second messenger 3′-5′ cAMP and are mediated by different types of dopamine receptors (Hemmings et al., 1984). Based on these pathways, the five dopamine receptor subtypes are often grouped into stimulatory D1-like dopamine receptors (D1 and D5) and inhibitory D2-like dopamine receptors (D2, D3, and D4) (Missale et al., 1998; Beaulieu and Gainetdinov, 2011). Dopamine receptors can also be grouped into low affinity (D1 and D2) and high affinity (D3, D4, and D5) receptors depending on their affinity for dopamine. The regulation of cAMP production is mediated by the release of heterotrimeric G proteins that are coupled to each dopamine receptor; D1-like receptors stimulate the production of cAMP, and D2-like receptors inhibit it.

More specifically, dopamine binding to a dopamine receptor triggers a conformational shift that allows a guanine exchange factor to exchange a guanosine diphosphate for the guanosine triphosphate bound to the *α* subunit of the heterotrimeric G-protein. This releases the coupled G-protein and facilitates its dissociation into *α* and *βγ* subunits. The *α* subunit then acts on adenylate cyclase, an enzyme that catalyzes the conversion of ATP into cAMP. The specific effect on adenylate cyclase depends on the released G-protein. D1-like receptor activation releases G_s_ alpha subunits of the G_s_ heterotrimeric G protein (G*_α_*_s_), which activate adenylate cyclase, increasing intracellular concentrations of cAMP (Kebabian and Greengard, 1971; Kebabian, 1978). Activation of both D1-like receptors increases production of cAMP in transfected cells, but some knockout studies suggest that D1 is more strongly linked to this response relative to D5 (Undie and Friedman, 1994; Undieh, 2010). In contrast to D1-like receptors, D2-like receptors are coupled to the inhibitory G_i_ alpha subunit of the G_i_ heterotrimeric G protein (G*_α_*_i_). D2-like receptor activation inhibits the activity of adenylate cyclase, reducing cAMP production and decreasing the downstream activity induced by D1-like receptors (Boyd and Mailman, 2012).

cAMP primarily activates protein kinase A (PKA), but studies have shown that cAMP can also activate exchange factor directly activated by cAMP (EPAC) and protein kinase C (PKC). PKA activation leads to the transcription of cAMP response element binding protein (CREB), which triggers the transcription of a variety of genes (Beaulieu et al., 2015; Wang, Xu et al., 201). In neurons, dopamine and cAMP-regulated phosphoprotein is also a major target of PKA and is a critical mediator of dopamine signaling (Svenningsson et al., 2004), although the activity of dopamine and cAMP-regulated phosphoprotein in nonneuronal cells is poorly understood. PKA also modulates mitogen-activated protein kinase (MAPK) family activation, although this appears to be cell type–specific and may involve other downstream signaling effectors (Zhen et al., 1998; Skalhegg and Tasken, 2000; Han et al., 2007).

Although cAMP signaling is most associated with dopamine receptor activation, a growing number of studies have shown important effects of alternative GPCR-dependent and -independent pathways. Activation of both D1-like and D2-like receptors can activate protein lipase C-*β* (PLC*β*) to induce inositol triphosphate (IP_3_)-mediated intracellular calcium flux. Unlike the effect on cAMP signaling, both dopamine receptor subtypes mediate similar effects on IP_3_-mediated calcium flux despite different signaling cascades (Beaulieu and Gainetdinov, 2011; Beaulieu et al., 2015). In this pathway, D1-like receptors couple to G*_α_*_q_ and stimulate PLC*β*, leading to diacylglycerol and IP_3_ production, and the subsequent activation of Ca^2+^ and PKC (Jin et al., 1998; Wang et al., 1995; Jin et al., 2001; Zhang et al., 2009; Undieh, 2010; Medvedev et al., 2013). Knockout studies showed that dopamine activated PLC*β* in Drdr1 knockout animals but not Drd5 knockout animals (Sahu et al., 2009), and earlier studies showed that only one D1-like isoform could modulate PLC*β* activity (Friedman et al., 1997). This finding suggests that D5 may be the specific D1-like receptor that mediates G*_α_*_q_ activity and that the two D1-like receptors may mediate different effects, although this may be tissue-specific (Undieh, 2010). D2-like receptor activation can also stimulate PLC*β* (Di Marzo et al., 1993; Hernandez-Lopez et al., 2000), acting through G*_βγ_* subunits rather than G*_α_*_q_ subunits (Choi et al., 1999; Hernandez-Lopez et al., 2000; Beaulieu et al., 2015). Dopamine-mediated PLC*β* activation has been observed in a number of cell types, including striatal neurons (Friedman et al., 1997; Sahu et al., 2009), renal proximal tubule cells (Felder et al., 1989; Vyas et al., 1992), and immune cells (Nickoloff-Bybel et al., 2019), suggesting that this effect may be central to many of the actions of dopamine.

Dopamine signaling can also be mediated through G-protein-independent pathways, such as the activation of *β*-arrestins. Classically, these proteins regulate receptor internalization but have also been shown to trigger specific signaling activities (Lefkowitz and Shenoy, 2005; DeWire et al., 2007; Defea, 2008; Jean-Charles et al., 2017). In addition, at higher concentrations, dopamine can activate *α*- and *β*-adrenergic receptors (Lei, 2014), triggering a distinct but often overlapping set of downstream pathways. Dopamine-induced *β*-arrestin signaling is primarily mediated by D2-like receptors (Beaulieu et al., 2005, 2015; Peterson et al., 2015) which recruit protein phosphatase 2A (PP2A) and cause the subsequent inhibition of protein kinase B (Akt) (Beaulieu et al., 2004; Radl et al., 2013; Zhang, Jiang et al., 2016; Han et al., 2017; Wu et al., 2020). These and other data indicate that the view of D1-like receptors as stimulatory (G*_α_*_s_) and D2-like receptors as inhibitory (G*_α_*_i_) is only partially accurate, as D1- and D2-like receptors can stimulate both opposing and overlapping signaling mechanisms through non-cAMP pathways.

It is challenging to define all the dopamine-driven signaling cascades that are downstream of cAMP, Ca^2+^, and PLC*β*, as dopamine receptors can activate a wide range of additional proteins and effectors, including the MAPKs extracellular signal-regulated kinase 1/2 (ERK1/2), p38 MAPK, and c-Jun N-terminal kinase (JNK)/stress-activated protein kinase, L-type calcium channels, Akt, and AMP-activated protein kinase (Welsh et al., 1998; Zhen et al., 1998; Cussac et al., 1999; Yan et al., 1999; Zhen et al., 2001; Brami-Cherrier et al., 2002; Nair et al., 2003; Nair and Sealfon, 2003; Wang et al., 2005; Ming et al., 2006; Han et al., 2007; Liu et al., 2009; Mannoury la Cour et al., 2011; Chen, Ruan et al., 2012; Perreault et al., 2013; Yoon and Baik, 2013; Franz et al., 2015; Bone et al., 2017; Fan et al., 2018). The precise signaling pathways involved in the activation of these effectors are complicated, often overlapping, and vary by cell type. For example, both D1- and D2-like receptors have been shown to activate all three members of the MAPK family: p38 MAPK, JNK, and ERK1/2; however, the specific MAPKs involved and the distinct roles of dopamine receptors vary from system to system. In mouse embryonic stem cells, dopamine, the D1-like agonist SKF-38393, and the D2-like agonist quinpirole activated all three members of the MAPK family (Lee et al., 2006). However, in neuroblastoma cells, D1-like agonists only activated p38 MAPK and JNK, and no MAPKs were activated by quinpirole (Zhen et al., 1998). Moreover, in CD4^+^ T-cells, D5 stimulation activates ERK1/2, while D3 stimulation was linked to inhibition of this protein (Franz et al., 2015).

Similarly, dopamine may have varying impacts on Akt activation in different systems. Dopamine both positively and negatively regulates Akt (Zhen et al., 2001; Brami-Cherrier et al., 2002; Nair et al., 2003; Nair and Sealfon, 2003; Beaulieu and Gainetdinov, 2011; Mannoury la Cour et al., 2011; Chen, Ruan et al., 2012; Perreault et al., 2013; Radl et al., 2013; Mirones et al., 2014; Beaulieu et al., 2015; Tolstanova et al., 2015; Zhang, Jiang et al., 2016; Gao et al., 2017; Han et al., 2017; Wu et al., 2020; Yan et al., 2020), although D2-like receptors are most commonly associated with Akt inhibition (Beaulieu et al., 2007, 2015; Beaulieu and Gainetdinov, 2011; Radl et al., 2013; Tolstanova et al., 2015; Zhang, Jiang et al., 2016; Han et al., 2017). Akt inhibition is likely mediated by *β*-arrestin-induced recruitment of PP2A, which inhibits the phosphorylation and activation of Akt (Beaulieu et al., 2005, 2007, 2015; Beaulieu and Gainetdinov, 2011).

A substantial amount of dopaminergic immunomodulation can be mediated by MAPK and Akt activity, as these proteins modulate many immune activities. MAPKs can regulate functions such as cytokine/chemokine production and phagocytosis (Karin, 1995; Cuenda and Rousseau, 2007; Kaminska et al., 2009; Cargnello and Roux, 2011; Kyriakis and Avruch, 2012), while Akt inhibition may mediate some of the anti-inflammatory effects of dopamine receptors (Zhang, Jiang et al., 2016; Han et al., 2017; Wu et al., 2020). Taken together, these data highlight the complexity of dopamine signaling and indicate that cell type–specific variations in dopamine receptor responses to the same ligands likely account for the diverse effects of dopamine on the functions of different immune cells.

### C. Dopamine Production and Metabolism

**Fig. 2:**
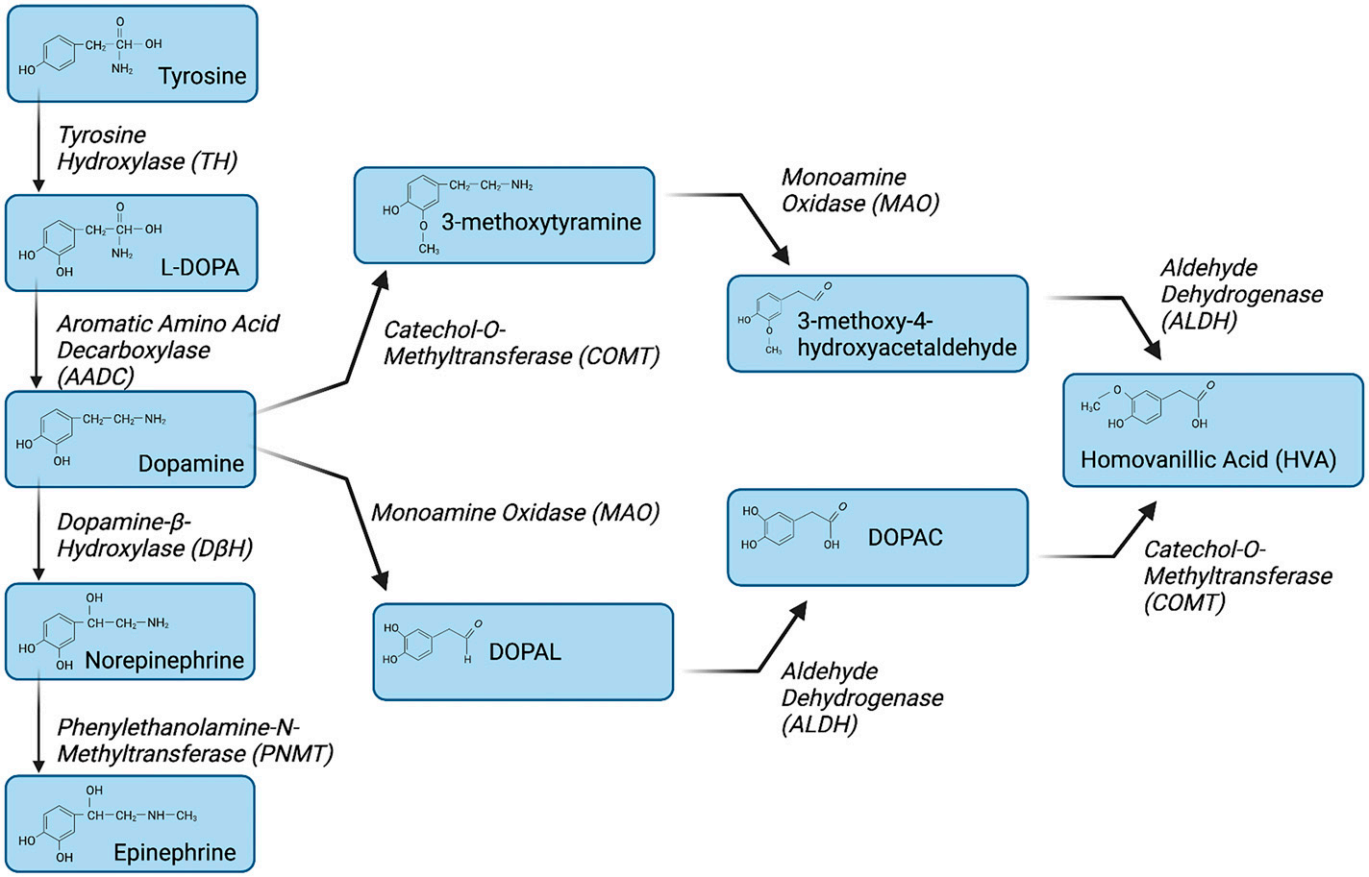
Metabolic pathway of dopamine biosynthesis and degradation. Dopamine synthesis is initiated with the hydroxylation of tyrosine by the enzyme TH to generate L-DOPA. L-DOPA is converted to dopamine by AADC. Dopamine beta hydroxylase (DBH) hydroxylates dopamine to form norepinephrine, which is converted to epinephrine by phenylethanolamine-N-methyltransferase (PNMT). Dopamine is primarily metabolized by two enzymatic pathways, COMT and MAO. COMT converts dopamine to 3-methoxytyramine, which is subsequently converted to 3-methoxy-4-hydroxyacetaldehyde by MAO. In contrast, MAO converts dopamine to 3,4-dihydroxyphenylacetaldehyde, which is then converted by aldehyde dehydrogenase (ALDH) to DOPAC. In the final steps of dopamine degradation, ALDH and COMT convert 3-methoxy-4-hydroxyacetaldehyde and DOPAC to HVA, respectively. Created with BioRender.com

The synthesis of dopamine has been most widely studied in dopaminergic neurons in the CNS, in which dopamine is synthesized from tyrosine. An overview of the dopamine synthesis and degradation pathways described in this section is found in Fig. 2. Tyrosine is produced from phenylalanine in the liver through the action of phenylalanine hydroxylase and is transported into dopaminergic neurons after crossing the blood–brain barrier through the large neutral amino acid transporter. In dopaminergic neurons, tyrosine hydroxylase (TH) catalyzes the addition of a hydroxyl group to the meta position of tyrosine to produce levodopa (L-DOPA) (Meiser et al., 2013). This process is considered the rate limiting step in this pathway and is susceptible to end-product inhibition by high levels of catecholamines. However, it is much less affected by changes in tyrosine levels, as TH is typically saturated with substrate. Once produced, L-DOPA is rapidly converted to dopamine by aromatic L-amino acid decarboxylase (AADC) (Meiser et al., 2013). In dopaminergic neurons, newly synthesized dopamine is transported from the cytoplasm into synaptic vesicles through vesicular monoamine transporter 2 (VMAT2). In adrenergic neurons containing dopamine-*β*-hydroxylase, dopamine can be further hydrolyzed to generate norepinephrine (Hoffman et al., 1998). Once produced and packaged into vesicles, dopamine can be released when the vesicles fuse with the cell membrane to release the packaged dopamine.

Extracellular dopamine mediates its effects by binding to dopamine receptors and can be recycled via active transport back into dopaminergic neurons by monoamine transporters. The primary transporter that mediates dopamine uptake is the dopamine transporter (DAT), but in brain regions with low DAT expression, dopamine is taken up by the norepinephrine transporter (Moron et al., 2002). Once dopamine is taken back up, it can be repackaged into VMAT2-expressing vesicles, which are then re-released, recycling the neurotransmitter. Alternatively, in both neurons and other cell types, dopamine can be metabolized to form homovanillic acid (HVA). The two major degradation pathways that generate HVA begin with oxidative deamination by monoamine oxidases (MAOs), or O-methylation by catechol-O-methyltransferase (COMT). These pathways generate a variety of intermediate metabolites, including dihydroxyphenylacetaldehyde, 3,4 dihydroxyphenylacetic acid (DOPAC), and 3-methoxytyramine, which are ultimately metabolized to HVA (Kopin, 1985; Thomas Broome et al., 2020). These regulatory pathways are often the targets of therapeutic drugs prescribed for diseases mediated by dopaminergic dysregulation, such as neuropsychiatric disorders (Laatikainen et al., 2012; Finberg, 2019). Importantly, the processes described here apply mainly to the production and metabolism of dopamine in the CNS as mediated by dopaminergic neurons. As discussed in the following text, many immune cells also express these proteins, and data indicate that these processes occur in several immune cell types. However, the processes regulating dopamine synthesis and metabolism in immune cells, how they play into the function of these cells, and the role of dopamine in disease processes remain poorly understood.

### D. Dopamine Release and Uptake

Classically, dopaminergic neurons release dopamine when an action potential reaches the axon terminal, inducing dopamine-containing vesicles to fuse with the cell membrane and release dopamine via exocytosis (Liu and Kaeser, 2019). Within dopaminergic neurons, two temporally distinct modes of signaling are traditionally discussed: phasic and tonic firing. Phasic firing involves synchronized burst firing, which results in fast and transient dopamine release (seconds), while tonic firing involves asynchronous spontaneous firing that produces slow and widespread dopamine release (minutes to hours) (Hauber, 2010). The amount of dopamine that is released in both the CNS and in the periphery depends on the region or tissue being examined and the stimulus involved, but the precise amounts in humans remain largely unclear. However, studies in rodents suggest that tonic firing releases dopamine in the nanomolar range while phasic firing increases the released dopamine concentrations up to micromolar levels (Matt and Gaskill, 2020).

Extracellular dopamine mediates communication by binding to dopamine receptors on neighboring cells. Classic neurotransmission refers to dopamine receptors on the postsynaptic neuron and point-to-point transfer of dopamine in the synaptic cleft. This type of direct interaction within well-defined physical boundaries, such as axons and their synapses, represents a discrete type of transmission known as wired transmission. This type of communication was long considered to be the primary form of dopaminergic neurotransmission (Agnati et al., 2010). However, more precise mapping of neurotransmitter location has shown that a slower, less directed form of communication known as volume transmission also plays a substantial role in dopaminergic neurotransmission. Volume transmission is characterized by extrasynaptic dopamine receptor activation via dopamine spillover or by nonsynaptic release of dopamine into the extracellular space (Venton et al., 2003; Fuxe et al., 2015; Borroto-Escuela et al., 2018). Recent data suggest that dopamine spillover from the synapse plays a minor role in this process, suggesting that volume transmission is largely due to focused release and the diffusion of dopamine at extrasynaptic sites (Wiencke et al., 2020).

Because dopaminergic neurons have broad arbors, dopamine released via spillover and extrasynaptic release increase dopamine concentrations in a large area of surrounding tissue. This can activate extrasynaptic dopamine receptors on neighboring neurons, as well as other nearby cells, such as immune cells, that may be a considerable distance away from the synapse (Rice and Cragg, 2008; Rice et al., 2011). Because increased concentrations of dopamine lead to the diffusion of dopamine throughout a greater area of CNS tissue, the area and number of cells exposed to dopamine is directly proportional to the amount of dopamine released. However, the rates and distances of diffusion can vary widely across the CNS, depending on the concentration and volume of dopamine release, DAT uptake dynamics, the regional specificity of the dopamine receptors, and the volume of extracellular fluid (Sulzer et al., 2016; Matt and Gaskill, 2020).

The function of DAT is particularly important for the regulation of dopaminergic signaling, as this transporter is a critical regulator of extracellular dopamine concentrations. DAT dysregulation or neuronal death/dysfunction that reduces the amount of functional DAT in a specific area could expose larger brain areas and the immune cells in those regions to increased concentrations of dopamine. Indeed, DAT dysregulation is implicated in several distinct pathologies, including ADHD, PD, and bipolar disorder (Vaughan and Foster, 2013; Bastos et al., 2018; Mackie et al., 2018). Most studies that model DAT regulation of dopamine concentrations focus on neuronal DAT, but DAT is also expressed and active on numerous cell types, including glia (Schomig et al., 1998; Meng et al., 1999; Takeda et al., 2002) and many types of immune cells (McKenna et al., 2002; Meredith et al., 2005; Mackie et al., 2018, 2022). The role of immune cell DAT, although not well understood, will be further discussed in subsequent sections.

### E. Dopamine and Oxidative Stress

The induction of oxidative stress is another important mechanism by which dopamine drives several disparate effects that are primarily associated with the development of pathologic conditions. Oxidative damage associated with dopamine includes dopamine auto-oxidation, *α*-synuclein aggregation, glial cell activation, alterations in calcium signaling, mitochondrial dysfunction, and excess free iron (Juarez Olguin et al., 2016). Dopamine and its metabolites contain 2-hydroxyl residues, which generate highly reactive dopamine and DOPA quinones (Miyazaki and Asanuma, 2008; Meiser et al., 2013). The production of these highly reactive quinones not only can be the result of enzymatic oxidation by metal catalysis (Fe^3+^) or by cyclooxygenases, tyrosinases, or other enzymes but can also occur spontaneously. These reactions can then generate superoxide radical anions by donating electrons to oxygen (Graham et al., 1978; Stokes et al., 1999; Sulzer and Zecca, 2000; Meiser et al., 2013). In the presence of iron, dopamine quinones can also further react to form 6-hydroxydopamine, which is a neurotoxin (Simola et al., 2007). These products [dopamine quinones, 6-hydroxydopamine and reactive oxygen species (ROS) such as O^2-^] can be particularly harmful to cells by reacting nonspecifically, causing irreversible cell damage and apoptosis. The interplay of oxidative stress and neuroinflammation is a major factor in the impact of dopamine on immune function and has been shown to synergistically promote the progression of neurodegenerative diseases such as PD and Alzheimer’s disease (Asanuma et al., 2003; Jiang, Sun et al., 2016).

### F. Dopaminergic Pathways in the CNS

The dopaminergic neurons that make up the nigrostriatal, mesolimbic, and mesocortical pathways are largely present in midbrain regions, specifically the substantia nigra (SbN), ventral tegmental area (VTA), and arcuate nucleus of the hypothalamus (Scarr et al., 2013; Nolan and Gaskill, 2019). Their efferent projections reach the striatum, nucleus accumbens (NAc), and several regions in the cortex, respectively. Classic slow-firing properties are seen in dopaminergic neurons that project to the dorsal striatum and NAc shell, while fast-firing properties are seen in dopaminergic neurons in the medial VTA that project to the amygdala or NAc core (Lammel et al., 2008; Hauber, 2010). Dopamine concentrations in the nigrostriatal, mesolimbic, and mesocortical pathways range from 10^−8^M to 10^−5^M. The microglia and macrophages in these pathways are in relatively close proximity to neurons and could be exposed to these dopamine concentrations during homeostatic function. A more in-depth discussion of the concentrations of dopamine to which CNS immune cells may be exposed can be found in the recent review by Matt and Gaskill (2020).

Within the tuberoinfundibular pathway, which is the fourth major dopaminergic pathway in the CNS, neurons project to the median eminence and are primarily responsible for the regulation of prolactin (Gudelsky, 1981; Scarr et al., 2013). Unlike the majority of dopaminergic neurons, most tuberoinfundibular dopaminergic neurons are categorized as secretory neurons due to their lack of synaptic contacts (Ben-Jonathan and Hnasko, 2001). Dopamine released by tuberoinfundibular dopaminergic neurons can diffuse through the perivascular space and is transported to the pituitary gland by portal blood. Moreover, in this pathway, continuously high exposure to dopamine signals via D2 receptors to suppress the activity of pituitary lactotrophs by inhibiting prolactin through the control of calcium flux (Ben-Jonathan and Hnasko, 2001). In the pituitary, studies have identified macrophages (Fujiwara et al., 2017) and dendritic cells (DCs) (Glennon et al., 2015) that could play a role in communicating immune activation to the hypothalamic–pituitary–adrenal (HPA) axis. Dopamine concentrations in the tuberoinfundibular pathway range from 10^−8^M to 10^−4^M in both the hypothalamus and pituitary, and immune cells could be exposed to significant dopamine fluctuations due to diet (Volkow et al., 2011) or the regulation of prolactin production (Lyons et al., 2012).

### G. Peripheral Dopamine

The role of dopamine in the periphery was first described in 1972 in the renal and cardiovascular systems (Goldberg, 1972). Since then, peripheral dopamine has been shown to influence many critical functions in the periphery, such as blood pressure, GI motility, respiration, and immune activation (Goldstein et al., 1995; Rubí and Maechler, 2010; Grassi and Ram, 2016; Matt and Gaskill, 2020). Peripheral and central nervous systems share some mechanisms and molecular machinery, but studies suggest that peripheral dopaminergic systems act via pathways distinct from those in the CNS. There are substantial genetic and expression level differences in dopaminergic proteins in peripheral cells relative to CNS cells, as well as different dopamine release and uptake mechanisms (Wong et al., 1995; Myohanen et al., 2010; Zeng and Jose, 2011). The concentrations of dopamine in peripheral regions vary and were previously believed to originate from the nervous system via crosstalk or from mesenteric regions. It is now clear that while dopamine is released from these regions, it can also be generated in other cell types and peripheral organs. For example, in the kidney or adrenal medulla, dopamine can act as an autocrine/paracrine regulator of local organ function (Eisenhofer et al., 1997; Matt and Gaskill, 2020).

Changes in plasma dopamine are partially determined by sympathetic nerve activity, as increased peripheral dopamine is associated with increases in sympathetic activity, and patients with a loss of sympathetic nerve function have low plasma dopamine concentrations (Goldstein and Holmes, 2008). Sympathetic nerves release dopamine through vesicles that release both dopamine and norepinephrine via exocytosis (Goldstein and Holmes, 2008). Dopamine is also directly released into the circulation from chromaffin cells in the adrenal medulla, as well as amine precursor uptake and decarboxylation cells, which are found predominantly in the kidney (Wolfovitz et al., 1993). The high levels of plasma dopamine are not well understood, but one possible explanation is an underestimation of the amount of dopamine synthesis and metabolism that occurs in nonneuronal cells in the periphery. This notion is supported by high urinary excretion of DOPAC and HVA, as well as very high concentrations of dopamine conjugates in the periphery (Eisenhofer et al., 1997). Free dopamine levels in the circulation are in the picomolar to femtomolar range and only make up 5% of dopamine in plasma (Kuchel and Kuchel, 1991). Most peripheral dopamine is conjugated as sulfates or glucuronides, which are biologically inactive (Yoneda et al., 1983). There are also substantial amounts of both types of dopamine conjugates in the CNS (Suominen et al., 2013), again exceeding the concentrations of free dopamine in these regions. Data suggest that the source of these dopamine conjugates may be the adrenal gland (Wang et al., 1983; Uutela et al., 2009).

Dopamine sulfate has a half-life of a few hours compared with a few minutes for unmodified dopamine (Eldrup, 2004). The sulfo-conjugation mechanism seems relatively independent of sympathetic nerves, as the loss of sympathetic nerve function does not decrease plasma levels of dopamine sulfate. However, ingestion of a standard meal increases plasma dopamine sulfate concentrations by more than 50-fold, suggesting that sulfo-conjugation may depend on diet and dopamine conjugation in the GI tract (Goldstein et al., 1999). Dopamine sulfate conjugation is hypothesized to take place before dopamine enters the bloodstream, and very little dopamine sulfate is formed from circulating dopamine. This mechanism may localize the effects of bioactive dopamine due to diet or local dopamine produced by peripheral tissues, and/or it may be used to inactivate dopamine when it enters circulation to prevent toxicity and catecholamine buildup (Goldstein et al., 1999). Inactivating circulating dopamine may be important as variations in the ability to sulfo-conjugate dopamine increases the risk of certain diseases (Suominen et al., 2015).

Unlike dopamine inactivation by deamination or O-methylation, sulfo-conjugation is reversible. Dopamine sulfate can be converted back to bioactive dopamine by the enzyme arylsulfatase A (Strobel et al., 1990), which is found in the liver, lung, brain, and adipose tissue (Richard et al., 2001; Borcherding et al., 2011). Small amounts of dopamine sulfate can cross the blood–brain barrier (Suominen et al., 2015), and the levels of the UDP-glucuronosyltransferases and phenol sulfotransferases (needed for dopamine conjugation have been reported in rat and human brain (King et al., 1999; Kauffman, 2004). It has also been shown that dopamine can induce its own sulfation metabolism and that inhibiting the sulfotransferase SULT1A3 significantly increased the susceptibility of cells to dopamine toxicity (Sidharthan et al., 2013). This suggests that sulfation is a mechanism to protect cells from damage and could be involved in neurodegenerative pathology. However, so far this has only been studied in vitro with SK-N-MC and Neuro2A cells (Sidharthan et al., 2013).

Levels of glucuronidated dopamine vary with sympathetic input to the periphery (Claustre et al., 1983; Alexander et al., 1984), although the mechanisms underlying this variability is unclear. Glucuronidation is common in the gut, and while human cells do not express glucuronidase enzymes, many gut bacteria express *β*-glucuronidase that can reverse this process (Pellock and Redinbo, 2017). In the gut lumen of mice, levels of free and glucuronidated dopamine are regulated by the activity of gut microbiota, such as *Clostridium,* with high levels of *β*-glucuronidase activity (Asano et al., 2012). More broadly, numerous bacterial species in the human gut can release catecholamines (Kim and Shin, 2018; Xue, Zhang et al., 2018; González-Arancibia et al., 2019), and dietary changes that alter the gut microbiome directly influence neurotransmitter levels in the brain and gut (Guo et al., 2021). Further, changes in gut microbial composition that reduced TH expression in the gut exacerbated invariant natural killer (NK) T-cell–mediated hepatitis (Xue, Zhang et al., 2018). Taken together, these data indicate that the microbiome plays an important role in the regulation of peripheral dopamine levels, potentially by modulating levels of conjugated dopamine. More research is needed to define how both sulfation and glucuronidation can impact peripheral dopamine levels, but these studies can be challenging as there are large differences in the extent of conjugation between different species (Claustre et al., 1983; Eisenhofer et al., 1997). Still, the active production, degradation, conjugation, and excretion of dopamine indicate an active peripheral dopaminergic system, but the importance of peripheral regulatory mechanisms, and indeed many of the activities of peripheral dopamine, remains understudied.

## II. Considerations and Caveats Regarding the Study of Dopaminergic Immunology

### A. Interpreting In Vivo Measurements of Dopamine

Despite an increasing focus on the immunomodulatory effects of dopamine, there is still a substantial gap in our knowledge regarding the specific impact of this neurotransmitter on different types of immune cells and diseases. Determining specific dopamine concentrations to use in vitro can be difficult, as dopamine concentrations in specific organ systems or to which immune cells could be exposed are not well defined. In animal models, studies of CNS dopamine levels are more common, particularly in rodents, and a number of studies have examined peripheral dopamine levels in animal systems, although to a much lesser extent than the brain. Direct access to a living human brain is limited, and although we can examine DAT and dopamine receptor densities, we are still developing the tools needed to define dopamine levels by neuroimaging in humans. While it is possible to directly measure peripheral dopamine levels, there has been relatively little interest in studying peripheral dopamine, so studies that make these determinations are scarce. Our recent review discusses these data in detail, aggregating studies to present the general ranges of dopamine levels throughout much of the CNS and periphery (Matt and Gaskill, 2020).

However, these ranges are relatively wide in most organs, with considerable differences within each region. A substantial amount of this variation is likely due to differences in populations, species, or environment between the different studies. However, some differences may also be due to specific technical issues that should be carefully considered when assessing dopamine concentrations. Measuring dopamine in postmortem tissue requires careful consideration of the isolation procedures used, as exposure to oxygen, metal ions, or specific enzymes can induce oxidation of the catechol ring and result in the production of dopamine quinones (Graham et al., 1978; Stokes et al., 1999; Sulzer and Zecca, 2000). This could reduce the amount of detectable dopamine present in the sample and potentially underestimate dopamine levels. Oxidation can also affect dopamine metabolites and may be more pronounced in certain regions, generating artificial distortions in dopamine:dopamine metabolite ratios and regional differences in dopamine levels and metabolism (Sloviter and Connor, 1977; Graham et al., 1978; Spokes and Koch, 1978; Kontur et al., 1994; Sun et al., 2018). Rapid tissue processing, processing under anoxic conditions (Morelli et al., 2011; Emanuel et al., 2022), and isolation and storage at colder temperatures can prevent oxidation and may provide a more accurate measurement of tissue dopamine levels.

Other technical considerations include interference by molecules with similar oxidation potential, and the sensitivity and specificity of the neurochemical techniques used for analysis, as this can have significant effects on the assessed dopamine concentration. Many of the methods from which our current understanding of anatomic dopamine levels has come (high-performance liquid chromatography, microdialysis or fast scan cyclic voltammetry) have considerable associated errors (McLaurin et al., 2021). More precise and potentially higher levels of dopamine (Patriarchi et al., 2018) will likely be measured as more studies use newer electrochemical detection techniques that are enhanced with aptamers or molecularly imprinted polymers and enzyme-, aptamer-, and antibody-based biosensors, as well as more sensitive in vivo tools including fiber photometry and genetic dopamine sensors such as dLight (Patriarchi et al., 2018; Leopold et al., 2019; Sabatini and Tian, 2020; Labouesse and Patriarchi, 2021; McLaurin et al., 2021).

Consideration must also be given to the relationship between the amount of dopamine measured in a particular tissue and the amount of dopamine to which immune cells in that compartment are exposed. In the brain and likely other compartments, biogenic amines and their metabolites are generally found in three regions: the extracellular space, cytoplasmic vesicles, and the cytoplasm itself (Best et al., 2009, 2010). Postmortem tissue collection and analysis limits the measurement of dopamine to that found within cytosolic and vesicular pools but not the extracellular pool as this is lost in processing, and most in vivo studies examine dopamine levels in the brain in close proximity to neurons. Further, the overall dopaminergic tone in a particular tissue does not account for microenvironments. These are created because dopamine levels throughout tissues are dynamic and based on the density and activity of cells that produce, take up, and degrade dopamine in that compartment (Kawagoe et al., 1992; Wightman et al., 2007). In many studies focusing on changes in dopamine in the context of behavior or neurotransmission, these concerns should be noted, but they are not likely to change the results, as it is the amount of change rather than baseline dopamine concentrations that are critical. However, in vitro studies or studies that expose immune cells to a specific amount of dopamine should consider these issues, as the amount of dopamine to which immune cells respond in vivo may only be approximated by the range determined for that tissue.

### B. In Vitro Dopamine Concentrations

To account for uncertainty in the physiologically relevant concentrations of dopamine, in vitro examinations of dopaminergic immunomodulation should use dopamine concentrations that approximate dopamine levels to which the immune cell being studied could be exposed. In most tissues, this range is likely to be approximately 10^−5^M to 10^−11^M, although the utility of higher and lower levels of dopamine should be determined based on the experimental question. While the higher end of dopamine concentrations are >10^−5^M in some brain regions and peripheral compartments such as the adrenal gland, gut, and carotid body, most immune cells will generally encounter lower concentrations of dopamine during homeostatic conditions (Matt and Gaskill, 2020). In addition, regulation of dopamine levels is likely to be disrupted by a number of diseases, as well as by many therapeutics, particularly neuropsychiatric drugs (Matt and Gaskill, 2019).

Of particular note, the dopamine levels encountered by immune cells in the CNS are very likely to be increased in the context of SUDs, as all addictive substances acutely increase CNS dopamine in the mesocorticolimbic system, as well as other brain regions (Di Chiara and Imperato, 1988; Pierce and Kumaresan, 2006; Volkow, Fowler et al., 2009). Data suggest that methamphetamine generally induces the greatest increase, resulting in concentrations of approximately 1 to 5 × 10^−5^M (Matt and Gaskill, 2020). The effects of neuropsychiatric and addictive drugs on peripheral dopamine are not clear and need further study, although whole-body positron emission tomography (PET) scanning in mice showed that cocaine, ketamine, and methamphetamine altered dopamine concentrations in several peripheral organs (Yeh et al., 2014). The major effect of exogenous increases in dopamine is likely to be an increase in the number of immune cells exposed to lower dopamine levels, as increased dopamine release expands the area of tissue exposed to dopamine, and lower concentrations cover a larger area and contact more cells (Peters and Michael, 2000; Venton et al., 2003; Spuhler and Hauri, 2013). Thus, even studying the immunoregulatory effects of stimulant-induced dopamine levels does not necessarily require the use of dopamine levels much higher than 10^−5^M.

The concern regarding the use of extraphysiologic dopamine concentrations (>10^−5^M) is that it may create confounding results by initiating immune functions that do not occur in vivo. One mechanism by which this could occur is through the activation of lower affinity dopamine receptors that would not be activated when an immune cell encounters lower dopamine levels. Different dopamine receptors, as well as nondopamine receptors, also have discrete affinities for dopamine (Richtand, 2006; Beaulieu and Gainetdinov, 2011). Thus, lower dopamine levels may activate high affinity receptors (D3, D4, and D5) to drive one function, while high dopamine levels could activate both the low- (D1 and D2) and high-affinity dopamine receptors, potentially activating other distinct functions through multiple receptors. For example, dopamine concentrations from 10^−10^M to 10^−6^M dose-dependently decrease glucagon secretion by human pancreatic islet cells, but 10^−4^ and 10^−5^M dopamine increases glucagon release (Aslanoglou et al., 2021). In splenic macrophages from wall lizards, lower dopamine levels (10^−11^–10^−15^M) increased phagocytosis, while higher dopamine levels (10^−7^–10^−5^M) decreased phagocytosis (Roy and Rai, 2004). In schizophrenia, it has been suggested that lower dopamine levels selectively stimulate high-affinity dopamine receptors (D3 and D5) and trigger inflammation, while high dopamine levels stimulate low-affinity dopamine receptors (D1 and D2), inducing an anti-inflammatory effect (Pacheco, 2017; Vidal and Pacheco, 2020).

Exposure to increased dopamine levels may also mediate effects through nondopamine receptors, such as adrenergic receptors, as higher concentrations of dopamine can bind to adrenergic receptors in different tissues and species (Cornil et al., 2002; Cornil et al., 2008; Lei, 2014; Ozkan et al., 2017; Aslanoglou et al., 2021), which can also drive inflammatory changes. For example, the *β*-adrenergic receptor antagonist propranolol inhibited dopamine-induced increases in nuclear factor kappa-light-chain-enhancer of activated B-cells (NF-*κ*B), interleukin (IL)-6, and IL-8 and blocked dopamine-mediated IL-12p40 suppression in human keratinocytes and rodent macrophages (Hasko et al., 2002; Parrado et al., 2012; Parrado et al., 2017). In the RAW264.7 rodent macrophage cell line, dopamine only affected nitric oxide (NO) production at a concentration of 5 × 10^−6^M, while much lower concentrations of the adrenergic receptor agonists norepinephrine and epinephrine increased lipopolysaccharide (LPS)-induced NO production, suggesting that dopamine may act through adrenergic receptors in this system (Chi et al., 2003).

Exposure to high levels of dopamine could also induce nonspecific changes in immune cell function through cytotoxicity and oxidative stress induced by the formation of dopamine quinones and other ROS (Graham et al., 1978; Stokes et al., 1999; Sulzer and Zecca, 2000). Studies in BV-2 microglia showed that quinone formation resulting from dopamine pre-treatment (10^−6^M–10^−4^M) for 24 hours attenuated LPS-induced expression of IL-6, tumor necrosis factor alpha (TNF-*α*), and IL-1*β* by inhibiting NF-*κ*B (Yoshioka et al., 2016, 2020). Treatment with 0.5 to 2 × 10^−5^M dopamine induced cell cycle arrest and apoptosis in rapidly dividing B-cells via oxidative stress (Meredith et al., 2006), and in peripheral blood lymphocytes (PBLs), 1 to 5 × 10^−4^M dopamine induced intracellular ROS levels and apoptotic cell death through oxidative stress. Similar effects have been seen in other studies of primary human lymphocytes, which showed increased apoptotic marker levels and dose-dependent decreases in proliferation, differentiation, and the synthesis of IL-4 and interferon gamma (IFN-*γ*) in response to high dopamine concentrations (10^−5^–5 × 10^−4^M) (Bergquist et al., 1994, 1997). In addition, the antioxidant glutathione prevented high dopamine levels (6–10 × 10^−5^M) from reactivating latent human immunodeficiency virus (HIV) in a chronically infected T-cell line (Scheller et al., 2000), indicating that reactivation was induced by oxidative stress. Thus, the use of extraphysiologic dopamine levels could produce confounding results by increasing the activation of receptors that may not be physiologically relevant and inducing aberrant effects due to cell death and the subsequent response to factors released from apoptotic cells.

### C. Evaluating and Accounting for Dopamine Receptor Expression

Cell type- and species-specific differences in dopamine receptor expression and signaling have important implications in the effects of dopamine on immune cell function and regulation. As has been reviewed recently (Levite, 2016; Pacheco, 2017; Pinoli et al., 2017; Matt and Gaskill, 2020) and will be discussed in subsequent sections, the majority of immune cells have been shown to express all types of dopamine receptors. Further, dopamine receptors are also expressed in several other nonneuronal cell types, such as renal proximal tubule cells (Dawirs and Teuchert-Noodt, 1992; Fardoun et al., 2007; Han et al., 2007), pancreatic beta cells (Rubi et al., 2005), brown adipocytes (Kohlie et al., 2017), and cells throughout the gut such as stomach parietal cells (Mezey et al., 1999; Li, Schmauss et al., 2006). However, the relative expression levels of the different dopamine receptor subtypes are not consistent between studies.

Dopamine receptor expression levels are typically analyzed at the mRNA level using in situ hybridization and reverse-transcription polymerase chain reaction or at the protein level by Western blotting, immunohistochemistry, or immunofluorescent staining. While mRNA expression does not equate to protein expression and cannot reveal the receptor expression levels in the plasma membrane, the absolute specificity of the probe/primer sequences can indicate expression, enable precise subtype differentiation, and suggest the relative ratios between the different subtypes. In contrast, protein analysis can theoretically demonstrate surface expression and be used to quantify receptor density. However, dopamine receptor antibodies often lack specificity (Bodei et al., 2009; Michel et al., 2009), likely due to the high homology among dopamine receptor subtypes within a given subfamily (Platania et al., 2012). Thus, antibodies may not sufficiently discriminate different receptor proteins on the cell surface, nor do they bind to denatured proteins in blotted membranes. Moreover, because the molecular sizes of dopamine receptor subtypes are relatively similar [D1, 49 kD; D5, 53 kD; D2 short isoform, 47 kD; D2 long isoform, 59 kD; D3, 44 kD; however, several shorter isoforms have been described (e.g., D4, 41 kD), although weight vary slightly based on the number of 48-base pair variable tandem repeats in exon 3] (Van Tol et al., 1992; Fishburn et al., 1993; Khan et al., 1998; Richtand, 2006; Beaulieu and Gainetdinov, 2011), their immunoblot signals could be, at least in part, superimposed (Bucolo et al., 2019).

These technical issues, as well as differences in the information provided by these methodologies, creates difficulties when trying to make comparisons across the literature. For instance, mRNA and protein expression levels of dopamine receptors vary greatly among human and rodent immune cell types, making comparisons across species complicated (Davis, 2008; Shay et al., 2013). Even within species, there can be large differences, as primary human macrophages have high expression of D1-like receptors and D2, but much lower expression of D3 and D4 (Nickoloff-Bybel et al., 2019; Nolan et al., 2019), while human THP-1 monocytic cells have substantially higher levels of D4, particularly relative to D1-like receptor expression (Basova et al., 2018). The murine immature osteoblast line, MC3T3-E1, only expresses D1 and D4, while all subtypes of dopamine receptors except D3 are expressed in primary osteoblasts (Motyl et al., 2017).

These differences are especially important when considering the impact of dopamine on cells isolated during different disease states or in activated and resting cells. Diverse stimuli, as well as many pathologies, can alter both dopamine concentrations and dopamine receptor expression levels, which could potentially activate different dopamine receptors than would normally respond to dopamine and promote important homeostatic activity. Changes in the expression of some dopamine receptors and not others could also affect function by altering the ratios of different dopamine receptors. As changes in dopamine receptors may be critical for determining the effects of dopamine on distinct cell types, it is important to define baseline expression levels, how those levels compare with other systems, and how those levels change throughout experiments. Moving forward, examining dopamine receptor ratios may be more relevant than measuring individual receptor expression. Further, examining receptor ratios in relation to other receptors that modulate dopamine signaling (e.g., adrenergic receptors) may be a better strategy for understanding dopamine-mediated patterns in immune function and disease.

### D. Species-Specific Dopamine Signaling and Immune Function

Classically, dopamine signaling has been defined in neurons, but several studies have shown dopamine signaling differs between neurons and immune cells, as well as other nonneuronal cell types (Wang et al., 2005; Beaulieu and Gainetdinov, 2011; Beaulieu et al., 2015), and between species. This is particularly important regarding rodent–human differences, as rodents and rodent immune cells are commonly used to model human immune function. There are substantial differences between rodent and human immune responses due to genetic differences and the experimental environment (Beura et al., 2016; Tao and Reese, 2017). For example, LPS amplifies toll-like receptor (TLR)2/6 responses and downregulates CXCR4 in murine macrophages but does not affect TLR2/6 responses and increases CXCR4 expression in human macrophages (Ariffin and Sweet, 2013). There are also differences in the transcriptional profiles of rodent and human immune systems (Shay et al., 2013), and studies of inflammatory diseases using rodents often poorly translate to humans (Mestas and Hughes, 2004; Seok et al., 2013).

This disconnection is also seen regarding dopamine. In human primary monocyte-derived macrophages (hMDMs), D1-like receptor activation does not stimulate cAMP production (Nickoloff-Bybel et al., 2019) and primes the nucleotide-binding oligomerization–like receptor family pyrin domain containing 3 (NLRP3) inflammasome (Nolan et al., 2020). However, studies in LPS-primed murine bone marrow–derived macrophages (BMDMs), D1 activation inhibits NLRP3 activity through a cAMP-dependent pathway, although D1-mediated increases in cAMP were not directly observed (Yan et al., 2015). Although both studies examined D1-like receptors, there are clear differences in the activated pathways. These variations may result from species-specific differences in dopamine receptor or inflammasome activity, the presence of LPS, or the different concentrations of dopamine used, which may have activated different dopamine receptors. Similarly, both human and mouse pancreatic *α*- and *β*-cells express dopamine and adrenergic receptors, but there are large, species-specific differences in the ratios of dopamine receptors to adrenergic receptors. Further, dopamine induces dose-dependent changes in glucagon in human islets, but low levels decrease glucagon, while higher levels increase glucagon. In contrast, dopamine only dose-dependently increases glucagon in mouse islets. This may be due to variations in the ratios of receptors with different dopamine affinities (Aslanoglou et al., 2021). These types of dissimilarities could, at least partially, explain species-specific differences in dopaminergic immunomodulatory effects. Differences in environmental stimuli could also contribute to these effects, such as the comparison of LPS-stimulated and nonstimulated cells (Gaskill et al., 2012) or between cell activity in vitro and in vivo.

Additionally, dopamine receptors can form heteromers with other dopamine receptors, other types of GPCR, and even ion channels. This includes D1–D2, D1–D3, D2–D3, D2–D4, and D2–D5 complexes (Marcellino et al., 2008; So et al., 2009; González et al., 2012; Perreault et al., 2014), although there has been controversy about the existence of D1–D2 receptors under physiologic conditions (Rashid et al., 2007; Chun et al., 2013; Frederick et al., 2015; Hasbi, Sivasubramanian et al., 2020). Dopamine receptors also oligomerize with many other types of GPCR, mostly receptors associated with neurotransmission. This includes, but is not limited to, adenosine, N-methyl-D-aspartate, corticotrophin-releasing hormone, neurotensin, serotonin, histamine, and metabotropic and ionotropic glutamate receptors (Borroto-Escuela et al., 2013; Cahill et al., 2014; Ferre et al., 2014; Fuenzalida et al., 2014; Moreno et al., 2014; Perreault et al., 2014; Andrianarivelo et al., 2021). Some studies show sex differences (Hasbi, Nguyen et al., 2020) in dopamine receptor heteromers, and there may also be cell type or species-specific differences in the type or frequency of dopamine receptor oligomers, although this has not been well studied. Heteromeric receptors often display distinct signaling capacity and functional selectivity in ligand binding (Ferre et al., 2014), and the formation of distinct types of heteromeric complexes may be associated with the frequency of different receptors on distinct cell types. Thus, immune cells may be more likely to generate heteromeric complexes between dopamine receptors and cytokine or chemokine receptors that enable dopamine to influence immune activity. For example, a recent study showed that D5R and C-C motif chemokine receptor 9 (CCR9) form a heteromer on both mouse and human CD4^+^ T-cells and that these D5R:CCR9 complexes are increased during gut inflammation and drive colonic homing of these T-cells (Osorio-Barrios et al., 2021). Thus, whether immune dopamine receptors exist as monomers or as part of an oligomeric complex could have substantial impact on the dopamine-mediated signaling processes and immune functions in those cells. These and other data clearly show that much more research is needed to effectively interpret and synchronize our understanding of the broad impacts of dopamine, particularly across species.

### E. Considerations Regarding the Effects of Dopamine Induced by Addictive Substances or Pharmacological Agents

Another concern when interpreting the immunologic effects of dopamine is the potential imprecision associated with the use of pharmacologic agents to activate dopamine receptors. Specificity of a pharmacologic agent is determined and/or relies on a distinct experimental system, so specificity may differ between systems with varying dopamine receptor levels, and more specific drugs or additional mechanistic studies may be needed to precisely target a particular receptor. This is problematic because when agonists and antagonists are not specific or selective enough, they do not provide the basis for the univocal and unambiguous identification of particular receptors (Salomone and Waeber, 2011).

For example, studies that treat immune cells in vitro with dopamine receptor antagonists often show modulatory effects. But if there is no dopamine in the system, it is not clear that the antagonist is preventing the effect that results from receptor activation. In these cases, antagonists may have effects on systems that are dopamine receptor–independent. Antagonists could also be acting on dopamine receptors in unexpected ways because the affinity for the dopamine receptors expressed in these systems are distinct from the system in which the antagonist was defined. It is also possible that the antagonist is blocking the effects of endogenous dopamine release and autocrine activation (we note in the next section that many immune cells produce and secrete dopamine as part of their communication strategy). Because it is often not clear how the pharmacologic agent is acting on the dopamine receptor being studied or what is the appropriate concentration of agonist/antagonist to use, extrapolations about the effects of dopamine based on pharmacologic drugs could be misleading.

Another similar issue occurs in studies evaluating the immunomodulatory effects of addictive drugs. As previously noted, all addictive substances, including stimulants such as cocaine and methamphetamine (Di Chiara and Imperato, 1988; Kimmel et al., 2005), alcohol (Wozniak et al., 1991; Kegeles et al., 2018), cannabis (Chen et al., 1993), or opioids such as heroin (Hemby et al., 1995), acutely increase CNS dopamine levels through distinct mechanisms of action (Pierce and Kumaresan, 2006; Volkow, Fowler et al., 2009). Further, many of these substances also act on other systems, such as the opioid or endocannabinoid system, or specific receptors such as sigma-1 (Tsai et al., 2015; Lever et al., 2016; Cai et al., 2017) or trace-amine associated receptor 1 (Cotter et al., 2015; Sriram et al., 2016). Thus, the in vivo immunomodulatory effects of these drugs could result from the dopamine released by the use of these substances or the interactions of the substances themselves with other receptors. While in vitro monocultures of immune cells can produce some dopamine, they lack the capacity to release dopamine as it is produced in vivo, and the presence and activity of additional receptors in these systems is often undefined. Thus, it is not necessarily accurate to attribute the immunomodulatory effects of addictive substances to dopamine when these effects are defined in vitro. As a result, many of these studies are actually evaluating the immunologic effects of the substances themselves rather than the dopaminergic impact of their use, and follow-up studies examining the specific receptors or signaling pathways induced by these substances could be fruitful. However, to fully address the immunomodulatory effects of dopamine induced when using addictive substances, in vitro mechanistic studies using the dopamine concentrations induced during SUDs should be combined with in vivo analyses to give a more complete picture of the impact of each addictive substance.

## III. Dopamine and Immune Cells

### A. Introduction

A growing body of evidence has shown that dopamine can modulate a variety of immune functions, including proliferation, chemotaxis, antigen presentation, phagocytosis, cytokine secretion, and cell adhesion (Cosentino et al., 1999; Sarkar et al., 2010; Levite, 2016; Nolan et al., 2018). Research defining the immunomodulatory effects of dopamine often generates conflicting data, suggesting that dopamine elicits activity or quiescence, or both pro- and anti-inflammatory states depending on the cell type, model system, and experimental context (Tarazona et al., 1995; Hasko et al., 1996, 2002; Sarkar et al., 2006; Capellino et al., 2010; Nakano et al., 2011; Gaskill et al., 2012; Franz et al., 2015; Yan et al., 2015; Zhang et al., 2015; Zhang, Jiang et al., 2016; Nolan and Gaskill, 2019; Yoshioka et al., 2020). Much of this research has focused on myeloid cells, particularly macrophages and microglia, and T-lymphocytes, although dopamine has been shown to affect immune function in most immune cell types.

Almost all types of immune cells express various levels of both the D1- and D2-like receptors, as well as other proteins involved in the synthesis, reuptake, transport, and metabolism of dopamine, such as DAT, TH, VMAT2, and MAOs (McKenna et al., 2002; Farber et al., 2005; Cosentino et al., 2007; Gaskill et al., 2009, 2012; Mastroeni et al., 2009; Kustrimovic et al., 2014; Coley et al., 2015; Huck et al., 2015; Levite, 2016; Nolan and Gaskill, 2019; Prado et al., 2021; Wieber et al., 2022). This suggests that most immune cells interact with dopamine through surface receptors and can take up dopamine through active transport. Uptake and release of dopamine can modulate both the releasing cell and neighboring cells, mediating a variety of functions, such as transforming growth factor (TGF)-*β* and IL-10 production or B-cell activation (Faraj et al., 1991; Cosentino et al., 2007; Pacheco et al., 2009; Arreola et al., 2016; Papa et al., 2017). It is not entirely clear whether the dopamine that mediates these interactions is produced de novo or taken up from the surrounding environment and released. However, studies inhibiting TH activity and measuring catecholamine levels in human neutrophils (Cosentino et al., 1999), lymphocytes (Musso et al., 1996; Qiu et al., 2005), peripheral blood mononuclear cells (PBMCs) (Marino et al., 1999; Cosentino et al., 2002), and B-cells (Honke et al., 2022) show changes in dopamine levels in immune cells, and follicular T-helper cells specifically produce and store dopamine in dense-core granules marked by chromogranin B (Papa et al., 2017). Stimulation of rodent macrophages and neutrophils with LPS also resulted in the production of catecholamines (Flierl et al., 2007). These studies indicate that immune cells produce small quantities of dopamine de novo, and many of these cells use the produced dopamine for autocrine or paracrine regulation by activating dopamine receptors on neighboring cells. The following sections will further discuss the dopaminergic systems in the immune cells of the CNS and periphery, as well as specific immune cell responses to dopamine signaling. Then, the bidirectional interaction of dopamine and inflammation will be reviewed, discussing the role of dopamine in driving inflammatory functions such as cytokine production and the impact of inflammation on dopaminergic machinery.

### B. Dopaminergic Machinery and Activity in CNS Immune Cells

Dopamine levels in dopaminergic regions of the CNS, including the striatum, VTA, NAc, and prefrontal cortex (PFC), are among the highest in the body. Therefore, CNS immune cells, particularly in these regions, are likely to frequently encounter immunomodulatory levels of dopamine, and dopamine-mediated effects may be associated with homeostatic function in these regions (Matt and Gaskill, 2020). The most common immune cells in the CNS are myeloid cells, particularly microglia, as well as other types of CNS macrophages (Herz et al., 2017). Microglia are dynamic, yolk sac–derived, tissue-resident macrophages that make up a unique myeloid population in the CNS parenchyma (Prinz et al., 2019). These cells interact with neighboring neurons and other glia physically and through the production of cytokines and neurotrophic factors. Microglia play a critical role in the maintenance of neuronal health, synaptic pruning, and the organization of neuronal circuits (Schafer et al., 2012; Schafer et al., 2013; Li and Barres, 2018). These cells also protect the CNS by surveilling the parenchyma for pathologic insults or infection and differentiate into various activation states depending on the pathologic stimuli detected (Ousman and Kubes, 2012). Notably, microglia are overrepresented in dopaminergic midbrain pathways relative to other brain regions, so these areas may be particularly sensitive to the inflammatory effects of dopamine (Kim et al., 2017; Treadway et al., 2019). Activated cells can induce the production of inflammatory modulators such as IL-1, NO, IL-10, TNF-*α*, superoxide, and prostaglandin E2 to promote and/or quell inflammation (Wolf et al., 2017).

All five dopamine receptor subtypes have been identified on human microglia (McKenna et al., 2002; Mastroeni et al., 2009), human microglial cell lines (Matt et al., 2021) and rodent microglial cells (Farber et al., 2005; Kettenmann et al., 2011; Huck et al., 2015; Kopec et al., 2018) although not every study detected all subtypes. There may be an age-associated effect, as cultured human microglia from elderly individuals did not express Drd5 mRNA despite the presence of mRNA for the remaining dopamine receptors (Mastroeni et al., 2009). In rodents, microglial dopamine receptors and transcriptomes (De Biase et al., 2017) vary among brain region and local environment (Kuric and Ruscher, 2014; Huck et al., 2015), although this has not been studied in primates. Rodent microglia have also been shown to express DAT and the metabolic enzyme COMT (Myohanen et al., 2010; Fan et al., 2018), but studies are still needed to define the full dopaminergic system present in human microglia.

Dopamine has several effects on microglia, many pertaining to neuroinflammation and the activation state of these cells. In wild-type mice, no D2 expression was detected in Iba-1+ microglia, but cerebral ischemia and the associated inflammation induced high levels of D2 expression in these cells (Huck et al., 2015). In a separate study, both D1 and D2 were present in resting murine microglia, but only D2 mediated anti-inflammatory changes through the expression of renin-angiotensin receptors. In contrast, in LPS-induced microglia, both D1 and D2 receptors mediated this effect (Dominguez-Meijide et al., 2017). Furthermore, microglial activation was reduced by global ablation of D2 in a murine 1-methyl-4phenyl-1,2,3,6-tetrahydropyridine (MPTP) model of PD, although this was likely through T-cell-mediated inflammation (Liu, Zhai et al., 2021).

**Fig. 3:**
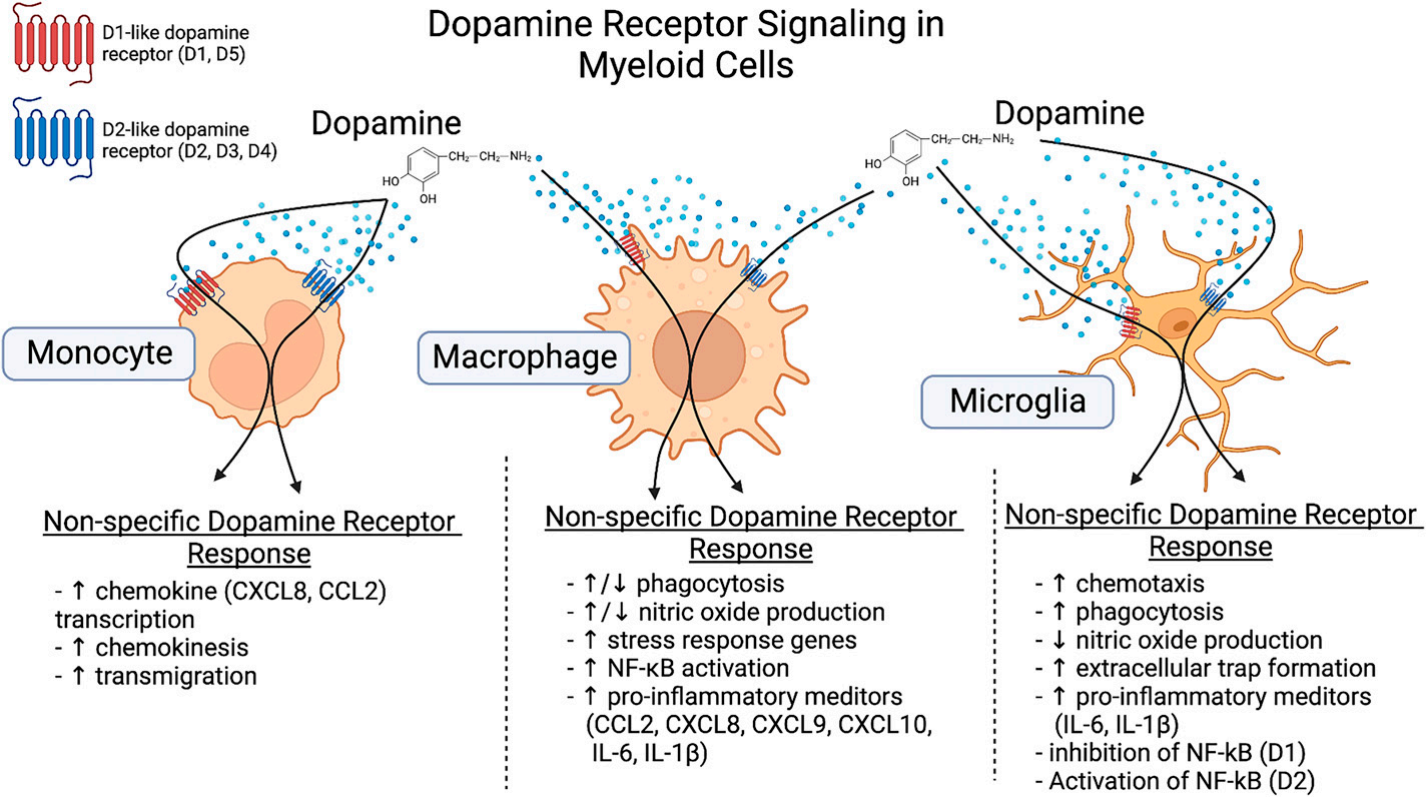
Dopamine receptor signaling in myeloid cells. Current knowledge of immunomodulatory effects of dopamine signaling in monocytes, macrophages, and microglia are summarized. In monocytes, dopamine signaling through the dopamine receptors leads to an increase in chemokine production, chemokinesis, and transmigration. Studies in macrophages show that dopamine can have bidirectional effects on phagocytosis and NO production, while stress response genes, NF-kB activation, and release of proinflammatory mediators are all increased in response to dopamine. In microglia, dopamine signaling through its receptors increases chemotaxis, phagocytosis, formation of extracellular traps and pro-inflammatory mediator production while decreasing NO production. Additionally, in microglia, in general D1-like receptor stimulation inhibits NF-kB while D2-like receptor stimulation activates NF-kB. Created with BioRender.com.

Studies in microglial cell lines and primary adult microglia show that with stimulation with phorbol myristate acetate or LPS, but not in unstimulated cells, dopamine (2.5 × 10^−6^M) induced the formation of extracellular traps. Although extracellular traps are normally generated in granulocytes, dopamine has not been shown to affect trap formation in that cell type. The novelty of this process was increased by the finding that these extracellular traps were formed independent of ROS production, actin polymerization, or cell death (Agrawal et al., 2021; Wu et al., 2021). This phenomenon may be particularly relevant to glioblastoma multiforme, a malignant brain tumor in which sterile neuroinflammation occurs along with release of dopamine (Agrawal et al., 2021; Alghamri et al., 2021; Roesler et al., 2021), suggesting that dopamine may be involved in the inflammation associated with this disease. There may also be sex-dependent differences in dopamine receptor expression associated with microglial functions. Microglial and complement-mediated phagocytosis both eliminate D1-like receptors in males but not females, and this elimination shapes NAc development (Kopec et al., 2018). These studies indicate that microglia express a fully functional dopaminergic system that may affect many microglial functions (summarized in Fig. 3). However, the effects of this system could vary widely depending on the brain region and local environment, and the specific role of dopamine in the function of human microglia in both health and disease requires further study.

In addition to microglia, there are other immune cell populations in the CNS, including peripheral immune cells such as T-cells and monocytes, a small number of which transiently survey the CNS during homeostasis (Prinz and Priller, 2017). The dopaminergic system in peripheral immune cells is discussed in subsequent sections. There are also many specialized CNS-resident macrophage populations, including perivascular macrophages, choroid plexus macrophages, and meningeal macrophages, although these cells are much less well studied than microglia. Classically, these macrophage populations were thought to be more closely related to peripheral macrophages than microglia, but fate mapping studies suggest that CNS macrophage populations are also yolk sac-derived and make up a stable, low-turnover population that is relatively transcriptionally related to microglia (Goldmann et al., 2016; Prinz and Priller, 2017). Further, recent studies suggest that some CNS macrophage populations are derived from the skull and vertebral bone marrow, and these populations occupy unique niches within the CNS (Cugurra et al., 2021). The functions of these specialized macrophage populations are not well defined, although major functions include sampling and removing various types of debris, providing trophic support to neurons and glia, and regulating the immune responses at different CNS boundaries such as the perivascular space, the choroid plexus, and the lymphatic and glymphatic systems (Herz et al., 2017). Although these cells are similar to microglia, it is not clear how much overlap is present between the dopaminergic systems in these specialized CNS macrophages and microglia.

Finally, while they are not considered immune cells, astrocytes are the largest population of cells in the CNS and play a central role in neuronal health and function, often having an immunomodulatory role in response to CNS insult or disease (Sofroniew, 2014; Giovannoni and Quintana, 2020). Like microglia, astrocytes can take on a reactive phenotype in response to CNS damage, inflammatory stimuli, and microglial activation (Hamby et al., 2012; Sofroniew, 2014; Liddelow et al., 2017; Giovannoni and Quintana, 2020). Activated astrocytes have both neuroprotective and neurotoxic effects, secreting both inflammatory and anti-inflammatory cytokines and chemokines that contribute to tissue repair and neuroinflammation (Markiewicz and Lukomska, 2006; Sofroniew, 2014; Giovannoni and Quintana, 2020; Linnerbauer and Rothhammer, 2020). An in-depth discussion of astrocytes is outside the scope of this review, but studies have shown that rodent astrocytes express all dopamine receptor subtypes (Bal et al., 1994; Zanassi et al., 1999; Reuss et al., 2000; Miyazaki et al., 2004; Montoya et al., 2019) as well as other dopaminergic proteins such as DAT (Takeda et al., 2002), monoamine oxidase B (MAO-B), and COMT (Fitzgerald et al., 1990; Myohanen et al., 2010; Winner et al., 2017). Astrocytes can also take up and metabolize dopamine (Pelton et al., 1981; Inazu et al., 1999), with at least one study indicating dopamine transport is regulated by the norepinephrine transporter (Takeda et al., 2002). Astrocytic dopamine receptors may play a role in inflammation (Shao et al., 2013; Zhang et al., 2015), as well as neuronal health and survival (Ohta et al., 2003, 2010; Li, Guo et al., 2006), indicating that dopamine responsiveness is important for glial cells within the CNS. There are species-dependent differences in astrocyte dopamine receptor expression and activity, and some studies have shown regional variations in dopamine receptor expression and responses. Astrocytes in dopaminergic regions such as the striatum, VTA or PFC express dopamine receptors and respond to dopamine, while those in other regions, such as the cerebellum, do not (Vermeulen et al., 1994; Khan et al., 2001; Reuss and Unsicker, 2001; Miyazaki et al., 2004; Xin et al., 2019). This finding indicates that the effects of dopamine on astrocytes, like many other cell types, is context- and environment-dependent.

### C. Dopaminergic Machinery and Activity in Peripheral Immune Cells

#### 1. Innate Immune Cells

The innate immune response is considered the first line of defense against invading pathogens and mediates rapid, nonspecific inflammatory responses. In innate cells, particularly myeloid cells, granulocytes, NK cells, and DCs, these responses are generally initiated by the exposure of extracellular and intracellular pattern recognition receptors (PRRs) to various stimuli associated with pathogens or other insults. The four main families of PRRs are toll-like receptors (TLRs), nucleotide-binding oligomerization domain-like receptors (NLRs), C-type lectin receptors, and RIG-1 like receptors (Janeway and Medzhitov, 2002; Takeuchi and Akira, 2010; Pinoli et al., 2017). TLRs are membrane proteins that are localized on endosomes and mediate extracellular recognition of pathogens, whereas NLRs are cytosolic proteins that recognize intracellular pathogens (Janeway and Medzhitov, 2002; Franchi et al., 2009; Kumar et al., 2011). There are currently 10 known functional TLRs in humans and 12 in mice, while there are 22 human NLRs (Kawai and Akira, 2008; Takeuchi and Akira, 2010). Formyl peptide receptors and scavenger receptors are also PRRs, as they bind N-formyl peptides produced by bacterial degradation and acetylated or oxidized low-density lipoproteins, respectively (Janeway and Medzhitov, 2002; Takeuchi and Akira, 2010; Pinoli et al., 2017).

PRRs act primarily by recognizing a variety of molecules known as pathogen-associated molecular patterns (PAMPs) or damage-associated molecular patterns (DAMPs), which signal the presence of danger. Examples of PAMPs and DAMPs include LPS, which is a component of Gram-negative bacterial cell walls; single- or double stranded RNA, which is associated with viral infection; *β*-glucans, which are components of fungal cell walls; and immunostimulants, such as polyinosinic:polycytidylic acid, which mimics activation caused by viral RNA. The expression and activation of PRRs on innate immune cells mediates coordinated effector responses upon contact with an invading pathogen (Janeway and Medzhitov, 2002; Li and Wu, 2021). Dopamine receptors and other dopamine-related proteins have been detected on most of these cells, suggesting the potential for broad effects of dopamine on many branches of the innate immune system (Pinoli et al., 2017).

##### a. Monocytes and Macrophages

Monocytes are myeloid cells that circulate through the blood and lymphatic system and can be rapidly recruited to sites of tissue damage and infection. Within tissues, these cells differentiate into macrophages, which secrete inflammatory cytokines and trophic factors, engulf and eliminate pathogens, regulate development and homeostasis, and mediate tissue repair/wound healing, thereby playing major roles in protective immunity. The influence of dopamine on myeloid cell activity is show in Fig. 3. Monocytes and macrophages in humans and other mammals express all dopamine receptor subtypes, as well as other dopamine-related proteins including DAT, VMAT2, TH, AADC, COMT, and both MAO-A and -B (Tarazona et al., 1995; McKenna et al., 2002; Brown et al., 2003; Liang et al., 2008; Gaskill et al., 2009, 2012; Coley et al., 2015; Bone et al., 2017; Nolan et al., 2019). Studies show that primary human (Cosentino et al., 1999; Mackie et al., 2022) and rodent myeloid cells (Flierl et al., 2009), as well as rice stem borer (*Chilo suppressalis*) hemocytes, blood cells that are analogous to macrophages in vertebrates (Wu et al., 2015), can take up, store, and produce dopamine.

The capacity of macrophages to synthesize dopamine is supported by studies that show that intracellular dopamine levels in myeloid cells are decreased by the catecholamine synthesis inhibitor alpha-methyl-p-tyrosine (Freeman et al., 2001) and that M2-polarization in rodent macrophages may reduce dopamine levels in these cells (Fischer et al., 2017). Both the monocytic murine cell line RAW264.7 and the human myeloid cell line U937 store dopamine and express L-DOPA decarboxylase, and LPS stimulation of RAW264.7 cells increased TH mRNA expression and intracellular dopamine within 48 hours (Brown et al., 2003; Kokkinou et al., 2009). The expression of TH and VMAT2 in U937 cells is only seen in response to LPS, suggesting that dopamine production in this cell line could be tied to activation (Capellino et al., 2010). This is also seen in human monocytes, where stimulation with TNF-*α* increases the number of TH^+^ cells and the amount of TH present in each cell (Gopinath et al., 2021). Notably, hMDMs do not express dopamine-*β*-hydroxylase (Nolan and Gaskill, 2019), indicating that dopamine cannot be converted to norepinephrine and that it is produced for use as dopamine.

In addition to myeloid cells in the blood, there are many tissue-specific macrophages, heterogeneous populations of myeloid cells that fulfill niche-specific functions. These populations include but are not limited to a variety of CNS myeloid cells like microglia and meningeal macrophages (brain), alveolar macrophages (lung), Kupffer cells (liver), adipose-associated macrophages (adipose tissue), Langerhans cells (skin), osteoclasts and bone marrow macrophages (bone), and intestinal macrophages (gut). There is very little specific data on the effect of dopamine on tissue-specific macrophages other than microglia, although recent studies have identified DAT on a subset of gut macrophages in the lamina propria (Mackie et al., 2022). This lack of information is likely because identifying, isolating, and working with specific tissue macrophage populations is technically challenging, and many studies on macrophages, including the vast majority of those examining the dopaminergic system, are done with hMDMs or BMDMs matured in vitro. While the use of hMDMs and BMDMs has and continues to provide valuable data regarding macrophage function, it is important to recognize that these cells are distinct from tissue-specific macrophages in many ways (Davies et al., 2013; Gordon and Pluddemann, 2017). Further, dopamine increases the expression of stress response genes such as hypoxia inducible factor-1a and nuclear factor erythroid 2-related factor in murine BMDMs via the uptake of unbound iron (Dichtl et al., 2018). This finding suggests that dopamine influences many more macrophage functions than are currently understood. Future studies examining the impact of dopamine on tissue-specific macrophages, as well as on general macrophage function, should account for these differences and improve understanding of the specific impact of tissue macrophages on homeostasis and disease.

##### b. Granulocytes

**Fig. 4:**
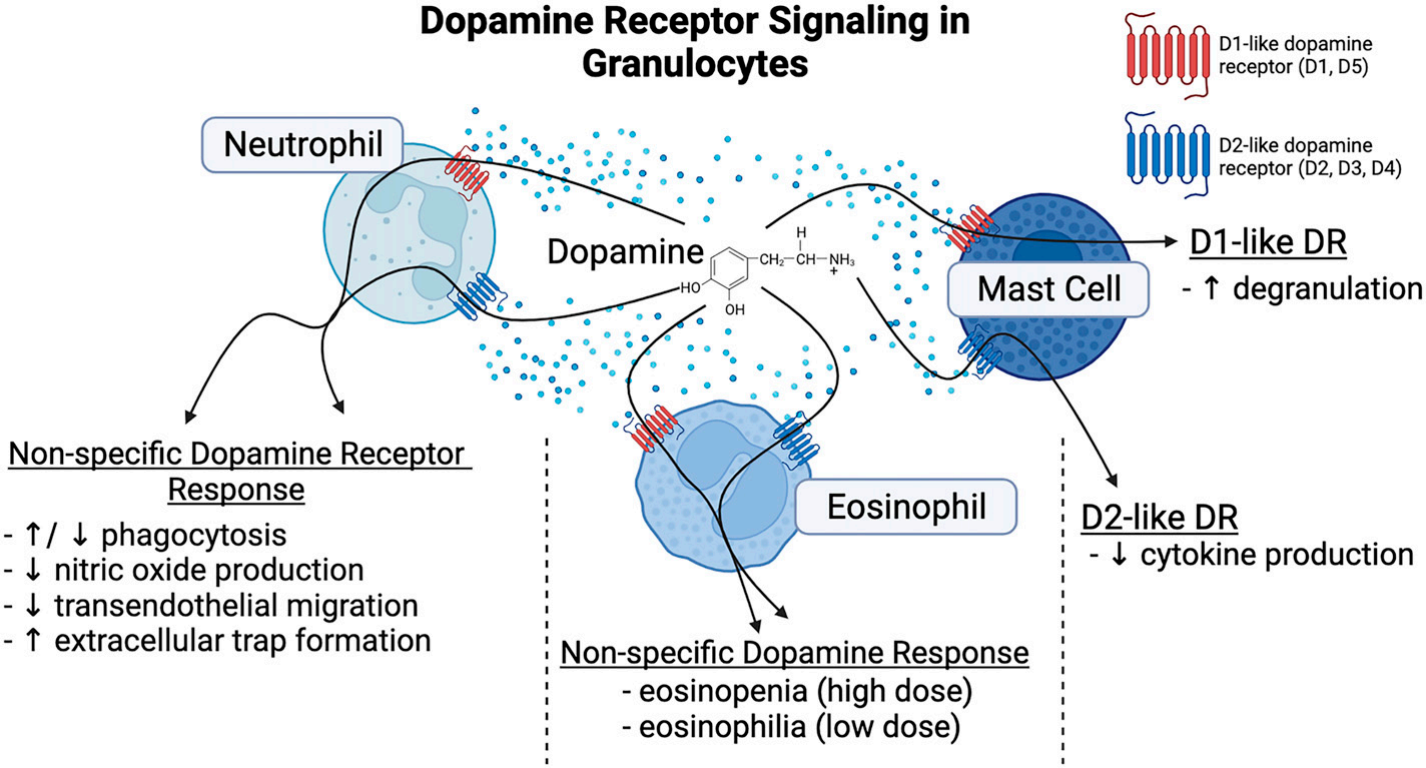
Dopamine receptor signaling in granulocytes. Current knowledge of the immunomodulatory effects of dopamine signaling in neutrophils, eosinophils, and mast cells is summarized. In neutrophils, dopamine acts through its receptors to increase formation of extracellular traps and to decrease NO production and transendothelial migration, while having reported bidirectional effects on neutrophil phagocytosis. Dopamine signaling in eosinophils has been shown to affect eosinophil counts with high doses of dopamine leading to eosinopenia and low doses of dopamine leading to eosinophilia. In mast cells, dopamine acting at D1-like receptors has been shown to increase degranulation while D2-like receptor stimulation decreases cytokine production. Created with BioRender.com.

There has been relatively little research on the impact of dopamine on the four types of granulocytes: neutrophils, basophils, eosinophils, and mast cells. Dopamine has been shown to mediate functional changes in neutrophils, eosinophils, and mast cells (Fig. 4) with little current research on basophils. The majority of the research has been studied in neutrophils (Pinoli et al., 2017). These cells are relatively short-lived granulocytes derived from the bone marrow that quickly move into the blood. Neutrophils are the most abundant granulocyte in circulation (40%–70% of blood leukocytes) and are often the first line of host defense, inhibiting or eliminating invading pathogens through the generation of cytokines, ROS, neutrophil extracellular traps, and other factors. Recent studies have shown that neutrophils play a critical role in communication between the innate and adaptive immune response through direct interactions or cytokine signaling (Rosales, 2020). The expression of both D1-like and D2-like receptors on human neutrophils has been confirmed at both the mRNA and protein levels (Cosentino et al., 1999; Sookhai et al., 1999; McKenna et al., 2002; Pereira et al., 2003; Chen et al., 2014).

Dopamine regulates neutrophil activity, as dopamine treatment (2.61 × 10^−7^ M) attenuated the expression of CD62l (L-selectin) and CD11b in neutrophils stimulated with N-formyl-methionyl-leucyl-phenylalanine, a potent neutrophil activator that mimics the actions of factors released by bacteria. This effect was not seen in resting neutrophils or in response to lower dopamine concentrations (2.61 × 10^−10^M) (Trabold et al., 2007). The downregulation of L-selectin and CD11b could substantially interfere with adherence to and rolling on activated endothelium, which is a primary function of these cells. Higher levels of dopamine (10^−4^M–10^−5^M) increased apoptosis in human neutrophils (Sookhai et al., 1999; Aslan et al., 2011), and the dopamine receptor antagonists chlorpromazine and pimozide blocked the increases in neutrophil counts associated with exposure to ovalbumin peptide in rats. In contrast, the dopamine receptor agonist apomorphine increased neutrophil numbers (Altenburg et al., 1995), suggesting that dopamine receptor activity could regulate neutrophil numbers and function and that higher levels of dopamine reduced neutrophil numbers, while lower levels may increase them.

Eosinophils are another granulocyte subset that are much less common than neutrophils (2%–3% of blood leukocytes) that have a key role in defense against helminth infection, injured tissue repair, and allergic diseases. These cells are released into the circulation like neutrophils but generally reside in tissues, mediating their effects through the release of cytoplasmic granules, cytokines, chemokines, and growth factors (Aoki et al., 2021). Very few studies have examined the dopaminergic system in these cells, but they have been shown to express all five subsets of dopamine receptors on their surface (McKenna et al., 2002). Eosinophils also express TH and can synthesize and release dopamine. Dopamine synthesis is slightly increased following activation with IL-5 and eotaxin, and inhibiting eosinophil TH activity with alpha-methyl-p-tyrosine decreased murine vascular relaxation in vitro. This suggests that the release of dopamine or other catecholamines is important for eosinophil function (Withers et al., 2017). In rats, administration of low-dose L-DOPA or apomorphine increased blood eosinophil count, while higher doses of these agents decreased blood eosinophil count, suggesting that dopamine synthesis and dopamine receptor activity affects eosinophil viability (Podolec et al., 1979). The usefulness of these findings is limited by our understanding of the dopaminergic system in eosinophils and is an area in need of further study.

Mast cells are myeloid lineage cells that originate in the bone marrow, but unlike other granulocytes, these cells reside in regions that are exposed to the external environment, such as mucosal and epithelial tissues. Mast cells are also common in connective tissues, and the many types of mast cells depend on the tissue environment in which they reside. These cells are important effectors of host defense against bacteria, venomous toxins, and triggers of allergic responses and anaphylaxis. During the immune host response, these cells release factors that rapidly recruit other innate and adaptive immune cells, which creates a balanced response to infection. Mast cells contain granules that store a variety of inflammatory mediators including cytokines and chemokines such as IL-4, C-C motif chemokine ligand 2 (CCL2), C-C motif chemokine ligand 5, and TNF-*α*; growth factors such as TGF-*β* and vascular endothelial growth factor; proteoglycans, proteases, and other enzymes; and biogenic amines such as histamine, serotonin, and dopamine (DeBruin et al., 2015). Mast cells express some dopamine receptors, and several studies indicate that dopamine plays an important role in mast cell function. Human synovial mast cells from patients with rheumatoid arthritis (RA) express D3, although these receptors were only seen on a subset of mast cells (Xue, Li et al., 2018). mRNA for D1 and D5 receptors was found in bone marrow and fetal skin derived mast cells (Mori et al., 2013). Bone marrow-derived murine mast cells also express TH and store dopamine in granules via a process that seems to be dependent on the presence of serglycin, an intracellular proteoglycan. Degranulation and the release of intracellular dopamine may be triggered by intracellular Ca^2+^ flux, which is mediated by IgE crosslinking (Ronnberg et al., 2012). Other studies have confirmed dopamine storage in murine (Freeman et al., 2001) and bovine mast cells (Edvinsson et al., 1977).

The release of dopamine is important for mast cell function, as dopamine-mediated (10^−9^M–10^−7^M) activation of D1-like receptors dose-dependently increased murine mast cell degranulation. Antagonizing D1-like receptors with SCH23390 also reduced ear swelling caused by passive cutaneous anaphylaxis, which is a mast cell–mediated process (Mori et al., 2013). Although not all D2-like receptors are present on mast cells, treating the rat cell line RBL-2H3 with bromocriptine, 7-OH-DPAT, haloperidol, and clozapine resulted in potent dose-dependent inhibition of degranulation (Seol et al., 2004). These ligands all bind to D2-like dopamine receptors, including D3, suggesting that D2-like receptors may also affect mast cell activity, although this cell type is actually derived from basophils, and it is not clear if it is entirely representative of mast cell biology (Passante and Frankish, 2009). Other studies suggest that D3 activity may be anti-inflammatory in mast cells, as there is a negative correlation between the numbers of D3-expressing mast cells in synovial fluid and disease severity in RA (Xue, Li et al., 2018). Furthermore, studies using bone marrow–derived mast cells from Drd3 knockout mice showed that D3 regulated the inhibitory effects of methamphetamine on LPS-induced cytokine production in mast cells. As methamphetamine does not bind to dopamine receptors, these data suggest that methamphetamine facilitates dopamine release in these cells by activating an autocrine signaling pathway by which the mast cells activate D3 to block inflammation (Xue et al., 2016). Taken together, these findings suggest that D1-like receptors may drive inflammatory activity in mast cells, while D2-like receptors may act in an anti-inflammatory manner.

Basophils are the largest and least common type of granulocyte, making up less than 1% of blood leukocytes. Basophils are very similar to mast cells but are found in the circulation rather than in tissues. After entering the peripheral blood, basophils transmigrate to various organs, such as the spleen, lymph nodes, and other inflammatory sites, where they can be activated. Like all granulocytes, these cells regulate innate inflammation and specialize in fighting parasites and regulating allergic responses. Basophils store inflammatory mediators such as IL-4 and TNF-*α*, as well as histamine, releasing them upon IgE crosslinking with surface receptors (Schwartz et al., 2016). The expression of dopamine receptors and other dopamine-related proteins on basophils is largely unclear, although human basophils do express VMAT2 (Anlauf et al., 2006), suggesting these cells can store monoamines in cytoplasmic vesicles. While there are no specific data on the functional effects of dopamine in this population, treatment with very low doses of epinephrine reduced histamine release (Mannaioni et al., 2010), and serotonin exposure significantly decreased IL-4 release (Schneider et al., 2011). These findings suggest that monoamines can regulate basophil function and that dopaminergic modulation may occur in regions with sufficient dopamine. For example, murine basophil activation leads to the release of serine proteases such as mMCP-8 and mMCP-11, which enhance microvascular permeability, allowing T-cells and other immune cells to transmigrate to sites of inflammation (Miyake and Karasuyama, 2017; Yamanishi et al., 2017). Dopamine has a known role in vascular regulation (Bhattacharya et al., 2008) and has been detected in the spleen and lymph nodes, which basophils frequent (Matt and Gaskill, 2020), suggesting that these cells are involved in this process. These interactions are currently unclear because of the lack of research on dopamine signaling in basophils, preventing a more comprehensive understanding of the role of dopamine in diseases characterized by basophilia, such as allergy or myeloproliferative disorders.

##### c. Natural Killer Cells

NK cells are granular lymphocytic cells that are often described as being in the gap between innate and adaptive immunity. These cells are named for their capacity to kill virus-infected and tumor cells without priming via antigen presentation or the recognition of major histocompatibility complexes (MHC) on target cells. Indeed, one of the primary functions of these cells is to recognize and eliminate other cells that lack MHC class I molecules. NK cells act by producing and secreting cytokines and chemokines that can influence the immune response and/or induce death pathways in infected cells (Ljunggren and Karre, 1990; Biron et al., 1999; Capellino et al., 2020). A variety of neurotransmitters, including dopamine, have been shown to influence NK cell function (Capellino et al., 2020), and human NK cells highly and consistently express all five dopamine receptors (McKenna et al., 2002; Zhao et al., 2013; Mikulak et al., 2014).

Dopamine may have both inhibitory and activating effects on NK cells. In NK cells isolated from human PBMCs, activation with IL-2 increases the expression of D5, allowing low levels of dopamine (10^−9^–10^−12^M) to inhibit cellular proliferation and reduce the synthesis of IFN-*γ* (Mikulak et al., 2014). In contrast, in NK cells isolated from mouse spleens, SKF38393 (D1-like agonist) treatment increased D1-like receptor expression and signaling through the cAMP–PKA–CREB pathway, enhancing cytotoxic activity against YAC-1 lymphoma cells. This study also showed that quinpirole (D2-like receptor agonist) reduced the expression of D3 and D4, as well as cytotoxic activity, and that antagonizing D2-like receptors with haloperidol blocked this effect (Zhao et al., 2013). Haloperidol also decreased NK cell activity in a different murine model (Nozaki et al., 1996), and in APO-SUS rats, which have a hyperreactive dopaminergic system characterized by increased expression of TH mRNA and D2 receptors, there is decreased splenic NK cell activity (Teunis et al., 2004). While this finding suggests that D2-like receptors generally suppress NK activity, one study assessing the effects of seven different dopamine receptor inhibitors on NK activity showed that only the pan-dopamine receptor antagonists thiothixene, fluphenazine, and trifluoperazine suppressed murine splenic NK cell cytotoxicity and effector-target cell conjugation (Won et al., 1995). This finding suggests that activation of multiple types of dopamine receptors or crosstalk with other receptors is needed to suppress NK activity or that these agents may affect NK cell immune function through alternative, nondopaminergic mechanisms.

##### d. Dendritic Cells

**Fig. 5:**
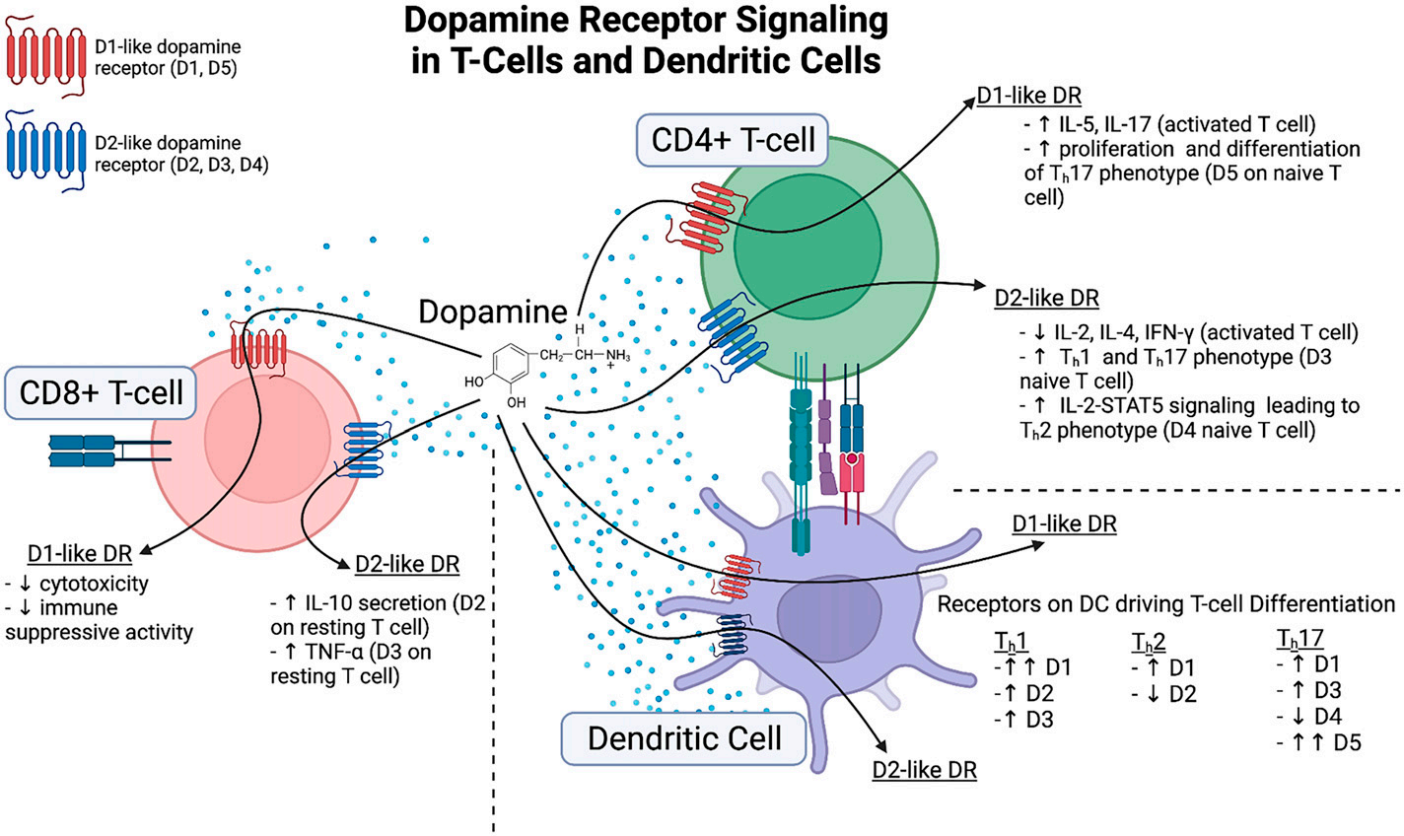
Dopamine receptor signaling in T-cells dendritic cells. Current knowledge of dopaminergic immunomodulation in dendritic cells and T-cells. Dopamine receptor balance on dendritic cells appear to impact T-cell differentiation. Increased expression of D1, D2, and D3 receptors on dendritic cells induces a T_h_1 phenotype in T-cells. Increased D1 expression with low D2 expression drives T_h_2 phenotype while increased D1, D3, and D5 along with low D4 expression has been linked to T_h_17 phenotype. Double arrows before receptor expression indicate a stronger correlation of the corresponding receptor on dendritic cells that drive T-cell differentiation. On activated CD4^+^ T-cells, dopamine stimulates D1-like receptors to increase IL-5 and IL-17 production and stimulates D2-like receptors leading to reduced IL-2, IL-4, and IFN-y production. On CD8^+^ T-cells, dopamine stimulation of D1-like receptors leads to decrease cytotoxicity and decreased immune suppressive activity. By acting on D2-like receptors on resting CD8^+^ T-cells, dopamine increases IL-10 and TNF-*α* production. Created with BioRender.com.

Dendritic cells (DCs) are professional antigen-presenting cells derived from both myeloid and lymphoid progenitors. The primary function of these cells is to migrate to secondary lymphoid organs and interact with T-cells to promote the differentiation of various effector T-cell subsets (Patente et al., 2019). Both human and rodent DCs express all dopamine receptor subtypes and other dopamine-related proteins such as MAO-A and -B, TH, and VMAT2. As in macrophages, dopamine-*β*-hydroxylase is not expressed in DCs, and interestingly, DAT was also not found, suggesting that these cells may not be able to take up dopamine from the extracellular space (Chen, Tsai et al., 2012; Prado et al., 2012; Figueroa et al., 2017; Arce-Sillas et al., 2019). Human DCs can produce dopamine de novo, and intracellular dopamine levels increase in response to synthetic L-DOPA. Dopamine production seems to be regulated by D2-mediated cAMP signaling that activates TH, as D2 blockade and treatment with forskolin, an activator for adenylate cyclase, increased stored dopamine. By inhibiting D2, dopamine signals predominately through D1-like receptors, which canonically stimulate the formation of cAMP (Beaulieu and Gainetdinov, 2011), increase phosphorylation of TH, and increase dopamine production (Nakano et al., 2009). Once produced, dopamine is stored in vesicles near the plasma membrane, which release dopamine. It is possible that the release acts in an autocrine fashion, binding to DC dopamine receptors to regulate proper dopamine levels, as dopamine is important in their role in antigen presentation and T-cell activation. These and other effects of dopamine on DC activity are summarized in Fig. 5.

When DCs interact with CD4^+^ T-cells, dopamine is released and activates D1-like receptors to increase cAMP signaling, which regulates T-helper (T_h_)1–T_h_2 polarization (Nakano et al., 2009). However, D2 may also be involved in this process, as blocking D2-like receptors in human monocyte-derived DCs with risperidone but not haloperidol inhibited DC production of T_h_1 cytokines (IL-6, IL-8, TNF-*α*). Blocking D2-like receptors also increased T_h_2 cytokine (IL-10) production and reduced T_h_1 polarization in cocultured T-cells (Chen, Tsai et al., 2012). Interestingly, haloperidol did affect the activity of murine bone marrow–derived DC, reducing the expression of MHC class II, CD80, and CD86, and decreasing the production of IL-12p40, an important factor for DC maturation (Matsumoto et al., 2015). This finding suggests species-specific differences in receptor crosstalk on murine and human DC, as both haloperidol and risperidone have strong affinities for D2-like receptors, but risperidone has much higher affinity for serotonin receptors.

Several studies from the Pacheco group indicate that the specific D1-like receptor that mediates DC–T-cell interactions is D5, as D5-mediated increases in cAMP increase the activation of signal transducer and activator of transcription and drive inflammation (Prado et al., 2018). The LPS-induced maturation of murine DCs decreased D5 expression, which impaired the activation and proliferation of antigen-specific CD4^+^ T-cells in vitro. Transplanting these D5-deficient DCs into a mouse showed a significant reduction in the percentage of T_h_17 cells infiltrating the CNS compared with that in wild-type animals (Prado et al., 2012). In the experimental autoimmune encephalomyelitis (EAE) mouse model of multiple sclerosis (MS), adoptive transfer of D5-deficient DCs reduced the severity of EAE and the frequency of inflammatory CD4^+^ T-cell subsets in the CNS. Reserpine-mediated dopamine depletion in wild-type DCs prior to transfer into EAE animals also reduced clinical severity, indicating that autocrine dopamine activation of D5 on DCs mediated the inflammatory effects of dopamine in this system (Prado et al., 2018). Because SCH23390 also blocks D5 activity, these data are supported by a separate study in which the inhibition of D1-like receptors with SCH-23390 blocked DC-mediated T_h_17 differentiation in EAE mice, although treatment with the D4-selective antagonist L750667 induced T_h_17 differentiation (Nakano et al., 2008). Adoptive transfer of D3-deficient DCs into mice had no effect on CD4^+^ T-cell responses but increased antigen cross presentation and CD8^+^ T-cell activity against tumors (Figueroa et al., 2017). This finding indicates that different dopamine receptors have discrete roles in antigen presentation and T-cell activation-associated signaling pathways in DCs. Because changes in antigen presentation impact the ability of the adaptive immune system to create memory, defining the overlapping and discrete dopamine pathways by which DCs regulate T-cell responses could be of substantial value in the treatment of autoimmunity.

#### 2. Adaptive Immune Cells

The primary cells that mediate adaptive immunity are T- and B-lymphocytes, which interact with the innate immune system to generate specific responses to invading pathogens, killing infected cells and generating specific antibodies against pathogens. A critical feature of adaptive immunity is immune memory, which enables the body to rapidly respond to repeat insults using factors that specifically target those pathogens. Lymphocytes, particularly T-cells, can differentiate into numerous subpopulations that have a wide variety of functions depending on the maturation process and stimuli to which they are exposed. The major T-cell subsets are classified by surface expression of CD4 or CD8. The CD4^+^ “helper” T-cells coordinate the immune response, activating other adaptive immune cells, such as memory B-cells and effector T-cells, and innate immune cells, such as macrophages. These cells are activated by exposure to peptide antigens on the surface of antigen-presenting cells, such as DCs and macrophages and differentiate into T_h_ subtypes that rapidly proliferate and secrete various cytokines. The subsets into which CD4^+^ T-cells differentiate include T_h_1, T_h_2, T_h_17, and others depending on the stimulus encountered and the type of immune function they need to execute. The CD4^+^ regulatory T-cell (T_reg_) subset primarily prevents a response to self-antigens and suppresses detrimental immune responses. CD8^+^ cells, also called cytotoxic T-cells or cytotoxic T lymphocytes, are activated by CD4^+^ T-cells or antigen-presenting cells, producing cytotoxins such as perforin and granzyme, Fas ligand, and various cytokines that destroy target cells such as virus-infected cells or tumor cells.

The other major lymphocyte that mediates adaptive immunity is the B-cell, which primarily produces antibodies, although B-cells can also act as antigen-presenting cells and produce cytokines. Naïve or memory B-cells express B-cell receptors on the plasma membrane, and when this receptor is activated by a specific antigen, the B-cell proliferates and differentiates into a plasma cell, producing large quantities of specific antibodies to fight invading pathogens. Both T-cells and B-cells can develop into memory cells that can persist in a quiescent state for long periods of time until activated by the specific antigen to which they respond. Once the memory cell is activated, it generates a rapid adaptive immune response. Lymphocyte differentiation, the specific roles of lymphocytes subsets, and the formation of immune memory are central to the adaptive immune response, and this topic is an extraordinarily expansive and well-researched field, with numerous excellent reviews (Murphy et al., 2008; Chaplin, 2010; Kurosaki et al., 2015; Netea et al., 2020). The role of dopamine in adaptive immunity has also been well described, and many studies and reviews have discussed the different functions dopamine can modulate in T-lymphocytes (Pacheco et al., 2009; Gaskill et al., 2013; Levite, 2016; Levite et al., 2017), although there has been much less study of B-cells (Tsao et al., 1997; Meredith et al., 2006).

##### a. T-Lymphocytes

T-cells are among the most well-characterized immune cell types associated with dopaminergic regulation and have been reviewed in detail, so we will discuss them more briefly. All dopamine receptor subtypes are expressed on the T-cell surface (Levite et al., 2001; McKenna et al., 2002; Besser et al., 2005; Kustrimovic et al., 2014). The binding profiles of dopamine receptor-specific ligands in these cells is similar to those observed in neuronal membranes, suggesting that the receptors may act similarly to those found in neurons (Takahashi et al., 1992). T-cells also express TH, DAT, VMAT2, and COMT, and different types of T-cells can synthesize, store, release, and take up dopamine (Bergquist et al., 1994; Josefsson et al., 1996; Cosentino et al., 2007; Papa et al., 2017), although the dopamine concentrations involved in these processes are not the same among T-cell subsets. For example, human CD4^+^CD25^+^ T_regs_ contained significantly higher dopamine levels than CD4^+^CD25^−^ T-cells (Cosentino et al., 2007), and human T follicular helper cells (T_f_h) stored much higher concentrations of dopamine than naïve T-cells (Papa et al., 2017). Some studies suggest that T-cells tightly regulate intracellular dopamine concentrations through a balance of synthesis, uptake and release (Bergquist et al., 1994). Treatment of lymphocytes with IFN-*β*, extracellular dopamine (10^−6^M–10^−8^M), or the PKC activator phorbol myristate acetate increased the production and release of dopamine and other catecholamines (Musso et al., 1996; Ferrari et al., 2004; Cosentino et al., 2005). The effects of dopamine on T-cell activity and maturation are summarized in Fig. 5.

As many of these effects are also distinct between T-cell subsets, the effects of dopamine are likely influenced by distinct dopamine receptor profiles. For example, D1-like receptors are highly expressed in both naïve and memory T-cells, while D2-like receptors are expressed mainly in memory T-cells and only marginally in naïve cells (Nakano et al., 2008; Mignini et al., 2013). The density of D1 expression is also significantly lower in human CD4^+^CD25^+^ T_regs_ than in CD4^+^CD25^−^ T-cells (Cosentino et al., 2007), and there is significantly higher D3 expression in CD8^+^ T-cells than in CD4^+^ T-cells (Watanabe et al., 2006). In rat thymocytes, the expression of dopamine-associated proteins such as DAT and VMAT2 was higher in CD8^+^ cells than CD4^+^ cells, and DAT and D1-like receptors are expressed at higher levels than D2-like receptors on rat thymocytes and rat peripheral lymphocytes (Mignini et al., 2013). In cells derived from the blood of individuals with PD, the levels of D1-like receptors are increased in total CD4^+^ T-cells and naïve CD4^+^ T-cells relative to those of healthy patients, and a decrease in D1 expression was associated with increased motor dysfunction (Kustrimovic et al., 2016). The variations in dopamine receptor expression and dopamine levels across T-cell subsets, as well as the release of dopamine in response to multiple types of stimuli, suggest that dopamine influences multiple T-cell functions and that this depends on the type of T-cell, the activation state, and local environment.

For example, in mice, dopamine (10^−5^ and 10^−7^M) downregulates the suppressive activity of T_regs_ but does not affect the D1-mediated response of T-effector cells to regulatory cell suppression, which may be mediated by ERK signaling (Kipnis et al., 2004). D1-like receptor expression was also associated with reduced T_reg_ function in human CD4^+^ T_regs_ (Cosentino et al., 2018). However, adoptive transfer of T-cells from Drd5 knockout mice showed that D5-specific signaling enhanced the suppressive activity of murine T_regs_ in vivo and increased the expression of glucocorticoid-induced tumor necrosis factor receptor-related protein (GITR) (Osorio-Barrios et al., 2018). Thus, broad stimulation of D1-like receptors reduced the suppressive activity of T_regs_, but specific signaling through D5 enhanced this function.

Further, while adoptive transfer experiments showed that D5 signaling drives T_reg_ suppressive functions, in naïve CD4^+^ T-cells, D5 signaling increased proliferation and increased the differentiation of the T_h_17 inflammatory phenotype in the early stages of EAE (Osorio-Barrios et al., 2018). In contrast, when T-cells derived from individuals with lung carcinoma and those undergoing stress were treated with dopamine, it significantly inhibited the proliferation of human CD4^+^ and CD8^+^ T-cells and significantly reduced CD8^+^ T-cell cytotoxicity through D1-mediated cAMP signaling (Saha, Mondal, Basu et al., 2001, Saha, Mondal, Majumder et al., 2001). D1-like receptor activation with SKF-38393 also decreased the suppressive activity of human CD8^+^ T_regs_ (Nasi et al., 2019).

These differences in dopamine signaling may also regulate T-cell differentiation, and D3 and D5 seem to play particularly important roles. In naïve CD4^+^ T-cells, D3 signaling shifts the inflammatory balance toward the T_h_1 and T_h_17 phenotypes, and antigen recognition by naïve CD4^+^ T-cells induces D3 expression (Contreras et al., 2016). D3 stimulation in human T-cells also promotes integrin activation and the expression of IFN-*γ* and TNF-*α* (Levite et al., 2001; Ilani et al., 2004; Besser et al., 2005), indicating that D3 mediates increases in the number of T_h_1 cells. This finding was confirmed in two studies using murine CD4^+^ T-cells, which suggested that D3 mediates the increase in T_h_1-differentiation by reducing ERK phosphorylation, while a D3-mediated reduction in cAMP levels also increased CD4^+^ T-cell activity (González et al., 2013; Franz et al., 2015). One of these studies also showed that D5 signaling mediated increases in ERK phosphorylation and T-cell activity (Franz et al., 2015). Although this effect was not specific to D5, stimulation of D1-like receptors on human lymphocytes increased cAMP production and enhanced T-cell proliferation more than D2 receptor stimulation (Cosentino et al., 2018). However, D3 and D5 were not the only receptors affecting T-cell differentiation, as ablation of D2 receptors in a murine MPTP model of PD also increased T_h_1 and T_h_17 polarization of CD4^+^ T-cells in vivo. Treatment of isolated splenic CD4+ T-cells in vitro with sumarinole (D2 agonist), also blocked T_h_1 and T_h_17 polarization, and this effect was blocked by D2 antagonism with L-741,626 (Liu, Zhai et al., 2021). This differs from what is seen with D3 signaling, suggesting distinct roles among dopamine receptor subtypes. Finally, in naïve murine CD4^+^ T-cells, stimulation of D4 induced IL-2-STAT5 signaling, which promoted a T_h_2 phenotype. This effect was blocked by specific D4-inhibition with L-745,870 and confirmed in Drd4^−/−^ mice, which showed profound age-dependent reduction in T_h_2 inflammation with altered allergen exposure response in the lung of neonatal animals (Wang, Cohen et al., 2019).

These data suggest that all types of dopamine receptors can activate T-cells, particularly through D3 and D5, but dopamine receptor activation more often inhibits activated immune cells. Dopamine alone activates naïve human T-cells, and CD8^+^ T-cells seem to be more responsive to dopamine-mediated inhibition than CD4^+^ T-cells. Dopaminergic regulation of T-cells is important in many disease processes such as skin inflammation or psoriasis (Keren et al., 2019) and PD (González et al., 2013; Ambrosi et al., 2017). We will expand on how the mechanisms discussed in this section can be applied to disease pathophysiology later in this review.

##### b. B-Lymphocytes

There is historically far less research on the role of dopamine in B-cells than on almost any other immune cell type, but recent publications in this area demonstrate a role for dopamine in B-cell immune function. These cells express TH and can store and produce dopamine. Additionally, in activated B-cells, increased production of endogenous catecholamines, including dopamine, norepinephrine and epinephrine, is associated with autocrine activation of *β*-adrenergic receptors and increased IL-10 production (Ferrari et al., 2004; Honke et al., 2022). This is further supported by the finding that T_f_h, upon interaction with B-cells, release stored dopamine to facilitate T_f_h–B-cell interactions and accelerate germinal center output (Papa et al., 2017). B-cells also express dopamine receptors on their surface (Santambrogio et al., 1993; McKenna et al., 2002; Meredith et al., 2006; Wei et al., 2016), indicating that these cells can also respond to dopamine. Treatment with dopamine (10^−9^M–10^−6^M), SKF-38393 (D1-agonist), and LY171555 (D2-agonist) all increased LPS-induced murine splenocyte proliferation (Tsao et al., 1997), suggesting that dopamine receptor signaling could drive B-cell proliferation. However, in B-cell neoplasia, dopamine toxicity may target cycling B-cells independent of their receptors to induce cell cycle arrest and apoptosis (Meredith et al., 2006). This suggests that dopamine regulates B-cell activity via both receptor dependent and independent mechanisms.

The D2-like receptors, particularly D3, may have an important role in B-cell immunity. In a rodent model of EAE requiring effective antigen presentation, the number of D3^+^/CD20^+^ B-cells (antigen-presenting B-cells) was increased in the CNS due to increases CNS tropism mediated by upregulation of CXC chemokine receptor 3 (CXCR3). Further, this subset of cells seems important to the onset of EAE, because knocking out Drd3 in B-cells completely abrogated disease symptoms. Expression of CXCR3 is increased in the CNS of patients with MS, and natalizumab, which blocks lymphocyte entry into the CNS, reduces CXCR3^+^ B-cells in MS brains. However, in a separate EAE model that does not require antigen-presentation, Drd3 knockout in B-cells exacerbated disease progression and increased the number of inflammatory CD4^+^ T-cells in the CNS. This suggested that D3 activation had discrete effects on antigen presenting versus nonantigen-presenting, anti-inflammatory B-cell subsets, demonstrating the importance of dopamine in regulating the function of several types of B-cells (Prado et al., 2021). In another study, expression of D2 on peripheral B-cells was negatively correlated with plasma TNF-*α* levels in patients with RA, suggesting dopamine receptors could influence disease progression by modulating inflammation (Wei et al., 2016). These data indicate an important role for dopamine receptors in the functional regulation of distinct B-cell subsets. Further studies are necessary to better understand the role of other dopamine receptors on B-cells and to determine how these functions influence the behavior of B-cell subsets.

### D. Immunomodulatory Effects of Dopamine

#### 1. Inflammatory Regulation of Dopaminergic Machinery

As dopamine receptors, transporters, and metabolic machinery are expressed in nearly all immune cells, it is not surprising that there is significant crosstalk between the dopaminergic system and the immune function of these cells. While dopamine can modulate the responses of immune cells to inflammatory insults, inflammatory factors released from different immune cells can also impact dopaminergic machinery, altering the function and expression of dopamine receptors and impacting dopamine metabolism. In the CNS, cytokines and chemokines influence the development, maintenance, and functional properties of midbrain dopaminergic neurons (Zalcman et al., 1994; Ling et al., 1998; Dunn, 2006; Felger and Miller, 2012). Inflammatory cytokines such as IL-1*β* promote the differentiation of mesencephalic progenitor cells into dopaminergic neurons (Ling et al., 1998), and IL-6 modulates dopaminergic activity in various mesolimbic structures in a dose-dependent manner (Zalcman et al., 1994; Dunn, 2006).

Inflammation can also regulate dopamine synthesis and release. A number of studies show NO induced changes in dopamine regulation in different brain regions (Lorrain and Hull, 1993; Seilicovich et al., 1995; Hartung et al., 2011; Motahari et al., 2016). Acute exposure to IL-1*β* and IL-6 can stimulate TH expression and dopaminergic activity in vivo and in vitro (Abreu et al., 1994; Zalcman et al., 1994; Ling et al., 1998). In rice stem borer (*Chilo suppressalis*) hemocytes, exposure to LPS stimulates dopamine synthesis (Wu et al., 2015). Inflammatory cytokines also impair the conversion of phenylalanine to tyrosine (Felger et al., 2013), potentially limiting tyrosine availability for dopamine synthesis. In addition to altering the biosynthesis of dopamine, some cytokines can regulate dopamine storage. IL-1*β* and TNF-*α* can decrease the expression of VMAT2, thereby limiting the availability of presynaptic dopamine. Conversely, TGF-*α* increases VMAT2 expression, favoring the storage of presynaptic dopamine (Kazumori et al., 2004). In hMDMs, DAT is highly responsive to inflammatory stimuli, as LPS treatment reduces DAT-mediated uptake and increases DAT-mediated dopamine efflux by promoting an efflux-favoring conformation in these cells. These activities were shown to be part of a TLR4-dependent autocrine loop by which DAT dynamically regulates the amount of dopamine in the local microenvironment to modulate immune functions such as cytokine release and phagocytosis (Mackie et al., 2022).

These results indicate that inflammatory cytokines can exert complex regulatory effects on the amount of dopamine available in dopaminergic cells by modifying the biosynthesis rate and the storage of this neurotransmitter. Finally, it is possible that oxidative stress dysregulates dopamine receptors. In rat renal proximal tubes, oxidative stress caused hyperphosphorylation of DR1, leading to uncoupling of the receptor and G*_α_*_s_ via NF-*κ*B (Banday et al., 2007; Fardoun et al., 2007). This effect was also induced by the TLR4 agonist LPS and may be one mechanism by which dysregulated dopamine receptor signaling can lead to hypertension (Wang, Luo et al., 2014; Yang, Villar et al., 2021). Together, these data support a bidirectional interaction between the immune system and dopamine metabolism, uptake, storage, and receptor signaling.

#### 2. Dopamine-Mediated Impacts on the Inflammatory Response

Multiple studies have demonstrated that dopamine can impact the production of inflammatory mediators in various cell systems, although the precise receptors and signaling pathways involved in this process are complex and not well understood (Tarazona et al., 1995; Hasko et al., 1996, 2002; Capellino et al., 2010; Nakano et al., 2011; Gaskill et al., 2012; Zhang et al., 2015, Zhang, Jiang 2016; Arreola et al., 2016; Nolan and Gaskill, 2019; Yoshioka et al., 2020). These effects are broadly summarized in Table 1. As previously discussed, innate immune cells recognize PAMPs and DAMPs via PRRs. Dopamine often acts on pathways downstream of several of these PRRs, so a baseline understanding of their signaling processes is important for understanding dopamine signaling in the immune response. TLRs coordinate innate immune functions and initiate adaptive immune responses through activation of the NF-*κ*B/activator protein 1 and/or IRF3/7 pathways. Similarly, NOD-like receptor and C-type lectin receptor (dectin-1) signaling activates NF-*κ*B and inflammasomes during bacterial and antifungal infections, respectively. Antiviral immunity is most often mediated by the upregulation of type I interferons via cytoplasmic RIG-I/MDA5, which is a cytoplasmic caspase-recruiting domain helicase (Lee and Kim, 2007). Several of these signaling pathways converge on NF-*κ*B and its downstream effectors. Production of many cytokines is regulated by NF-*κ*B transcriptional activity (Liu et al., 2017), and NF-*κ*B activation is the first step in inflammasome activation and the secretion of IL-1 cytokines in a variety of cell types (He et al., 2016; Herman and Pasinetti, 2018). Signaling pathways involve numerous secondary messengers, kinases, and other signaling effectors (Kawai and Akira, 2009; Takeuchi and Akira, 2010), many of which can be activated by distinct dopamine signaling pathways (Beaulieu and Gainetdinov, 2011; Beaulieu et al., 2015), demonstrating substantial crosstalk between PRRs and dopamine signaling.

**TABLE 1:** Dopamine-mediated immune function

Species	Cell type/region	Receptors or proteins involved	Dopamine/dopamine modifying agent or method	Immunologic finding	Method(s) of Detection	Reference
Chicken	Macrophage	N/A	Dopamine (0.1–0.25 *μ*g/mL)	↑ Phagocytosis of *E. coli* and sheep red blood cells ↑ Percentage of Fc-receptor positive macrophages	Phagocytosis assay Fc-receptor Assay	Ali et al., 1994
Guinea Pig	Macrophage (spleen)	D1-like D2-like	Bromocriptine (0.005–0.5 *μ*g/mg) Leuprolide (0.005–0.5 *μ*g/mg) Pergolide (0.001–0.25 *μ*g/mg) Chlorpromazine (0.5–50 mg/kg) Metochlopramide (0.5–50 mg/kg) Sulpiride (1–50 mg/kg) Veralipride (1–50 mg/kg) Alizapride (1–50 mg/kg) Cisapride (0.1–5 mg/kg)	↑ Clearance of IgG-sensitized RBCs, in vitro binding of IgG-sensitized RBCs, and cell surface expression of Fc*γ* by dopamine agonists (opposite effect with dopamine antagonists)	Fc-receptor assay Flow cytometry	Gomez et al., 1999
Human	B-cell (BC-3 cell line)	D1-like D2-like	N-acyl-dopamine derivatives (5–10 *μ*M) SCH23390 (10 *μ*M) Haloperidol (10 *μ*M)	N-acyl-dopamine derivatives reactivated Kaposi's sarcoma-associated herpesvirus (blocked by SCH23390 and haloperidol)	Luciferase reporter assay	Lee et al., 2008
Mouse	B-cell	D3	N/A	↑ CNS-tropism of pro-inflammatory B-cells with antigen presenting function ↑ CNS homing of anti-inflammatory B-cells without antigen presenting function	Flow cytometry	Prado et al., 2021
Human	Brain (striatum, caudate, putamen)	D2	Methylphenidate (40 mg)	↑ Methylphenidate-induced D2 activation after LPS administration compared with placebo	^11^C-raclopride PET	Petrulli et al., 2017
Human	T-cell (CD4^+^CD45RA^+^)	N/A	Dopamine (1 nM–1 *μ*M)	↑ CD3/CD28-stimulated IL-4 and IL-5 in T-cells treated with dopamine	ELISA	Nakano et al., 2009
Human	Dendritic cell (monocyte-derived	D1-like D2-like	Sulpiride (100 nM) Nemonapride (3 nM) SCH-23390 (1 *μ*M)	↑ IL-5:IFN*γ* ratio and CCR4 with sulpiride and nemonapride ↓ CXCR3 in co-culture with T-cells ↓ IL-5:IFN*γ* ratio with SCH-23390	ELISA	Nakano et al., 2009
Human	Keratinocyte (HaCa T-cell line)	D2-like DR-independent (*β*-adrenergic) Dopamine-dependent oxidative stress	Dopamine (100 nM–100 *μ*M) Cabergoline (10–100 *μ*M) Sulpiride at (10–100 *μ*M) Ascorbic acid (0.1 mM)	↑ IL-6 with dopamine (effect reduced by ascorbic acid and sulpiride) ↑ IL-8 with dopamine ↑ IL-6 with cabergoline (effect blocked by sulpiride)	ELISA	Parrado et al., 2012
Human	T-cell (PBL, CD4^+^, and CD8^+^)	D3	Quinpirole (100 nM–10 *μ*M) U-maleate (100 nM–10 *μ*M)	↑ PHA + IL-2-activated IFN-*γ* and ↓ IL-4 and IL-10 in total and CD4^+^ T-cells by quinpirole (reversed by U-maleate in total T-cells) ↑ PHA + IL-2-activated IFN-*γ* in CD8^+^ T-cells by quinpirole	RT-PCR	Ilani et al., 2004
Human	Lymphocyte (peripheral blood)	D1-like Dopamine-dependent oxidative stress	Dopamine (100 nM–500 *μ*M) N-acetyl-L-cysteine (5 mM) SCH 23390 (1 *μ*M)	↓ ROS and apoptosis by low-dose dopamine (blocked by SCH 23390) ↑ ROS and apoptosis by high-dose dopamine (blocked by N-acetyl-L-cysteine)	Flow cytometry Fluorescent assay	Cosentino et al., 2004
Human	Lymphocyte (peripheral blood)	N/A	L-DOPA (10–100 *μ*M) Dopamine (10–100 *μ*M)	↓ Con-A and PWM-stimulated proliferation by L-DOPA ↓ Con-A-stimulated proliferation and IFN*γ* by dopamine	3H-thymidine incorporation assay	Bergquist et al., 1994
Human	Macrophage (monocyte-derived)	N/A	Dopamine (20 nM–20 *μ*M)	↑ IL-6 and CCL2 ↑ IL-6, CCL2, IL-8 and IL-10 in LPS-treated cells ↓ TNF-a in LPS-treated cells	ELISA	Gaskill et al., 2012
Human	Macrophage (monocyte-derived)	N/A	Dopamine (1 *μ*M)	↑ NLRP3 and IL-1*β*, NF-*κ*B nuclear translocation, and ATP-induced IL-1*β* release ↑ Impact of cytomegalovirus on NF-*κ*B activation	Alphalisa Immunofluorescence Western Blot qPCR	Nolan et al., 2020
Human	Macrophage (monocyte-derived) Microglia (C06 cell line)	D1-like	Dopamine (1 *μ*M)	↑ CCR5 in both macrophages and microglia D1-like receptors correlate with CCR5 expression in macrophages	Flow cytometry qPCR Western blot	Matt et al., 2021
Human	Macrophage (monocyte-derived)	D1-like	Dopamine (1 nM–1 *μ*M)	↑ IL-1*β*, IL-6, IL-18, CCL2, CXCL8, CXCL9, and CXCL10	Alphalisa	Nolan et al., 2019
Human	Macrophage (monocyte-derived)	DAT	Nomifensine (10 *μ*M)	↑ IL-6 and TNF-*α*	Flow cytometry qPCR Immunocytochemistry	Mackie et al., 2022
Human	Microglia (primary)	N/A	Dopamine (2.5 *μ*M)	↑ Extracellular traps	Immunofluorescence	Agrawal et al., 2021
Human	T-cell (CD4^+^) coculture with dendritic cells (monocyte-derived);	D1-like D2-like	SCH23390 (1 *μ*M) L750667 (1 *μ*M)	↓ IL-17 and ↑ IFN*γ* with SCH23390 ↑ IL-17 and ↓ IFN*γ* with L750667	ELISA	Nakano et al., 2008
Human	Monocyte (peripheral blood and THP-1 cell line)	N/A	Dopamine (10 nM–2 mM)	↓ LPS-stimulated proliferation with high dose dopamine ↓ LPS-induced NF-*κ*B binding to the TNF*α* promoter	3H-thymidine incorporation assay EMSA	Bergquist et al., 2000
Human	Monocyte (THP-1 cell line)	D1 D3 D4	Dopamine (1 *μ*M) SKF 38393 (1–10 *μ*M) SCH 23390 (100 nM–1 *μ*M) Promipexole (1–10 *μ*M) Pnu 177864 (1–10 *μ*M) PD 168077 (100 nM–1 *μ*M) L 745.870 (100 nM–1 *μ*M)	↑ NF-*κ*B nuclear translocation with dopamine ↑ CCR5 promoter RNA pol II, CCR5 transcription, CCR5 promoter H3K4me3 and H3K27Ac modifications, and CCR5 protein with dopamine ↓ CCR5 with SKF 38393, Pnu 177864, and L 745.870 ↑ CCR5 with SCH 23390, promipexole and PD 168077	Immunofluorescence ChIP qPCR Flow cytometry	Basova et al., 2018
Human	Neutrophil	D1-independent mechanism	Dopamine (10–100 *μ*M) Fenoldopam (1 nM–100 *μ*M)	↑ Apoptosis and phagocytosis by dopamine (not changed by fenoldopam)	Flow cytometry	Sookhai et al., 1999
Human	NK cell (CD56^+^)	D1-like	Dopamine (1aM–1 nM) SCH23390 (1nM–1 *μ*M) SKF38393 (100 nM)	↓ Proliferation with SKF38393 and dopamine ↓ IFN*γ* with dopamine, SKF38393, and SCH23390	Proliferation assay ELISA	Mikulak et al., 2014
Human	Osteoclast (monocyte-derived)	D2-like	Dopamine (100 pM–1 *μ*M) Pramipexole (5–500 pg/ml) Quinpirole (1 nM–100 nM) Haloperidol (1–10 nM)	↓ TRAP-positive cells with dopamine (mimicked by pramipexole and quinpirole and blocked by haloperidol) ↓ Osteoclast-mediated bone resorption with dopamine and D2-like agonists	TRAP assay Pit formation assay	Hanami et al., 2013
Human	Peripheral blood mononuclear cells	N/A	L-DOPA (10–500 *μ*M) Dopamine (10 nM–500 *μ*M)	↓ Con A and PWM-stimulated proliferation, IFN*γ* and IL-4 with L-DOPA and dopamine ↑ Apoptosis with L-DOPA and dopamine ↓ IgM and IgG-producing B-cells with dopamine	3H-thymidine incorporation assay ELISA	Bergquist et al., 1997
Human	T-cell	D1-like D2 D3	Dopamine (10 nM–100 *μ*M) U-maleate (1 *μ*M) Quinpirole (10–100 nM) 7-OH-DPAT (10–100 nM) SKF 38393 (10–100 nM) L-741,626 (100 nM)	↑ TNF-*α* expression and secretion with dopamine (blocked by U- maleate) ↑ TNF-*α* with SKF 38393 and 7-OH-DPAT ↑ IL-10 expression and secretion by dopamine (blocked by L-741,626) ↑ IL-10 with SKF 38393 and quinpirole	RT-PCR ELISA	Besser et al., 2005
Human	T-cell	D2 D3	Dopamine (1–5 ng/mL) Eticlopride (not specified) U99194A (not specified)	↓ CD3-activated proliferation, IL-2, IFN*γ* and IL-4 (blocked by eticlopride and U99194A)	3H-thymidine incorporation assay ELISA	Ghosh et al., 2003
Human	T-cell	D4	PD 168, 077 (1 *μ*M) ABT 724 trihydrochloride (1 *μ*M)	↓ CD3/CD28-activated proliferation, IL-2 and iERK1/ERK2 phosphorylation with PD 168, 077 and ABT 724 trihydrochloride	3H-thymidine incorporation assay ELISA	Sarkar et al., 2006
Human	T-cell [CD4^+^, CD8^+^, and lymphokine activated killer T-cells (LAK-T)]	D1-like	Dopamine (10.2–48.6 pg/mL) SCH23390 (10 ng/mL)	↓ IL-2-induced proliferation of CD3-activated CD4^+^ and CD8^+^ T-cells with high dose dopamine ↓ Cytotoxic ability of LAK-T-cells with dopamine (blocked by SCH23390)	3H-thymidine incorporation assay Cytotoxicity assay (^51^Cr release)	Saha et al., 2001
Human	T cell (CD4^+^ T_reg_)	D1-like D2-like	SKF38393 (10 pM) Pramipexole (50 pM)	↑ Intracellular cAMP with SKF38393 ↓ Intracellular cAMP with pramipexole ↓ Proliferation with increasing D1-like activity based on genetic polymorphism	ELISA	Cosentino et al., 2018
Human	T cell (CD4^+^CD25^+^ T_regs_)	D1-like	Reserpine (1 *μ*M) SCH23390 (1 *μ*M)	↓ IL-10 and TGF-*β* with reserpine (blocked by SCH23390)	RT-PCR ELISA	Cosentino et al., 2007
Human	T-cell (CD8^+^)	D3	Dopamine (10 nM–1 *μ*M) 7-OH-DPAT (100 nM) U-99194A (100 nM)	↑ Adhesion to fibronectin and ICAM-1 with dopamine and 7-OH-DPAT ↑ Chemotactic migration with dopamine ↑ CCL19-, CCL21-, and CXCL12 -induced chemotaxis with dopamine in naive CD8^+^ T-cells	Adhesion assay Chemotaxis assay Migration assay	Watanabe et al., 2006
Human	T-cell (CD8^+^)	N/A	Dopamine (1 *μ*M)	↑ Adhesion of naïve CD8^+^ T-cells ↑ Migration of naïve but not CD3/CD28-activated CD8^+^ T-cells ↓ CD3/CD28 cross-linking, IL-2, Erk1/2 and NF-*κ*B	Flow-through adhesion assay Migration assay ELISA	Strell et al., 2009
Human	T cell (CD8^+^ T_reg_)	D1-like	Dopamine (10 nM) SKF-38393 (10 nM)	↓ T-cell-mediated proliferation of human PBMCs with dopamine and SKF-38393	Flow cytometry	Nasi et al., 2019
Human	T-cell (naïve)	D2 D3	Dopamine (10 nM) 7-OH-DPAT (10 nM) U-Maleate (10 nM) Bromocriptine (10 nM) Pergolide (10 nM) Butaclamol (10 nM–1 *μ*M) Haloperidol (10 nM–1 *μ*M)	↑ Adhesion to fibronectin with dopamine and DPAT (blocked by U-Maleate) ↑ Adhesion to fibronectin with bromocriptine and pergolide ↓ Adhesion to fibronectin with butaclamol and haloperidol	T cell adhesion assay	Levite et al., 2001
Mouse	T-cell (CD4^+^CD25^+^ T_reg_, CD4^+^CD25^-^ T effectors from spleen or lymph node)	D1-like	Dopamine (100 nM–10 *μ*M) SKF-38393 (10 *μ*M) SCH-23390 (10 *μ*M)	↓ T effector proliferation in co-culture with T_reg_ with dopamine (mimicked by SKF-38393 and blocked by SCH-23390) ↓ CD3/CD28-activated IL-10 in T_regs_ with dopamine ↓ T_reg_ adherence to CSPG with dopamine (mimicked by SKF-38393 and blocked by SCH-23390)	3H-thymidine incorporation assay ELISA Adhesion assay	Kipnis et al., 2004
Mouse	T-cell (CD 4+ T-cell)	D4	Dopamine (0.1–25 *μ*M) A412997 (100 nM) L745870 (300 nM)	↑ Th2 differentiation ↑ *Il13* gene expression	Flow cytometry qPCR	Wang, Cohen et al., 2019
Mouse	Dendritic cell (bone-marrow derived, D5 KO)	D5	SKF38393 (1 nM) SCH23390 (1 nM)	↓ ERK1/2 phosphorylation in LPS-stimulated cells with SKF38393 ↓ IL-12 with intracellular dopamine depletion ↓ IL-23 in D5 KO	Western blot ELISA	Prado et al., 2012
Mouse	Dendritic cell (bone-marrow derived)	D2	Haloperidol (5 *μ*g/mL) SCH23390 (3–5 *μ*g/mL) L750667 (3–5 *μ*g/mL)	↓ CD80, CD86, MHC ΙІ, CD83 LPS-induced IL-12p40 with haloperidol ↓ T-cell proliferation and IFN*γ* in co-culture with haloperidol-treated dendritic cells ↓ Dendritic cell maturation with L750667	Flow cytometry ELISA	Matsumoto et al., 2015
Mouse	Macrophage (bone marrow–derived and RAW264.7 cell line)	D2	Haloperidol (10 *μ*M) L750.667 (10 *μ*M)	↓ LPS-stimulated CD80 in RAW cells and ↓ LPS-stimulated CD80 and CD86 in BMDM with haloperidol ↓ LPS-stimulated IL-1*β*, IL-6, IL12p40 and NF-*κ*B activity in RAW cells with haloperidol ↓ LPS-stimulated CD80 and IL-6 in RAW cells with L750.667	Flow cytometry ELISA Reporter assay	Yamamoto et al., 2016
Mouse	Macrophage (bone-marrow derived)	D5	Dopamine (500 nM–400 *μ*M)	↓ Pam3 or S. aureus- induced expression of IL-6 and TNF-*α* (blocked by D5 knockdown) ↓ S. aureus-induced IL-12 ↓ Pam3 or S. aureus-induced phosphorylation of IKK and IkBa	ELISA qPCR	Wu et al., 2020
Mouse	Macrophage (bone-marrow derived)	D1	Dopamine (150–250 *μ*M) A-68930 (20–30 *μ*M)	↓ LPS-primed nigericin stimulated IL-1*β*, IL-18, and caspase-1 with dopamine (blocked by D1 knockdown) A-68930 inhibited nigericin-induced IL-1*β* secretion	ELISA Western blot	Yan et al., 2015
Mouse	Macrophage (peritoneum and RAW264.7 cell line)	D2	Dopamine (40–1040 *μ*M) Eticlopride (50 *μ*M)	↑ TNF-*α* and IL-12 and ↓ TGF-*β*, IL-10 and VEGF (blocked by eticlopride or D2 small interfering RNA)	ELISA	Qin et al., 2015
Mouse	Macrophage (peritoneum and J774.1 cell line)	DR-independent (*β*-adrenergic)	Dopamine (100 nM–100 *μ*M) Propranolol (1–10 *μ*M)	↓ IL-12 p40 expression and release from LPS-stimulated J774.1 cells with dopamine (blocked by propranolol) ↑ IL-10 in both cell types with dopamine (only blocked by propranolol in J774.1 cells)	RT-PCR ELISA	Hasko et al., 2002
Mouse	Macrophage (peritoneum)	D1	Fenoldopam (1 *μ*M)	↓ LPS-stimulated TNF-*α* and MIP-2 with fenoldopam	ELISA	Bone et al., 2017
Mouse	Macrophage (peritoneum)	D1-like D2-like	Bromocryptine (8 mg/kg) Quinpirole (3 mg/kg) Sulpiride (100 mg/kg) SCH-23390 (0.5 mg/kg)	↓ LPS-stimulated TNF-*α* in plasma and NO in peritoneal macrophages with bromocriptine, quinpirole and sulpiride ↓ LPS-stimulated NO in peritoneal macrophages with SCH-23390	ELISA Griess assay	Hasko et al., 1996
Mouse	Macrophage (RAW264.7 cell line)	DR-independent (*β*- adrenergic)	Dopamine (500 nM–50 *μ*M)	↑ LPS-induced NO at high concentrations ↓ NO at low concentrations	Griess assay Western blot	Chi et al., 2003
Mouse	Macrophage (RAW264.7 cell line)	N/A	Dopamine (10 *μ*M–1 mM)	↓ TNF-*α*, IL-6, IL-1*β*, iNOS, NLRP3, and caspase-1 mRNA and protein	Western blot qPCR	Liu et al., 2019
Mouse	Mast cell (bone marrow–derived)	D1-like	Dopamine (100 pM–100 nM) SCH 23390 (100–1000 nM)	↑ Mast cell degranulation with dopamine (blocked by SCH 23390)	*β*-Hexosaminidase assay	Mori et al., 2013
Mouse	Microglia (BV-2 cell line)	D1-like D2-like	Isosibiricin (50 *μ*M) SCH 23390 (1 *μ*M) Sultopride (1 *μ*M)	↓ IL-1*β*, IL-18, NLRP3, caspase-1 with isosibiricin (blocked by SCH 23390 and sultopride)	ELISA Western blot	Wang, Yv et al., 2019
Mouse	Microglia (BV-2 cell line)	N/A	Dopamine (250 *μ*M)	↑ Extracellular traps	Immunofluorescence	Agrawal et al., 2021
Mouse	Microglia (N9 cell line)	DR-independent (*α* and *β*− adrenergic)	Dopamine (1 *μ*M) L-DOPA (1–250 *μ*M)	↓ LPS-stimulated NO with dopamine ↓ LPS-stimulated NO at high concentrations of L-DOPA ↓ iNOS with dopamine	Griess assay Western blot	Chang and Liu 2000
Mouse	Microglia (BV-2 cell line)	Dopamine-dependent oxidative stress	Dopamine (–100 *μ*M) N-acetyl-L-cysteine (10 mM)	↓ LPS-induced NO with dopamine (blocked by N-acetyl-L-cysteine)	Griess assay Western blot	Yoshioka et al., 2016
Mouse	Microglia (primary)	Dopamine-dependent oxidative stress	Dopamine (1–100 *μ*M) Sulpiride (10–30 *μ*M) Bromocriptine (1–10 *μ*M) N-acetyl-L-cysteine (10 mM)	↓ LPS-induced NO with dopamine (partially inhibited by sulpiride and blocked by N-acetyl-L-cysteine) ↓ LPS-induced NO with bromocriptine	Griess assay Western blot	Yoshioka et al., 2016
Mouse	Microglia (primary and BV-2 cell line)	DAT	Dopamine (2 *μ*M) Benztropine (2 *μ*M) Vanoxerine (2 *μ*M)	↓ Number of cellular processes, ↑ cell adhesion/spreading, and ↑ vimentin filaments with dopamine ↓ LPS-induced cell spreading and phagocytosis with dopamine ↓ ERK1/2 phosphorylation in activated but not resting cells with dopamine ↑ p38MAPK activity in resting but not activated cells (attenuated by benztropine and vanoxerine)	Immunofluorescence Phagocytosis assay Western blot	Fan et al., 2018
Mouse	Microglia (primary)	D2 D3	Pramipexole (100 nM–100 *μ*M)	↑ LPS and IFN-*γ*-stimulated nitrite release	Griess assay	Huck et al., 2015
Mouse	Microglia (primary)	D1-like D2-like	Dopamine (1–10 *μ*M) Dihydrexidine (10 *μ*M) Quinpirole (10 *μ*M) SCH23390 (10 *μ*M) Sulpiride (10 *μ*M)	↓ LPS-induced NO with dopamine, dihydrexidine, and quinpirole (blocked by SCH23390 and sulpiride) ↑ Migration with dopamine, dihydrexidine, and quinpirole	ELISA Griess assay Filter assay	Farber et al., 2005
Mouse	NK cell (spleen)	D1-like D2-like	SKF38393 (10–100 nM) Quinpirole (10–100 nM) SCH23390 (10 nM–1 *μ*M) Haloperidol (10 nM–1 *μ*M)	↑ Cytotoxicity of NK cells against YAC-1 lymphoma cells with SKF38393 (blocked by SCH23390) ↓ Cytotoxicity of NK cells against YAC-1 lymphoma cells with quinpirole (blocked by haloperidol)	Western blot	Zhao et al., 2013
Mouse	Osteoclast (bone marrow–derived)	D2-like	Pramipexole (3mg/kg)	↓ Ex vivo osteoclastogenesis in in vivo LPS and pramipexole-treated mice	TRAP assay	Hanami et al., 2013
Mouse	Splenocyte	D1-like D2-like	MPTP (20mg/kg) SKF38393 (1, 5, 10 *μ*g/kg, 1 nM–1 *μ*M in vitro) LY171555 (1, 5, 10 *μ*g/kg, 1 nM–1 *μ*M in vitro) Dopamine (1 nM–1 *μ*M)	↑ LPS or Con A- stimulated proliferation with SKF38393 or LY171555 in vitro and in vivo ↓ LPS or Con A- stimulated proliferation with MPTP in vivo	Proliferation assay	Tsao et al., 1997
Mouse	Splenocyte	N/A	L-DOPA (10–500 *μ*M) Dopamine (10–500 *μ*M)	↓ ConA and LPS-stimulated proliferation with L-DOPA and dopamine ↓ IL-2, IL-6 and IFN*γ* with L-DOPA and dopamine	3H-thymidine Iincorporation assay Flow cytometry ELISA	Josefsson et al., 1996
Mouse	Splenocyte	D2-like	L-DOPA (126 mg/kg) Dopamine (5 *μ*g/kg) Domperidone (5 mg/kg)	↓ Number of IFN*γ*-producing cells with dopamine ↑ In vitro Con-A-stimulated proliferation and ↓ number of IFN*γ*-producing cells with L-DOPA in vivo (blocked by domperidone)	3H-thymidine incorporation assay ELISpot	Carr et al., 2003
Mouse	Splenocyte (NK cells and T-cells)	DAT	N/A	↓ Splenic NK activity in DAT deficient cells ↓ IFN-*γ*, IL-10, and proliferation in mitogenic-stimulated DAT deficient T-cells	ELISA	Kavelaars et al., 2005
Mouse	Macrophage (peritoneum)	DAT	N/A	↑ LPS-stimulated TNF-*α* and IL-10 in DAT deficient cells	ELISA	Kavelaars et al., 2005
Mouse	Splenocyte Thymocyte	N/A	Dopamine (10–100 *μ*M)	↓ Con-A or LPS-induced splenocyte and thymocyte activation with dopamine	3H-thymidine incorporation assay	Cook-Mills et al., 1995
Mouse	Splenocyte Thymocyte	D1-like D2-like	Haloperidol (10 nM–1 *μ*M) Chlorpromazine (10 nM–1 *μ*M) Flupentixol (10 nM–1 *μ*M)	↓ Con A-induced splenocyte and thymocyte proliferation with chlorpromazine, haloperidol, and flupentixol ↓ IL-2 with haloperidol, chlorpromazine, flupentixol	3H-thymidine incorporation assay	Boukhris et al., 1988
Mouse	T-cell (CD4^+^, D3 and D5 KO)	D3 D5	PD128907 (5–50 nM) SKF38393 (20 nM)	↓ IL-2 in CD3/CD28 stimulated D3 and D5 deficient cells ↓ IL-2 in CD3/CD28 stimulated cells with PD128907 and SKF38393	ELISA	Franz et al., 2015
Mouse	T-cell (CD8^+^)	D3	Dopamine (10nM–1 *μ*M or 0.1 nmol in 200 *μ*L in vivo) 7-OH-DPAT (100 nM or 0.1nmol in 200 *μ*L in vivo) U-99194A (100 nM or 20 mg/kg in vivo)	↑ Chemotactic migration with dopamine ↑ Naive T-cell migration to peritoneum with dopamine or 7-OH-DPAT ↓ Naive T-cell homing into lymph nodes with U-99194A	Chemotaxis assay Migration assay Flow cytometry	Watanabe et al., 2006
Mouse	T-cell (mesenteric lymph node)	D1-like D2-like	SKF38393 (1 nM–10 *μ*M) SCH23390 (100 nM) Quinpirole (1 nM–10 *μ*M) Haloperidol (100 pM–100 nM)	↓ IFN*γ* with SKF38393 (blocked by SCH23390); ↓ IFN*γ* and Con A-stimulated proliferation and ↑ IL-4 with quinpirole (blocked by haloperidol)	MTT assay Cytometric bead array	Huang et al., 2010
Mouse	T-cell (CD4^+^, D3 KO)	D3	N/A	↓ IFN-*γ* and Foxp3 and ↑ Gata3 and IL-4 in Th2-polarized D3 deficient cells	Flow cytometry qPCR	Contreras et al., 2016
Rat	Macrophage (peritoneum)	D2-like	Domperidone (10 nM)	↓ Oxidative burst and PMA-induced burst	Flow cytometry	Carvalho-Freitas et al., 2008
Rat	Macrophage (tumor tissue)	D2	Dopamine (25–50 mg/kg) Eticlopride (7.5 mg/kg)	↑ iNOS, CXCL9, TNF-*α*, IL-12 and ↓ Arginase-1, CD206, TGF-*β* and IL-10 with dopamine (effects blocked by eticlopride)	qPCR Western blot ELISA	Qin et al., 2015
Rat	Mast cell (RBL-2H3 cell line)	D1 D2	GLC756 (100 nM–30 *μ*M)	↓ IgE-activated TNF-*α* with GLC756	ELISA	Laengle et al., 2006
Rat	Microglia (primary)	N/A	Dopamine (10 *μ*M)	↓ LPS-induced NO with dopamine	ELISA Griess assay	Farber et al., 2005
Rat	T-cell	D3	L-DOPA (30 mg/kg) Carbidopa (10 mg/kg)	↑ IFN-*γ*, VLA-4 and CD25 with L-DOPA and carbidopa	RT-PCR Flow cytometry	Ilani et al., 2004

ChIP, chromatin immunoprecipitation; iNOS, NO synthase; N/A, not applicable; PMA, phorbol myristate acetate; qPCR, quantitative polymerase chain reaction; RT-PCR, reverse-transcription polymerase chain reaction; TRAP, tartrate-resistant acid phosphatase; VEGF, vascular endothelial growth factor; VLA-4, very late antigen 4.

The transcription factor NF-*κ*B is central to the initiation and regulation of PRR-mediated inflammatory responses (Liu et al., 2017), and the impact of dopamine on NF-*κ*B has been examined in a variety of immune cell subsets (Takeuchi and Fukunaga, 2003; Zhang et al., 2015; Nolan et al., 2019; Wang, Chen et al., 2019; Wu et al., 2020; Yoshioka et al., 2020; Yue et al., 2021). A number of these studies suggest that dopamine inhibits NF-*κ*B, indicating anti-inflammatory activity. In the macrophage RAW264.7 cell line, very high levels of dopamine (10^−3^–10^−5^ M) reduced both LPS-induced expression of NO synthase and the expression of the NLRP3 inflammasome components NLRP3 and caspase-1 (Liu and Ding, 2019). Although this study did not directly demonstrate that dopamine impacted NF-*κ*B, activation of this transcription factor is necessary for NLRP3 expression, and so the downregulation of NLRP3 indicates reductions in NF-*κ*B activity (He et al., 2016; Herman and Pasinetti, 2018). Rotigotine (D2-like agonist) reduced TNF-*α* production and NF-*κ*B activation in a mouse model of acute liver injury (Yue et al., 2021), and D2 signaling inhibited NF-*κ*B activation by inhibiting Akt in a murine model of acute pancreatitis (Han et al., 2017). These effects are not unique to myeloid cells, as D2 activation in resting peripheral human T-cells was associated with IL-10 secretion, which regulates cytokine production via NF-*κ*B (Besser et al., 2005). In CD3-stimulated human T-cells, D2 and D3 activation inhibited the dopamine-mediated release of IL-2, IFN-*γ*, and IL-4 (Ghosh et al., 2003). In a separate study, D4 blocked IL-2 production in CD3/CD28-stimulated human T-cells (Sarkar et al., 2006), indicating that under certain conditions, the activation of all three D2-like receptors can be anti-inflammatory. Dopamine treatment of astrocytes may also inhibit NF-*κ*B and the NLRP3 inflammasome through D2-like receptor signaling (Shao et al., 2013; Zhu, Hu et al., 2018).

Dopamine signaling through D1-like receptors may also reduce NF-*κ*B activity, potentially via PP2A activation (Wu et al., 2020), which is also linked to Akt inhibition (Manning and Toker, 2017). Dopamine (∼6–18 × 10^−5^M) also decreased inflammatory cytokine production and NLRP3 activity in murine lung tissue in ventilator-induced lung injury models (Yang et al., 2017). Treatment with A68,930 (D1-like agonist) inhibited NLRP3 activation in doxorubicin-treated murine cardiac myoblasts, as well as in mice with cardiac inflammation induced by doxorubicin, CNS inflammation induced by intracerebral hemorrhage, and acute kidney injury caused by renal ischemia/reperfusion (Wang, Nowrangi et al., 2018; Cao et al., 2020; Liu, Jin et al., 2021).

In murine BMDMs treated with the TLR2 ligand Pam3CSK4, D5 activation mediated the downregulation of NLRP3 (Wu et al., 2020), and signaling through D1 decreased NLRP3 activity by increasing NLRP3 ubiquitination in LPS-induced dopamine treated (1.5–2.5 × 10^−4^M) cells (Yan et al., 2015). These studies show that high levels of dopamine or specific D1-like receptor activation in rodents and rodent macrophages can downregulate NLRP3 activity. Several studies also suggest that this effect is mediated by cAMP signaling, although D1 signaling was not shown to act directly on cAMP production (Yan et al., 2015; Wang, Villar et al., 2018; Liu, Jin et al., 2021). The connection may therefore be indirect or circumstantial, as data in human macrophages suggest that D1-like receptors do not activate cAMP in this cell type (Nickoloff-Bybel et al., 2019).

Although these aforementioned studies showed an anti-inflammatory effect of D1-like receptor signaling, they examined the effects of high levels of dopamine or D1-specific agonists on existing inflammation. A number of other studies indicate an inflammatory role for dopamine, which can also be mediated by NF-*κ*B and NLRP3 activity (Nakano et al., 2008, 2011; Gaskill et al., 2012; Parrado et al., 2012; Trudler et al., 2014; Wang, Villar et al., 2018; Nolan et al., 2019, 2020). In hMDMs, dopamine (10^−8^–10^−6^M) increased the production of the inflammatory mediators CCL2, IL-6, C-X-C motif chemokine ligand (CXCL) 8, IL-1*β*, CXCL9, and CXCL10 (Gaskill et al., 2012; Nolan et al., 2019). Similarly, dopamine (2 × 10^−6^M–10^−5^M) increased IL-6 and IL-1*β* production in activated primary murine microglia, NK cells, and BV-2 murine microglial cells (Trudler et al., 2014; Fan et al., 2018; Nolan et al., 2019), and one study showed increased ERK1/2 and p38MAPK activation in LPS-induced rodent primary microglia and BV2 cells relative to resting cells (Fan et al., 2018). IL-1*β* production was also increased in LPS-induced peritoneal macrophages (Kawano et al., 2018). In hMDMs, specifically blocking DAT activity enhanced LPS-mediated production of IL-6, TNF-*α*, and mitochondrial superoxide levels, showing that the regulation of dopamine uptake and release, as well as dopamine receptor activity, plays a role in dopaminergic immunomodulation in macrophages (Mackie et al., 2022).

These effects are not limited to myeloid cells. In naïve human CD4^+^ T-cells stimulated with anti-CD3/anti-CD28, dopamine (10^−8^–10^−7^M) increased the production of IL-5, IL-17, IL-1*β*, and IL-6 and the dopamine-mediated changes in IL-5 and IL-17 were blocked by the presence of SCH23390 (D1-like receptor antagonist) (Nakano et al., 2011). The secretion of TNF-*α* by resting human peripheral T-cells also increased in response to D1/D5 and D3 activation (Besser et al., 2005). Activation of D3 also drives inflammatory effects in several other human and rodent systems, biasing the generation of inflammatory T_h_1 and T_h_17 cells and suppressing the development of T_h_2 cells (González et al., 2013; Ilani et al., 2004; Contreras et al., 2016). In a mouse model of intestinal inflammation, the transplantation of naïve Drd3*^−/−^* T-cells resulted in milder weight loss and mucosal inflammation than the transplantation of wild-type cells (Contreras et al., 2016). In an MPTP model of PD, Drd3 knockout animals showed decreased microglial activation and reductions in TNF-*α* and IFN-*γ* production due to a lack of D3 signaling in T-cells (González et al., 2013). In addition to traditional immune cells, in human adipocytes, pharmacological stimulation of D2 induced the expression and release of IL-6 (Wang, Villar et al., 2018), and in human keratinocytes, D2 activation increased IL-6 and IL-8 levels (Parrado et al., 2012).

Inflammatory responses were also suppressed by SCH23390 in a SCID-mouse model of murine RA, while D2-like receptor suppression with haloperidol increased the accumulation of IL-6^+^ and IL-17^+^ T-cells (Nakano et al., 2011). In a murine model of EAE, SCH23390 (D1-like receptor antagonist) decreased IL-17 production (Nakano et al., 2008), and in a different murine model of EAE, dopamine was found to exacerbate the disease, as removal of dopamine reduced EAE severity. Much of this effect was found to be driven by D5 signaling in DCs, which increased the frequency of inflammatory CD4^+^ T-cells in the CNS and drove inflammation by blocking signal transducer and activator of transcription and inducing the activity of IL-12 and IL-23 in T-cells (Prado et al., 2018). Further, dopamine (10^−6^M–10^−5^M) increased the anti-inflammatory mediator IL-10 in LPS-induced hMDMs (Gaskill et al., 2012; Nolan et al., 2019), LPS-induced primary murine splenocytes, B-cells, and bone marrow–derived DCs (Kawano et al., 2018), although the effect of A77636 (D1-like receptor agonist) on splenocytes was inhibitory (Kawano et al., 2018).

Many studies of innate immune cells such as macrophages indicate that the regulation of NF-*κ*B and the NLRP3 inflammasome is central to the inflammatory impact of dopamine. In hMDMs, dopamine increases the activation of NF-*κ*B, leading to the expression of NLRP3 and IL-1*β*. Dopamine could also potentiate ATP-induced secretion of IL-1*β* but did not mediate the secretion of IL-1*β* on its own, which indicates that dopamine can prime but not activate the NLRP3 inflammasome by activating NF-*κ*B (Nolan et al., 2020). Many previously discussed studies also show that dopamine or specific dopamine receptors increase and decrease NF-*κ*B and NLRP3 inflammasome activity (Nakano et al., 2011; Shao et al., 2013; Trudler et al., 2014; Yan et al., 2015; Yamamoto et al., 2016; Wang, Nowrangi et al., 2018; Wu et al., 2020; Liu, Jin et al., 2021).

These data, along with other, similar research, indicate that NF-*κ*B and the NLRP3 inflammasome may be central to dopaminergic modulation of inflammation, at least in macrophages and other types of innate immune cells. Notably, most studies only examined the NLRP3 inflammasome, and the effects of dopamine on other inflammasomes remain unclear. These data show the broad range of cell types on which dopamine can act and highlight the discrete roles of specific dopamine receptor subtypes, as well as how they could differ between cell types. While many of these data are conflicting, they demonstrate that dopamine acts differently on activated or stimulated cells than on unstimulated cells, mediating anti-inflammatory effects in the presence of inflammation and driving inflammation in non-inflammatory conditions.

It is likely that D1- and D2-like receptors mediate distinct effects, particularly across different cell types and activation states. The hypothesis that D1-like receptors are inflammatory and the D2-like receptors are anti-inflammatory is very appealing (Nolan and Gaskill, 2019) and is supported in many disease models, as well in a number of in vitro studies (Nakano et al., 2008; Nakano et al., 2011; Shao et al., 2013; Zhang et al., 2015; Osorio-Barrios et al., 2018; Prado et al., 2018; Zhu, Hu et al., 2018; Alam et al., 2021; Yue et al., 2021), many of which are discussed in the previous text. In hMDMs, there is a negative correlation between D1-like receptor expression and the anti-inflammatory cytokine IL-10, whereas dopamine increased IL-1*β* production in hMDMs lacking D2 (Nolan et al., 2019), and D1-like receptor signaling can increase TNF-*α* production in resting human CD4^+^ T-cells (Besser et al., 2005). Pharmacological inhibition of D1-like receptors using SCH23390, or knockout of Drd5 specifically on DCs reduced inflammation in an EAE model (Prado et al., 2018), antagonizing D1-like receptors also suppresses T_h_17 and neutrophilic lung inflammation signaling (Nakagome et al., 2011), both suggesting that D1-like receptor signaling drives inflammation.

In further support of this hypothesis, D2 activation was anti-inflammatory in rodent models of pancreatitis (Han et al., 2017) and acute liver injury (Yue et al., 2021). D2 activation increased the secretion of the anti-inflammatory cytokine IL-10 in human T-cells (Besser et al., 2005), and silencing D2 receptors reduced renal inflammation, which was associated with increased Akt phosphorylation and decreased PP2A activity, suggesting that D2R signaling may act through the activation of PP2A and subsequent inhibition of Akt (Zhang, Jiang et al., 2016). In rodent models of brain injury, the D2 agonist quinpirole protected against glial cell–induced neuroinflammation by inhibiting NF-*κ*B and the activation of *α*B-crystalline (Zhang et al., 2015; Alam et al., 2021). The precise cell type(s) affected by dopamine were not investigated in this study, but subsequent studies showed that the activation of astrocytic D2-like receptors induces *α*B-crystalline to alleviate neuroinflammation in response to MPTP-mediated injury and in stroke models (Shao et al., 2013; Qiu et al., 2016). Moreover, activation of astrocytic D2-like receptors inhibited NLRP3 inflammasome activation via a *β*-arrestin mediated mechanism (Zhu, Hu et al., 2018). While the PP2A/AKT axis has not been examined in astrocytes, *β*-arrestin-mediated recruitment of PP2A is a major mechanism by which D2-like receptors inhibit Akt (Beaulieu et al., 2005, 2015; Beaulieu and Gainetdinov, 2011). This finding suggests an important role of this signaling axis in mediating the anti-inflammatory effects of D2-like receptors.

While these studies and others support the inflammatory D1–anti-inflammatory D2 dichotomy, this concept is not consistent throughout the literature. For example, A68,930 (D1-like receptor agonist) inhibited NLRP3 activation in mouse lung (Jiang, Li et al., 2016), brain (Wang, Nowrangi et al., 2018), and kidney (Cao et al., 2020). Treatment with A77636 also inhibited inflammation in mouse splenocytes (Kawano et al., 2018), and while the effect was not specifically inflammatory, D5 signaling mediated an increase in T-cell activity (Franz et al., 2015). Extensive mechanistic studies show that both types of D1-like receptors individually inhibit NLRP3 activation in several types of activated murine macrophages (Yan et al., 2015; Liu and Ding, 2019; Wu et al., 2020; Liu, Wu et al., 2021). In the NG108-15 rodent glial cell line, D1-like signaling was also associated with the inhibition of NF-*κ*B through PKA-mediated mechanisms, whereas D2-like activation was associated with the activation of NF-*κ*B (Takeuchi and Fukunaga, 2003). D2 activation also increased the release of inflammatory cytokines by human adipocytes (Wang, Villar et al., 2018) and human keratinocytes (Parrado et al., 2012).

While it is possible that some of these differences may be associated with subtype-specific signaling (D5 is anti-inflammatory and D1 is proinflammatory), this seems unlikely, as both D1 (Yan et al., 2015) and D5 (Wu et al., 2020; Liu, Wu et al., 2021) have been shown to decrease inflammation in mouse model systems. Additionally, dopamine receptors of the same subtype can have distinct effects on the same cells, as D2 activation increases IL-10 in resting human T-cells, while D3 activation increases TNF-*α* in the same population (Besser et al., 2005). This bifurcation of dopamine-mediated effects on a dopamine receptor subtype seems to be particularly common in T-cells, in which D3 is more inflammatory and D2 is more anti-inflammatory, although this effect is clearly influenced by activation state. There may also be species-specific differences, such as dopamine being more inflammatory in human myeloid cells than rodent myeloid cells, but many additional studies of human immune cells are needed to determine this effect with any certainty. These data suggest that the effects of dopamine receptor signaling are heavily influenced by existing levels of inflammation and likely have distinct effects on different cell types.

#### 3. Dopaminergic Regulation of Phagocytosis

Phagocytosis is a critical immune function in which cells engulf pathogens or debris and, in the case of pathogens, kill them through intracellular or extracellular mechanisms. Phagocytosis is primarily mediated by professional phagocytes, including monocytes, macrophages, DCs, and eosinophils, although most cell types have the capacity for some kind of phagocytosis. A wide range of studies across species indicate that dopamine can modulate phagocytic activity, but the precise mechanisms by which dopamine mediates these effects seem to differ across species. In *Litopenaeus vannamei*, a species of white prawn, dopamine (10^−7^M–10^−6^ M) significantly decreased gene expression of Drd4, G_i_ heterotrimeric G protein, and exocytosis-related proteins, while phagocytosis-related proteins and G_s_ heterotrimeric G protein were increased (Tong et al., 2020). In the rice stem borer (*Chilo suppressalis*), dopamine (10^−5^M) increased hemocyte phagocytosis via a D1-like receptor. This effect appeared to involve ERK1/2 activation (Wu et al., 2015), which can be induced by dopamine via cAMP/PKA signaling (Zhen et al., 1998; Gerits et al., 2008; Shao et al., 2020). While the implications of these findings to human subjects is unclear, these data suggest that the capacity of dopamine to stimulate the D1–cAMP axis may promote the phagocytic function of immune cells via the G_s_ heterotrimeric G protein pathway, which signals through the activation of adenylate cyclase and the production of cAMP (Marinissen and Gutkind, 2001).

Studies also suggest that dopamine modulates phagocytic activity in a number of vertebrate systems, although the mechanisms underlying this activity are not clear. In wall lizard splenic macrophages, dopamine induced bimodal changes in phagocytosis; higher concentrations of dopamine (10^−7^–10^−5^M) could decrease phagocytosis, while very low dopamine concentrations (10^−11^–10^−15^M) could increase phagocytosis (Roy and Rai, 2004). Dopamine (10^−8^–10^−7^M) increased the phagocytic activity of murine BMDMs in the presence of IFN-*γ*, as well as in neutrophils (Sookhai et al., 1999), although another study showed that similar concentrations of dopamine (6.5 × 10^−7^M) reduced neutrophil phagocytosis (Wenisch et al., 1996). In mice, macrophage phagocytosis was decreased by spiperone (D2-like receptor antagonist) (Sternberg et al., 1987). Chicken macrophages treated with dopamine (0.65–1.6 × 10^−6^M) and guinea pig macrophages treated with bromocriptine (D2 receptor agonist) and pergolide (D2 receptor agonist) both showed an enhanced binding and phagocytosis of IgG-sensitive red blood cells (Ali et al., 1994; Gomez et al., 1999).

In each of these studies, changes in phagocytic activity were associated with dopamine receptor-induced changes in the expression of surface Fc-gamma receptor (Fc*γ*) receptors, which are critical regulators of the immune response that mediate a number of factors including antigen uptake (Sternberg et al., 1987). Dopamine-mediated changes in Fc*γ* receptors could not only disrupt phagocytosis and antigen presentation but also suggest a mechanism by which dopamine affects T-cell activation and promotes the development of autoimmunity (Junker et al., 2020). A D2-mediated effect on phagocytosis was seen in rodent peritoneal macrophages, but antagonizing D2-like receptors with domperidone increased phagocytosis in lactating rats (Carvalho-Freitas et al., 2008). Unlike studies showing effects through regulation of Fc*γ* expression, this effect was thought to be mediated by the regulation of serum prolactin levels, and phagocytosis was shown to be decreased at other time points in another study by this group (Carvalho-Freitas et al., 2008, 2011). Dopaminergic regulation of phagocytosis was also seen in hMDMs when DAT activity was blocked with nomifensine, downregulating LPS-induced phagocytic activity via autocrine signaling and suggesting that lower dopamine levels inhibit phagocytic activity in activated macrophages (Mackie et al., 2022). A decrease in phagocytosis, as well as ERK1/2 phosphorylation, was also seen in in response to dopamine (2 × 10^−6^M) treatment of LPS-activated BV2 microglia, but no effect on the phagocytic activity of resting cells was observed, suggesting autocrine control of phagocytosis by dopamine signaling in activated myeloid cells (Fan et al., 2018).

These differences could be due to the different mechanisms through which phagocytosis is regulated, such as Fc*γ* receptors, prolactin levels, or DAT/MAPK. Lower dopamine concentrations (≤10^−6^M) and D2 activation increased phagocytosis (Sternberg et al., 1987; Ali et al., 1994; Gomez et al., 1999) and were associated with changes in Fc*γ* receptors, while reducing D2 activity increased phagocytic activity and was associated with prolactin (Carvalho-Freitas et al., 2008, 2011). However, phagocytic activity was also reduced by the pan-dopamine agonist apomorphine, the D2/D3 agonist bromo-a-ergocryptine, and the *β*-adrenergic receptor antagonist propranolol in wall lizard macrophages. These data studies suggest that D1-like receptors can also drive phagocytosis, and many of these data suggest the involvement of the cAMP signaling pathway, as D2 activation inhibits cAMP production.

In support of this, in the wall lizard cells, treatment with phosphodiesterase IBMX, which increases cAMP levels, resulted in a greater reduction in phagocytosis when combined with dopamine. Signaling through MAPK is also downstream of cAMP in a number of pathways. However, the use of the cAMP analog db cAMP had a similar biphasic effect (Roy and Rai, 2004), making the directional effects of higher and lower cAMP levels unclear. Moreover, dopamine-mediated cAMP activity was notably absent from human macrophages (Nickoloff-Bybel et al., 2019), suggesting that this pathway might not function in human cells. Taken together, these data form a confusing picture of dopaminergic modulation of phagocytosis, suggesting that the differences in the effects of dopamine are due to the concentration of dopamine or ligand being used, the exposure time, the receptor being activated, and the species. Thus, while these data indicate that dopamine does regulate phagocytic function in multiple cell types, additional work with carefully chosen and controlled experimental conditions is needed to precisely define the mechanism by which this regulation occurs.

#### 4. Dopaminergic Regulation of Chemotaxis

Chemotaxis is the mechanism by which cells move in response to extracellular chemical gradients, allowing immune cells to migrate to sites of inflammation or tissue damage when they detect chemokines and other factors secreted by cells at that site. Chemotaxis involves many chemokines, some of which are species- or cell-specific, and is initiated when soluble chemokines bind to their cognate receptors on immune cells (Rossi and Zlotnik, 2000; Hughes and Nibbs, 2018). A number of studies have shown that dopamine can impact the production of chemotactic factors, particularly CCL2, which is important in the pathogenesis of many chronic inflammatory conditions, as well as neurologic HIV (neuroHIV) (Eugenin et al., 2006; Covino et al., 2016; Fantuzzi et al., 2019; Das et al., 2021). Dopamine increased the mRNA expression of CXCL8 and CCL2 in activated PBMCs (Torres et al., 2005) and the injection of a D1-like agonist increased the mRNA expression levels of CCL2 and C-C motif chemokine ligand 7 in the rodent PFC (Saika et al., 2018). In hMDMs, dopamine (10^−6^M–10^−8^M) increased the production of CCL2, CXCL8, CXCL9, and CXCL10 (Gaskill et al., 2012; Nolan et al., 2019), while blocking DAT activity potentiated LPS-induced CCL2 production (Mackie et al., 2022). Both CCL2 and CXCL2 were reduced by D2 signaling in acute pancreatitis (Han et al., 2017; Saika et al., 2018), and in mouse renal proximal tubule cells, D2 knockdown was associated with an increase in CCL2 production. This effect was connected to the loss of PP2A activity, suggesting that the D2-like receptors may inhibit cytokine production via PP2A and Akt inhibition (Zhang, Jiang et al., 2016). In addition to CCL2, in primary murine splenocytes, peritoneal macrophages and NK cells, dopamine (10^−5^–10^−6^M) increased CXCL1 production (Kawano et al., 2018).

In addition to increasing the production of chemokines, dopamine itself can act as a chemoattractant and potentiate chemotaxis and chemokinesis. In neutrophils, high concentrations of dopamine (10^−4^ and 10^−5^ M) inhibited IL-8 mediated transendothelial migration (Sookhai et al., 2000). In mature human CD14^+^CD16^+^ monocytes, dopamine (10^−6^M–5 × 10^−5^M) and SKF38393 (D1-like agonist) increased chemokinesis and transmigration across a model of the blood–brain barrier (Coley et al., 2015; Calderon et al., 2017), suggesting a chemotactic role for D1-like receptors. A role for D1-like receptors, specifically D5, was also shown in murine CD4^+^ T-cells in the gut, as the formation of a heteromeric CCR9:D5 complex on these cells mediated inflammation-induced T-cell migration into the gut mucosa. These effects were mediated by ERK1/2 signaling and were specific to CCR9, as D5 did not form heteromers with other chemokine receptors (Osorio-Barrios et al., 2021).

D2-like receptor signaling has also been implicated in dopamine-mediated chemotaxis. In mice, D3 stimulation of T_regs_ reduced CCR9 expression and inhibited the migration of these cells into the lamina propria region of the gut upon intestinal inflammation (Ugalde et al., 2021). In naïve CD8^+^ T-cells, dopamine (10^−7^M) potentiated homing to CCL19, CCL21, and CXCL12 through D3 and G*_α_*_i_-mediated Ca^2+^ mobilization (Watanabe et al., 2006), and treating peripheral T-cells from head and neck cancer patients with dopamine (10^−8^M) increased spontaneous migration and migration toward CXCL12 (Saussez et al., 2014). A role for D2-mediated G*_α_*_i_ signaling is supported by experiments using HEK293 cells transfected with D2-receptors. Treating these cells with quinpirole (D2-agonist) mediated a chemotactic response to IL-8 that required the activation of G*_α_*_i_ and G*_βγ_* activity, while G*_α_*_s_ and G*_α_*_q_ receptors did not mediate these effects (Neptune and Bourne, 1997).

In addition to driving immune cell chemotaxis, dopamine can also influence inflammation and immune activity by modulating the movement of bacteria and pathogens. Antagonizing D1- and D2-like receptors blocked liver fluke (*Clonorchis sinensis*) larva chemotaxis into the bile duct, indicating that dopamine plays a role in the development of clonorchiasis (Dai et al., 2020). High levels of dopamine (5 × 10^−5^M) also increased the expression of flagellar motility-related genes and the swimming motility of the aquatic bacterium *Vibrio harveyi*, a Gram-negative bacterium that is a major pathogen of all aquatic organisms. This effect was blocked by chlorpromazine, suggesting that this effect was driven by D2-like receptors, although the effect could be influenced by the many effects of chlorpromazine on nondopaminergic receptors (Yang, Anh et al., 2014). Dopamine has biphasic effects on the chemotaxis of *E. coli*, repelling these bacteria at concentrations below 10^−4^M but attracting *E. coli* at higher concentrations. Although gut concentrations of dopamine are <10^−4^M at most times, higher concentrations are likely present in the microenvironment around the gut lumen and at the mucous layer where these compounds are secreted, and different foods could also elevate gut dopamine levels (Lopes and Sourjik, 2018; Matt and Gaskill, 2020).

Taken together, these data implicate several signaling pathways in the regulation of dopaminergic chemotaxis and suggest that many of the chemotactic effects of dopamine could be mediated through CCL2, CXCL9, and CXCL12. It is difficult to identify patterns relating to cell type, as D1-like activity promoted chemotaxis in monocytes, T-cells, and *E. coli*, while D2-like receptors drove chemotaxis in T-cells and other pathogens. This indicates that both D1- and D2-like receptor signaling can play a role in chemotactic activity. Pathways mediated by G*_α_*_i_, G*_βγ_*, and Akt primarily associated with D2-like receptors, while ERK1/2 and Ca^2+^ flux could be mediated by either receptor subtype. As with many functions, these effects are likely different in distinct cell types and activation states, but the current data make it difficult to further define this, as both types of receptors act on multiple cell types in both resting and activated states.

#### 5. Dopaminergic Regulation of Oxidative Burst and Nitric Oxide Production

In addition to regulating the production of inflammatory mediators, phagocytic activity, and chemotaxis, dopamine also influences respiratory or oxidative bursts, as well as ROS and NO production. These bursts mediate the rapid release of ROS such as hydrogen peroxide (H_2_O_2_) and superoxide anion, which can be formed by the activity of superoxide dismutase. This process is common in phagocytes, such as myeloid cells and granulocytes, to degrade internalized bacteria and other particles as part of the immune response. These bursts can also affect cell signaling (Forman and Torres, 2002; Dahlgren and Karlsson, 1999). In neutrophils, dopamine may reduce respiratory bursts, as dopamine treatment (6.5 × 10^−7^M) of human neutrophils reduced ROS production (Wenisch et al., 1996), and high concentrations of dopamine (10^−4^ and 10^−5^ M) inhibited superoxide anion production in response to N-formylated N-formyl-methionyl-leucyl-phenylalanine stimulation (Yamazaki et al., 1989; Matsuoka, 1990). Similarly, high levels of dopamine (10^−4^M) and fenoldopam (D1-like agonist) reduced respiratory bursts in neutrophils isolated from the blood of patients with systemic inflammatory response syndrome (Sookhai et al., 1999). Lower concentrations of dopamine (2.61 × 10^−7^M or 2.61 × 10^−10^M) had no effect on the formation of H_2_O_2_-mediated oxidative bursts in human neutrophils (Trabold et al., 2007).

In myeloid cells such as rodent peritoneal macrophages, antagonizing D2-like receptors with domperidone increased spontaneous oxidative bursts of H_2_O_2_ (Carvalho-Freitas et al.,2008, 2011). Additionally, dopamine treatment (10^−6^–10^−8^M) of hemocytes from the white shrimp *Litopenaeus vannamei* significantly reduced respiratory bursts and superoxide dismutase activity (Cheng et al., 2005). These data suggest that dopamine generally inhibits the production of ROS and oxidative bursts in neutrophils, particularly at higher levels. This is supported by recent data showing exposure to dopamine (10^−6^–10^−9^M) broadly inhibited neutrophil activity in a D1-dependent manner (Marino et al., 2022). Further, data from hypertension studies showing that the activation of D1-like receptors, particularly D5, reduced the production of ROS in mitochondria through an autophagy-associated mechanism (Yang et al., 2006; Lee et al., 2021). In addition, studies showed that knocking out Drd2 in renal tubule cells increased renal ROS production (Yang, Cuevas et al., 2014) and that Drd2 knockout mice had increased levels of ROS due to aldosterone dysregulation (Armando et al., 2007).

Dopamine has also been shown to dysregulate NO production. NO is important in defense against infectious diseases, tumors, sterile inflammation, and other insults. NO is produced by several types of immune cells, mostly macrophages and granulocytes (Bogdan, 2001). In rodents, treatment with apomorphine (pan-dopamine receptor agonist) increased NO_2_^−^ and NO_3_^−^ in dialysate from the hypothalamus (Melis et al., 1996), suggesting that dopamine increases NO production. However, this conclusion is opposed by an array of in vitro studies. In LPS-induced primary rodent microglia and BV-2 cells, dopamine (2 × 10^−6^M) increased NO synthase (Fan et al., 2018). Inhibiting D1-like receptors with SCH23390 and D2-like receptors with sulpiride inhibited NO production by LPS-stimulated peritoneal macrophages (Hasko et al., 1996). Dopamine also enhanced LPS-mediated NO production in RAW264.7 cells, although only at higher concentrations (5 × 10^−5^M) and to a much lesser extent than other catecholamines (Chi et al., 2003). However, dopamine decreased the production of NO and NO synthase in LPS-stimulated primary rodent microglia as well as N9 and BV2 murine microglia through D1- and D2-like receptors (Chang and Liu, 2000; Farber et al., 2005; Yoshioka et al., 2016; Wang, Chen et al., 2019). These findings opposed other studies that suggested that dopamine increased NO production, but data from BV2 cells suggest that this could result from cytotoxicity due to the production of dopamine quinones (Beck et al., 2004) and may not be mediated by dopamine receptor signaling. These data suggest more research is needed in this area, but that dopamine receptor signaling may promote the production of NO in myeloid cells, although cytotoxic dopamine quinone formation may have the opposite effect.

#### 6. Signaling Mechanisms Mediating Dopaminergic Immunomodulation

The discrete effects of specific dopamine receptors may also reflect D1- and D2-like receptor signaling through both convergent and divergent signaling cascades, depending on the cell system and dopamine receptors activated. For example, Akt inhibition via PP2A recruitment has been suggested to be central in dopamine-mediated anti-inflammatory effects in isolated cell systems and disease models (Tolstanova et al., 2015; Zhang, Jiang et al., 2016; Han et al., 2017; Wu et al., 2020; Yue et al., 2021). Signaling through Akt regulates a wide range of cellular processes, including cell growth, survival, metabolism, and inflammation (Manning and Toker, 2017). In the immune response, Akt modulates NF-*κ*B activity by regulating IkappaB (I*κ*B) kinase, which phosphorylates I*κ*B to release and activate NF-*κ*B (Dan et al., 2008; Cheng and Kane, 2013; Dorrington and Fraser, 2019). This suggests the dopamine-mediated inhibition of Akt could reduce inflammatory cytokine production. Akt inhibition is generally associated with D2-like receptor signaling (Beaulieu et al., 2004, 2007; Zhang, Jiang et al., 2016; Han et al., 2017; Zhu, Hu et al., 2018; Wu et al., 2020), but D1-like receptors can act on AKT (Beaulieu et al., 2005). The activity of this pathway, and the relative frequencies of D2- and D1-like receptors may explain the anti-inflammatory activity of these receptors in certain systems. Both D1- and D2-like receptors can also activate the phosphatidylinositol 3-kinase/Akt signaling cascade, although the immunologic impact of dopamine-mediated activation of this pathway is not well understood (Zhen et al., 2001; Brami-Cherrier et al., 2002; Nair et al., 2003; Nair and Sealfon, 2003; Iwakura et al., 2008; Mannoury la Cour et al., 2011; Chen, Ruan et al., 2012; Perreault et al., 2013; Radl et al., 2013; Mirones et al., 2014; Yan et al., 2020).

Some of the differences in the effects of dopamine on similar cell types may be due to variations in the capacity of D1-like receptors to activate the canonical D1–G*_α_*_s_–cAMP signaling pathway. While studies have shown that cAMP signaling has both inflammatory and anti-inflammatory effects, in myeloid cells, cAMP signaling is largely associated with a decrease in inflammation (Serezani et al., 2008; Peters-Golden, 2009; Gerlo et al., 2011). Supporting this hypothesis, dopamine-mediated inhibition of NLRP3 in murine macrophages was linked to cAMP activation, and dopamine-mediated inhibition of NF-*κ*B was associated with D1-mediated activation of PKA, a downstream effector of cAMP, in microglia and other cells lines (Takeuchi and Fukunaga, 2003; Yan et al., 2015; Wang, Chen et al., 2019). In contrast, in hMDM where dopamine activates NF-*κ*B and primes the inflammasome (Nolan et al., 2020), D1-like receptors do not act through the G*_α_*_s_-cAMP pathway (Nickoloff-Bybel et al., 2019). In T-cells, stimulation of D3 reduces cAMP, which increases naïve CD4^+^ T-cell activation (Franz et al., 2015), further suggesting that a lack of cAMP/PKA signaling supports dopamine-mediated inflammatory effects.

Differences in the capacity of distinct dopamine receptors to activate the MAPK cascade may also contribute to variations in the effects of dopamine. The MAPK signaling cascade modulates numerous cellular functions, including inflammatory cytokine release, in part via the activation of transcription factors such as activator protein 1 (Karin, 1995; Kaminska et al., 2009; Furler and Uittenbogaart, 2010; Cargnello and Roux, 2011; Kyriakis and Avruch, 2012). Dopamine can activate all members of the MAPK family through both D1- and D2-like receptors, although the precise mechanism is unclear and varies from system to system (Luo et al., 1998; Choi et al., 1999; Zhen et al., 2001; Beom et al., 2004; Wang et al., 2005; Lee et al., 2006; Huang et al., 2012; Franz et al., 2015). Many studies have connected the capacity of dopamine to regulate different members of this family with its effects on inflammation. In naïve CD4^+^ T-cells, D3 and D5 mediate ERK1/2 phosphorylation and promote the differentiation of inflammatory T_h_1 cells (Franz et al., 2015). However, in DCs, D5 stimulation downregulated LPS-induced ERK1/2 phosphorylation but did not affect JNK or p38 MAPK phosphorylation. The D5-mediated reduction in ERK1/2 was associated with decreased IL-12 and IL-23 production in specific DC subsets, suggesting an inhibitory effect (Prado et al., 2012). Similarly, in primary microglia and BV-2 microglia, dopamine downregulated ERK phosphorylation, while in resting microglia dopamine increased p38 MAPK activity (Fan et al., 2018). Interestingly, in rat astrocytes, D1-like activation of ERK1/2 was associated with cell migration (Huang et al., 2012), supporting differences in the effects of dopamine-induced MAPK signaling between disparate cell types. Indeed, studies have shown both cell-type and receptor-specific differences in D2-like receptor-mediated ERK activation (Beom et al., 2004; Wang et al., 2005). As with cAMP and Akt signaling, this suggests discrete effects of dopamine receptor subtypes on MAPK activation, which may mediate the effects of dopamine on the inflammatory responses in these cells.

Overall, the data discussed in this section of the review indicate that dopamine influences a wide array of immune functions in both an inflammatory and anti-inflammatory manner. Data on most of these functions is relatively sparse, owing to the large variations in experimental design (species, dopamine/ligand concentration, disease model) and potential cell type– and species-specific differences in dopamine receptor expression and signaling bias. Thus, there is no clear consensus on the roles of distinct dopamine receptor subtypes in mediating these effects. The hypothesis that D1-like receptors are inflammatory while D2-like are anti-inflammatory is not broadly supported by the data, although there are certainly specific cell types or diseases, which will be discussed in depth in the following sections, in which this may be the case. Evidence suggests that dopamine may be more anti-inflammatory in stimulated cells and inflammatory in resting cells, and studies in human cells generally show an inflammatory effect of dopamine, although this may be due to the smaller numbers of studies done in humans relative to other systems. Data also suggest that the effects of specific dopamine receptors may differ between cell types and among the same cell type in different species. Signaling pathways activated by dopamine receptors are also likely to overlap, and the activation of multiple dopamine receptor subtypes could interact and result in opposing, additive, or novel outcomes.

Moving forward, careful evaluation of dopamine receptor expression in each model system, defining both raw receptor expression and the ratios of different dopamine receptors, is important in precisely defining the receptors and signaling mechanisms that mediate a particular effect. Many areas warrant further investigation, including the understanding of dopamine release in immune cells, examination of cell type–specific dopamine receptor signaling and specific immune activity, and delineation of dopamine-mediated inflammation from inflammation mediated by pharmacologic drugs that modify dopamine signaling. Broadly, these data show the importance of dopamine in the regulation of immune function and the need for further studies with carefully considered experimental designs to better understand and leverage dopaminergic immunomodulation for disease treatment. In the subsequent sections, we will discuss how the immunomodulatory effects discussed here impact different organs and diseases, highlighting the potential for dopaminergic immunomodulation in the treatment of a wide array of pathogenic conditions.

## IV. Regional and Disease-Specific Effects of Dopamine

### A. Central Nervous System

#### 1. Introduction

While further research on the signaling processes and cell type–specific effects induced by dopamine is important for understanding the basic processes that mediate dopaminergic immunomodulation, it is also important to consider the application of this research to specific pathologies. Fluctuations in dopamine and the dopaminergic system influence a wide variety of organ systems and diseases. Prominent among these are numerous neurologic conditions including PD and neuroHIV, neuropsychiatric conditions such as schizophrenia and depression, and developmental neurologic disorders such as ADHD and epilepsy. Many of the studies evaluating the impact of dopamine on these diseases can be found in Table 2. This is not an exhaustive list of neuropathologies that could be influenced by dopamine, and substantial research has been devoted to examining other neurologic conditions, such as MS (Pacheco et al., 2014; Levite et al., 2017) and Alzheimer’s disease (Thomas Broome et al., 2020).

**TABLE 2:** Effects of dopaminergic immunomodulation associated with disease

Species	Cell type/region	Dopamine receptors/proteins involved	Dopamine/dopamine modifying agent or method	Immunologic finding	Method of detection	Reference
NeuroHIV						
Human	Macrophage (HIV-infected monocyte-derived)	D1-like	Meth (1–250 *μ*M) SCH23390 (10 *μ*M)	↑ HIV replication and ↑ CCR5 with Meth (effect blocked by SCH23390)	RT-PCR ELISA Western blot	Liang et al., 2008
Human	Macrophage (HIV-infected ART-treated monocyte-derived macrophage)	N/A	Dopamine (1 nM–1*μ*M)	↑ IL-1*β*, IL-6, IL-18, CCL2, CXCL8, CXCL9, CXCL10	AlphaLISA	Nolan et al., 2019
Human	Macrophage (monocyte-derived macrophages)	D1-like D2-like	Dopamine (1 nM–1*μ*M)	↑ HIV entry by increasing calcium release with dopamine	Viral entry assay Immunofluorescence	Nickoloff-Bybel et al., 2019
Mouse	Macrophage (bone marrow–derived macrophages from transgenic nef mice) Brain (striatum)	DAT	N/A	↓ Striatal dopamine and DAT levels in HIV nef mice ↑ ccl2 gene expression in striatum and cortex ↑ ccl2 and ↓ ifn-*α* gene expression in BMDM ↑ ccl2 gene expression in striatum	qPCR IHC Western blot HPLC	Acharjee et al., 2014
Human	Monocyte (CD14^+^CD16^+^ Peripheral blood mononuclear cells)	D1-like	Dopamine (1–50 *μ*M) SKF38393 (1–50 *μ*M)	↑ Monocyte migration across the blood–brain barrier from HIV infected individuals with dopamine and SKF38393	Flow cytometry	Calderon et al., 2017
Primate	Brain (SIV infected frontal cortex, basal ganglia and SbN)	MAO D1-like D2-like	Selegiline (0.01–2 mg/kg intramuscularly) L-DOPA (50 mg/kg orally)	↑ SIV expression and TNF-*α* with selegiline and L-DOPA	In situ hybridization	Czub et al., 2004
Human	Dendritic cell (HIV-infected human PBMC-derived dendritic cells)	D2	Meth (10–100 *μ*M) Sulpiride (100 *μ*M)	↑ HIV replication and phospho-p38-MAPK with Meth ↑ CCR5 and CXCR4 with Meth ↓ Viral replication and CCR5 with drd1 KD and sulpiride	MAGI assay ELISA RT-PCR Western blot	Nair et al., 2009
Human	Macrophage (primary monocyte-derived macrophages)	D1-like D2-like	Dopamine (0.01–10 *μ*M) Flupentixol (1 *μ*M)	↑ HIV entry in the presence of dopamine (blocked by flupentixol)	Viral entry assay	Gaskill et al., 2014
Human	Macrophage (primary monocyte-derived macrophages)	D1-like D2-like	Dopamine (1 *μ*M)	↑ HIV replication with dopamine Dopamine alters effectiveness of maraviroc on HIV infection	AlphaLISA	Matt et al., 2021
Human	Microglia (C06 microglia and iPSC-derived microglia)	D1-like D2-like	Dopamine (1 *μ*M)	↑ HIV replication with dopamine	AlphaLISA	Matt et al., 2021
Human	T-lymphoblast (ACH-2 cell line)	Dopamine dependent oxidative stress	Dopamine (20–100 *μ*M) Glutathione (1–2 mM)	↑ HIV in chronically infected T lymphoblasts in the presence of dopamine (effects attenuated by Gluthiatione)	Flow cytometry	Scheller et al., 2000
Human	Monocyte (CD14^+^CD16^+^ peripheral blood mononuclear cells)	D1-like	Dopamine (100 nM–1 *μ*M) SKF38393 (1 nM–100 nM)	↑ Monocyte migration (chemokinesis) with dopamine and SKF38393 ↑ monocyte adherence with dopamine	Migration assay Spreading and adhesion assay	Coley et al., 2015
Human	T-cell (Jurkat cell line)	N/A	Dopamine (10–300 *μ*M)	↑ HIV-1 gene transcription in presence of dopamine	Chloramphenicol acetyltransferase assay	Rohr, Sawaya et al., 1999
Human	Peripheral blood mononuclear cells	N/A	Dopamine (100 *μ*M)	↑ HIV-1 gene transcription in presence of dopamine	Chloramphenicol acetyltransferase assay	Rohr, Sawaya et al., 1999
Human	Macrophage (monocyte-derived macrophages)	D2-like	Dopamine (20 *μ*M) Quinpirole (1 *μ*M)	↑ HIV-1 replication via ERK1 phosphorylation with dopamine ↑ Number of virally infected macrophages with dopamine ↑ HIV replication with quinpirole	ELISA Immunofluorescence Western blot	Gaskill et al., 2009
PD						
Mouse	T-cell (CD4^+^ T-cells from MPTP-treated mice)	D2	Sumanirole (1 *μ*M) L-741,226 (1 *μ*M)	↑ MPTP-induced neuropathology and motor impairment in drd2-KO ↑ T_h_1/T_h_17 phenotype with drd2-KO ↓ T_h_1/T_h_17 phenotype with Sumanirole in vitro (reversed by L-741,226) ↑ CD11b, TNF-*α*, IL-1*β*, IFN-*γ*, IL-17A in drd2-KO MPTP-treated mice	Flow cytometry Western blot RT-PCR	Liu, Zhai et al., 2021
Mouse	Microglia (MPTP-treated mice) Brain (substantia nigra)	D3	N/A	drd3-KO is neuroprotective in SbN of MPTP-treated mice and ↓ microglia activation	Flow cytometry	González et al., 2013
Mouse	T-cell (CD3^+^ T-cells from MPTP-treated mice)	D3	PD 128907 (50 nM in vitro)	↑ Percentage of Foxp3^+^ in drd3-KO cells ↓ IL-2, TNF-*α*, and IFN-*γ* in drd3-KO cells ↑IL-2 with PD 128907	ELISA Flow cytometry	González et al., 2013
Human	Lymphocyte (peripheral blood of untreated early PD patients)	D3	N/A	↓ drd3 gene expression	qPCR	Kim, Nigmatullina et al., 2019
Mouse	Lymphocyte (MPTP-treated mice)	D3	N/A	↑ drd3 gene expression	qPCR	Kim, Nigmatullina et al., 2019
Human	T-cell (peripheral blood mononuclear cells from PD patients and healthy donors)	D3	N/A	↓ D3 receptor expression levels in CD4^+^ T-cells from PD patients and higher frequency of T_h_1 polarization	Flow cytometry	Elgueta et al., 2019
Mouse	T-cell (CD4^+^ T-cells from MPTP-treated mice)	D3	PG01037 (30 mg/kg)	↓ Microglial activation, motor impairment and neurodegeneration in MPTP-treated mice given PG01037 Transference of CD4^+^ T-cells that were treated ex vivo with PG01037 led to ↓ microglial activation and motor impairment	Flow cytometry	Elgueta et al., 2019
Human	Lymphocyte (peripheral blood mononuclear cells of PD patients)	D3	[^3^H]7-OH-DPAT	↓ drd3 gene expression and inversely correlated with disease severity ↓ D3 receptor binding sites on lymphocytes of PD patients	qRT-PCR Receptor binding assay	Nagai et al., 1996
Human	Lymphocyte (peripheral blood lymphocytes from PD patients)	D2	[^3^H]spiroperidol (0.1–7nM)	73% ↓ in [^3^H]spiroperidol binding sites in PD patients	Receptor binding assay	Le Fur et al., 1980
Human	Lymphocyte (peripheral blood lymphocytes from PD patients and healthy controls)	D1-like D2-like	[^3^H] SCH 23390 [^3^H] 7OH-DPAT	↑ Density of D1-like and D2-like receptors on PBLs of PD patients prior to treatment (reversed following L-DOPA or bromocriptine therapy)	Receptor binding assay	Barbanti et al., 1999
Human	Lymphocyte (peripheral blood lymphocytes from PD patients and healthy controls)	TH	N/A	↓ TH immunoreactivity in PD patients ↓ intracellular DA concentration in PD patients	Tyrosine hydroxylase immunoreactivity assay HPLC	Caronti et al., 1999
Human	Lymphocyte (peripheral blood lymphocytes from PD patients and healthy control)	DAT	N/A	↓ DAT immunoreactivity in lymphocytes of early PD patients	Immunohistochemistry	Caronti et al., 2001
Human	Lymphocyte (peripheral blood lymphocytes from PD patients and healthy control)	DAT	N/A	↓ DAT immunoreactivity in lymphocytes of early PD patients	Densitometric analysis	Pellicano et al., 2007
Mouse	Striatum (MPTP-treated mice)	N/A	Rotigotine-loaded microspheres (10 mg/kg) or rotigotine (0.7 mg/kg)	↓ TNF-*α*, IL-1*β*, IL-6	Immunohistochemistry ELISA	Yu et al., 2015
Human	T-cells (CD4^+^ naïve T-cells and memory T-cells of PD patients and healthy controls)	D1-like D2-like	N/A	↓ D1-like receptors on naïve CD4^+^ T-cells of PD patients ↑ D2-like receptors on memory T-cells of PD patients	qPCR Flow cytometry	Kustrimovic et al., 2016
Schizophrenia						
Human	Peripheral blood mononuclear cells (patients diagnosed with schizophrenia /schizophreniform disorder)	D2 D3	N/A	↑drd3 gene expression compared with controls drd2 gene expression correlated with positive symptom scores	qPCR	Cui et al., 2015
Human	T-cell (peripheral blood mononuclear cells from patients diagnosed with schizophrenia)	D3 D4	N/A	↑ drd3 and ↓ drd4 gene expression on T-cells	RT-PCR	Boneberg et al., 2006
Human	T-cell (CD4^+^ peripheral blood mononuclear cells from patients diagnosed with schizophrenia)	D3	N/A	↑ D3 receptor expression in CD4^+^ T-cells of clozapine-treated schizophrenia patients	Flow cytometry	Fernandez-Egea et al., 2016
Human	T-cell (peripheral blood mononuclear cells from patients diagnosed with schizophrenia)	D2 D4	N/A	↑ D2 receptor expression on CD8^+^ T-cells and correlated positively with symptom scores ↓ D2 receptor expression on CD4^+^ T-cells ↑ D4 receptor expression on CD4^+^ T-cells and inversely correlated to symptom scores	Flow cytometry	Brito-Melo et al., 2012
Human	T-cell (peripheral blood T lymphocytes from patients diagnosed with schizophrenia)	D3 D5	N/A	↑ drd3 and drd5 gene expression in lymphocytes of drug naïve patients with schizophrenia compared with treated patients	RT-PCR	Kwak et al., 2001
Human	Serum (children with first episode of psychosis)	D2	N/A	IgG and IgM DRD2 antibodies were present in children with first episode of psychosis	Flow cytometry	Pathmanandavel et al., 2015
Human	Lymphocyte (peripheral blood lymphocytes from patients diagnosed with schizophrenia)	D2	N/A	↑ D2 receptor expression	Receptor binding assay	Bondy et al., 1985
Human	Lymphocyte (peripheral blood lymphocytes from patients diagnosed with schizophrenia)	D3	N/A	↑ drd3 gene expression	RT-PCR	Ilani et al., 2001
Human	Lymphocyte (peripheral blood lymphocytes from patients diagnosed with schizophrenia)	D3	N/A	↓ drd3 gene expression	RT-PCR	Vogel et al., 2004
Human	Lymphocyte (peripheral blood lymphocytes from patients diagnosed with schizophrenia)	DAT	N/A	↓ DAT receptor expression	Receptor binding assay	Marazziti et al., 2010
Bipolar disorder						
Mouse	Brain (striatum, PFC, and hippocampus)	DAT	GBR12909	↑ IL-2, ↓ IL-6, and ↑ BDNF in PFC ↑ IL-2 and IL-4, ↓ IL-6, ↑ IL-10 and IFN-*γ* in striatum ↑ IL-4 and IFN-*γ*, ↓ IL-10 in hippocampus	Cytokine binding assay ELISA	Bastos et al., 2018
Human	Lymphocyte (peripheral blood T lymphocytes of patients diagnosed with bipolar disorder)	D3	N/A	↓ drd3 gene expression in patients with bipolar disorder compared with healthy controls	RT-PCR	Vogel et al., 2004
Human	Lymphocyte (peripheral blood lymphocytes from patients diagnosed with bipolar disorder)	DAT	N/A	↓ DAT receptor expression in patients diagnosed with bipolar disorder compared with healthy controls	Receptor binding assay	Marazziti et al., 2010
Depression						
Human	Lymphocyte (peripheral blood lymphocytes from patients diagnosed with major depression)	D4	N/A	↓ drd4 gene expression in patients with major depression	RT-PCR	Rocc et al., 2002
Primate	Brain	D2	N/A	↓ D2 receptor binding and striatal dopamine release with chronic interferon-alpha administration in association with anhedonia-like behavior	PET imaging	Felger et al., 2013
Anxiety						
Mouse	Myeloid cell (CD11b+CD45+ immune cells from brain tissue)	D1	Modafinil (90 mg/kg) SCH23390 (0.1 mg/kg)	↓ LPS-induced immune cell infiltration in the brain with Modafinil ↓ il1b gene expression in stressed mice treated with Modafinil (blocked by SCH23390)	Flow cytometry qPCR	Zager et al., 2018
Human	T-cell (peripheral blood T lymphocytes from patients diagnosed with GAD)	N/A	Dopamine (1 *μ*M)	↓ IL-2 and IFN-*γ* in dopamine treated PHA activated T-cells ↓ IL-10 and TGF-*β* but ↑ IL-17 and IL-21 in dopamine treated PHA activated T-cells from patients with GAD ↑ IL-6 and TNF*α* in dopamine treated PHA activated T-cells from both healthy controls and patients with GAD	ELISA	Ferreira et al., 2011
ADHD						
Human	Serum (children diagnosed with ADHD)	DAT	N/A	Individuals carrying DAT 10/10 genotype exhibited higher DAT auto-antibodies levels than healthy controls which correlated with disease severity ↓ DAT auto-antibody levels comparable to healthy controls in ADHD patients treated with methylphenidate	ELISA qPCR	Giana et al., 2015
Mouse	Brain (prefrontal cortex, NAc, VTA of male adult mice)	D1 D2	N/A	Locomotor hyperactivity with alterations in D1 and D2 receptor profile in the brain of male mice exposed to ↑ early prenatal stress	qPCR	Bronson and Bale, 2014
Mouse	Choroid plexus (microglia, monocyte)	DAT	Amphetamine (1.5 mg/kg)	Dopamine transporter hypofunction ↓ monocyte-macrophage infiltration into the brain and was associated with ↓ microglial activation (COX-2, TNF-*α* and IRF7)	Histochemistry Flow cytometry qPCR	Castellani et al., 2019
Epilepsy						
Human	Peripheral blood immune cells from patients diagnosed with TLE	D1 D2 D4 D5	N/A	↑ D2 expression in T lymphocytes of patients diagnosed with TLE ↑ D1, D2, D4, and D5 receptor expression on monocyte and granulocytes of patients with TLE	Flow cytometry	Vieira et al., 2021
Colitis and inflammatory bowel disease						
Mouse	T-cell [splenic and lymph node T-cells from DSS-induced colitis model (drd5-KO and Rag1-KO)]	D5	SFK81297 (10–1000 nM)	↓ CD4^+^ T-cell migration into gut lamina propria without impacting survivability, proliferation, and differentiation in drd5-KO ↓ Colitis manifestations with D5 deficient T-cells or CCR9 deficient T-cells ↑ ERK1/2 phosphorylation and cAMP accumulation with SKF81297 treatment	AlphaScreen Time-resolved fluorescence resonance energy transfer	Osorio-Barrios et al., 2021
Mouse	Macrophage [colonic macrophages from DSS-induced colitis model (drd5-KO)]	D5	Dopamine (20 *μ*M)	↑ Severity of DSS-induced colitis in drd5-KO mice and in WT recipients following bone marrow transplant from drd5-KO mice ↑ TNF-*α*, IL-6, NF-*κ*B, and CCL2 in drd5-KO mice (blocked by dopamine) ↓ CD86, ↓ iNOS and ↑ M2-polarization markers with dopamine	ELISA FACS Immunoblotting qPCR	Liu, Wu et al., 2021
Human	Serum (from healthy controls and patients with ulcerative colitis or Crohn’s disease)	D2	N/A	DRD2 Taq1A polymorphism was associated with lower risk of development of refractory Crohn’s disease in individuals homozygous for the A_2_A_2_ allele	PCR-RFLP	Magro et al., 2006
Mouse	T-cell (CD4^+^ T-cells from layers of small intestine of DSS-induced colitis mice)	D3	PD128907 (50 nM) Dopamine (100 nM–1 *μ*M)	↓ T_h_17-mediated immunity in Drd3-KO mice ↑ IL-10 in Drd3 deficient T_regs_ ↑ CCR9 expression in Drd3 deficient T_reg_s isolated from mesenteric lymph node PD128907 limited T_reg_ ability to suppress CD4^+^ T-cell proliferation in vitro	Immunofluorescence in situ proximity ligation assay	Ugalde et al., 2021
Mouse	T-cell (mesenteric lymph node CD3^+^ T-cells from DSS-induced colitis model)	D1-like D2-like	Berberine (400 *μ*L/mL)	↓ IFN*γ* and IL-17 production with berberine	ELISA	Kawano et al., 2015
Rheumatic diseases						
Human	Synovial tissue samples and cells from patients with RA and OA	TH VMAT2	Reserpine (10 nM–1 *μ*M)	TH^+^ cells only present in inflamed tissues and not healthy controls ↓ TNF, cAMP and CREB with reserpine	Beadlyte cytokine assay	Capellino et al., 2010
Human	B lymphocyte (peripheral blood B lymphocytes from RA and OA patients)	D1 D2	N/A	↓ D2 receptor expression in both OA and RA patients compared with healthy controls ↓ D1 receptor expression in OA compared with healthy control D1-like receptor expression was negatively correlated with rheumatoid factor	Flow cytometry	Wei et al., 2015
Human	B lymphocyte (peripheral blood B lymphocytes and synovial tissues from RA and OA patients)	D2	N/A	D2 receptor negatively correlated with serum TNF*α* levels in RA patients ↑ D2 expression in RA patients compared with OA patients and healthy controls	Immulite immunoassay	Wei et al., 2016
Human	T lymphocyte (human peripheral blood CD4^+^ T lymphocytes)	D1-like	Dopamine (1 *μ*M) SCH23390 (10 *μ*M)	↑ Dopamine in RA synovial fluid ↑ IL-6-dependent IL-17 production by CD3/CD28 stimulated CD4^+^ T-cells with dopamine and SCH23390	ELISA	Nakano et al., 2011
Mouse	Macrophage (bone marrow–derived macrophages)	D1-like	SCH23390 (0.005–0.05 mg/kg) A68930 (0.05 mg/kg)	↓ Severity of collagen-induced arthritis in mice treated with SCH23390	ELISA	Nakashioya et al., 2011
Mouse	Macrophage (bone marrow–derived macrophages)	D1-like D2-like	Low dopamine (2 *μ*g/kg) High dopamine (10 *μ*g/kg) Haloperidol (10 nM)	↓ Osteoclast number in dopamine treated mice ↓ RANK-L, TNF-*α*, IL-1*β* and IL-6 in dopamine treated groups (reversed by haloperidol)	ELISA Immunohistochemistry	Yang et al., 2016
Human	Mast cell (from synovial fluid of patients with RA)	D3	N/A	D3 receptor expression negatively correlated with disease severity and lipid oxidation D3 receptor expression positively correlated with antioxidant levels in synovial fluid	Flow cytometry Thiobarbituric acid reaction	Xue, Li et al., 2018
Psoriasis and vitiligo						
Human	T lymphocyte (Peripheral blood CD3+ T lymphocytes from psoriasis patients)	D1-like	Fenoldopam (1 *μ*M) SKF38393 (100 nM)	↑ D1, D4, and D5 receptor expression with CD3/28 stimulation ↓ T-cell chemotaxis toward SDF-1/CXCL12 with fenoldopam and SKF38393 ↓ TNF-*α*, IFN-*γ*, IL-1*β*, IL-2, IL-6, CD69 with fenoldopam	Flow cytometry ELISA	Keren et al., 2019

BDNF, brain-derived neurotrophic factor; DDS, dextran sulfate sodium; FACS, fluorescence-activated cell sorting; HPLC, high-performance liquid chromatography; IHC, immunohistochemical; IKK, IkappaB kinase; iNOS, NO synthase; iPSC, induced pluripotent stem cell; IRF7, interferon regulatory factor 7; MAGI, multinuclear activation of a galactosidase indicator; OA, osteoarthritis; PCR-RFLP, polymerase chain reaction–restriction fragment length polymorphism; PHA, phytohemagglutinin; qPCR, quantitative polymerase chain reaction; RT-PCR, reverse-transcription polymerase chain reaction; TLE, temporal lobe epilepsy.

Anatomic changes in dopaminergic brain regions, the dysregulation of dopamine transmission, and aberrant dopamine receptor activity are key aspects in the development or progression of these diseases (Rangel-Barajas et al., 2015). Changes in dopaminergic activity in these diseases could drive pathogenesis by influencing immune function, as the etiology of many of these conditions is associated with changes in immune function (Block and Hong, 2005; Rogers et al., 2007; Cai et al., 2014; Yirmiya et al., 2015; Zrzavy et al., 2017; Thawkar and Kaur, 2019; Cignarella et al., 2020; Deng et al., 2020; Haque et al., 2020; Kam et al., 2020; Zhang et al., 2020; Bido et al., 2021). This section examines these interactions, highlighting the potential crosstalk between pathologic changes in dopaminergic regulation and dopamine-mediated immunomodulatory changes associated with the development of several neurologic diseases. We also discuss the impact of dopamine-altering therapeutics on these pathologic processes, as research in these areas could guide treatment recommendations, suggest specific targets for drug repurposing and development, and improve overall therapeutic efficacy.

#### 2. Parkinson’s Disease

PD is a progressive neurodegenerative condition that affects more than 6 million people globally (GBD 2016 Parkinson’s Disease Collaborators, 2018). It causes a variety of motor and nonmotor symptoms such as resting tremor, bradykinesia, rigidity, and gait and posture alterations, as well as cognitive impairment, anxiety, depression, sleep disturbances, and pain (Poewe et al., 2017; GBD 2016 Parkinson’s Disease Collaborators, 2018). Current data indicate that PD results in dysfunction and the loss of dopaminergic neurons in the SbN (Hirsch et al., 1988; Damier et al., 1999), which is likely related to the formation of *α*-synuclein protein aggregates known as Lewy bodies (Schulz-Schaeffer, 2010; Dagra et al., 2021). Elevated levels of *α*-synuclein are found in the CNS of PD patients (Tokuda et al., 2010; Irwin et al., 2012; Kouli et al., 2020) and can activate immune cells, such as microglia, macrophages, and T-cells, and astrocytes, increasing neuroinflammation, which is central to the progression of PD (Croisier et al., 2005; Thomas et al., 2007; Reynolds et al., 2008; Lee et al., 2010; Lindestam Arlehamn et al., 2020). Interestingly, recent data show that TH is upregulated in monocytes in the blood of PD patients and that TH expression in these cells is driven by TNF-*α* (Gopinath et al., 2021), suggesting that dopamine activity in peripheral myeloid cells may influence or be useful as a biomarker for PD progression.

In the CNS, microgliosis, astrogliosis and leukocyte infiltration are common histopathological findings in PD (Banati et al., 1998; Knott et al., 1999; Wilson et al., 2019; Kouli et al., 2020), and widespread microglial activation is present in PD patients (McGeer et al., 1988; Banati et al., 1998; Mirza et al., 2000; Imamura et al., 2003, 2005). In both humans and animal models, microglial activity correlates with neuronal death, starting prior to the death of dopaminergic neurons and exerting a neurotoxic effect on dopaminergic neurons that can result in neurodegeneration (Gao et al., 2002; Sugama et al., 2003, 2004;Ouchi et al., 2005; Zhang et al., 2005; Gerhard et al., 2006; Sawada et al., 2007; Kang et al., 2018). Neurotoxicity is mainly mediated by the production of ROS and the secretion of inflammatory cytokines such as TNF-*α*, IL-6 and IL-1*β*, which are elevated in the brain, cerebrospinal fluid (CSF), and serum in PD patients (Boka et al., 1994; Mogi et al., 1994; Müller et al., 1998; Imamura et al., 2005; Karpenko et al., 2018). The presence of ROS can exacerbate *α*-synuclein aggregation (Scudamore and Ciossek, 2018), and *α*-synuclein itself can activate microglia and astrocytes by binding to TLRs (Fellner et al., 2013; Kim et al., 2013; Daniele et al., 2015; Kouli et al., 2020; Sun et al., 2021) that are increased in PD brains (Shin et al., 2015; Dzamko et al., 2017; Maatouk et al., 2018; Ping et al., 2019). Increased TLR activation drives inflammation through NF-*κ*B (Mogi et al., 2007; Reynolds et al., 2008; Pranski et al., 2013) and activates the inflammasome, which has been increasingly linked to PD pathology (Gordon et al., 2018; von Herrmann et al., 2018). Notably, inhibition of the NLRP3 inflammasome, which is primarily activated in macrophages and microglia (Gold and El Khoury, 2015; Chaurasia et al., 2018; Guermonprez and Helft, 2019; Voet et al., 2019), improves disease progression by ameliorating dopaminergic neurodegeneration, striatal dopamine depletion, the formation of *α*-synuclein aggregates, and the secretion of TNF-*α*, IL-6, and IL-1*β* (Mao et al., 2017; Gordon et al., 2018; Campolo et al., 2019; Lee et al., 2019; Ou et al., 2021).

**Fig. 6:**
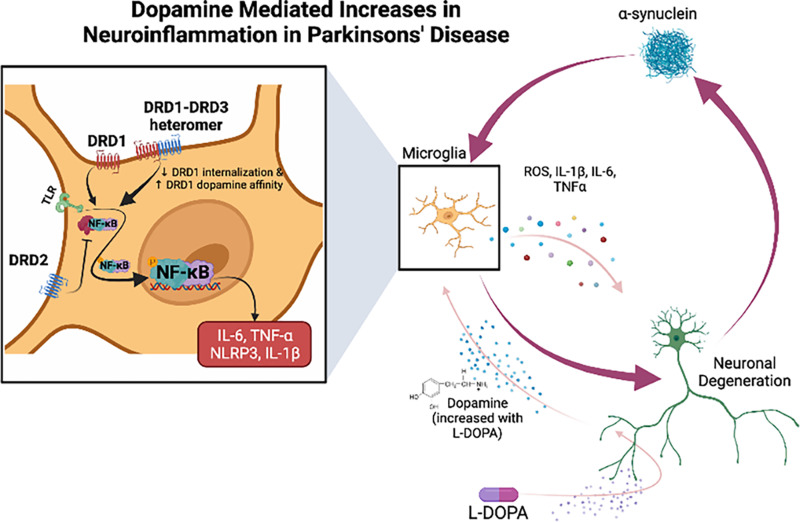
Dopamine-mediated increases in neuroinflammation in PD. The feed-forward cycle in PD starts with the disruption of the dopaminergic system that increases neuroinflammation, which, in turn, exacerbates dopaminergic neuronal dysfunction and death, further disrupting the dopaminergic system. Specifically, *α*-synuclein aggregates trigger microglial immune activation, leading to the production and secretion of neurotoxic factors (ROS, IL-1*β*, IL-6 and TNF-*α*) that result in loss of dopaminergic neurons and additional *α*-synuclein aggregation. The dysregulation of dopaminergic signaling increases neuroinflammation and feeds further into the cycle. D1/D3 receptor heteromers in PD decrease D1 internalization and increase the affinity of D1 to dopamine. Treatment with L-DOPA also increases the sensitivity of D1 to dopamine. As a result, there is excessive activation of proinflammatory D1 signaling, due to the D1/D3 receptor heteromers and L-DOPA treatment. This is concurrent with the observed downregulation of anti-inflammatory D2 signaling that can promote neuroinflammation and thus enhance neuronal degeneration in PD. Created with BioRender.com.

Disruption of the dopaminergic system that triggers immune activation, particularly activation of the NLRP3 inflammasome, may create a feed-forward cycle and exacerbate the development of PD (Fig. 6). The loss of dopaminergic neurons means these cells are no longer available to regulate the concentration or spatial distribution of dopamine via DAT-mediated uptake, potentially exposing immune cells to aberrant dopamine concentrations that could drive inflammatory activity. These effects could be compounded by changes in the expression of dopamine receptors and other dopaminergic proteins on immune cells. While D1 expression is not altered in treated or untreated PD patients (Rinne et al., 1990; Shinotoh et al., 1993; Laihinen et al., 1994), D2 expression is initially upregulated and then decreases as the disease progresses (Rinne et al., 1990, 1995; Antonini et al., 1997; Kaasinen et al., 2000; Yang, Knight et al., 2021), correlating with PD severity (Antonini et al., 1995). Downregulation of D2 occurs mainly in the striatum of PD patients and not in the SbN, where the bulk of dopaminergic cell death occurs (Yang et al., 2021b), suggesting that dopaminergic neuronal death does not directly cause the decrease in D2. Despite the lack of direct effect on neuronal viability, the age-associated reduction in D2 (Antonini et al., 1993) may be one of the reasons that age is a risk factor for PD.

The expression of D3 in PD brains is either unchanged (Yang, Knight et al., 2021) or decreased (Boileau et al., 2009) relative to healthy controls. The role of D3 in PD is not clear, but decreased Drd3 gene expression has been reported in circulating CD4^+^ T-cells from patients in the early stages of PD (Kim, Nigmatullina et al., 2019). In addition, ex vivo and in vitro studies have shown that D1 and D3 can form heteromers that decrease D1 internalization and increase the affinity of D1 for dopamine (Marcellino et al., 2008). Postmortem data show that the striatal density of both D1 and D3 in PD brain tissue is more strongly correlated with dopamine responsiveness, PD stage, age of onset, and survival time than the expression of D1 or D3 alone (Yang, Knight et al., 2021). While it is not clear how these heteromers affect D1 signaling (Fiorentini et al., 2008; Guitart et al., 2014), D1 activation can increase inflammation in human myeloid cells. Combined with the reduction in D2, the increased D1 affinity for dopamine might increase inflammatory activity mediated by this receptor and shift the dynamics of dopamine signaling toward an inflammatory state.

These effects could also change with treatment. The gold-standard treatment of PD is dopamine replacement therapy using L-DOPA, a metabolic precursor of dopamine (Fig. 2). L-DOPA restores CNS dopamine levels but does not replace the dopaminergic neurons that have died, creating an increased amount of dopamine that is not regulated as it is in healthy brains. This change in dopamine levels could further influence neuroinflammation, as highlighted by the approximately 30% of L-DOPA–treated patients that develop L-DOPA–induced dyskinesia (LID) within 5 years of treatment initiation (Tran et al., 2018). Like PD, inflammation is associated with the development of LID (Barnum et al., 2008; Bortolanza et al., 2015; Yan et al., 2021), and increases in ROS, NF-*κ*B signaling, and IL-1*β* are linked to LID progression (Barnum et al., 2008; Bortolanza et al., 2015; Yan et al., 2021). Dopamine signaling, particularly through D1, contributes to the development of LID (Rascol et al., 2001; Westin et al., 2007; Darmopil et al., 2009; Jones-Tabah et al., 2020), and L-DOPA increases D1 sensitivity, which correlates with dyskinesia severity (Aubert et al., 2005). Thus, excessive activation of D1 on microglia by L-DOPA during PD may result in neuroinflammation that contributes to the development of LID. Other dopamine receptors could also influence LID progression, as L-DOPA may decrease (Brooks et al., 1992; Thobois et al., 2004) or not effect D2 expression (Antonini et al., 1994), and D3 is upregulated in the brains of patients with LID (Payer et al., 2016). The impact of the interactions between dopamine receptors is not clear, although pharmacological inhibition of D1/D3 heteromer formation prevented the onset of LID (Fanni et al., 2019), further suggesting an important role for D1 signaling.

These data suggest dopamine receptor–mediated inflammation could be important in the development of PD and LID, and specifically that increased D1 sensitivity to dopamine induced by heteromerization with D3 and/or L-DOPA treatment is important in the progression of both diseases. Further, this pathology may be exacerbated by D2 downregulation, creating an inflammatory imbalance. However, it is important to note that the administration of nonsteroidal anti-inflammatory drugs does not reduce the risk of developing PD (Driver et al., 2011; Ton et al., 2006; Manthripragada et al., 2011; Ren et al., 2018; Poly et al., 2019; Brakedal et al., 2021), indicating that inflammation and the immunomodulatory effects of dopamine are only one aspect of disease etiology. Further defining the roles of specific dopamine receptors and their heteromers in CNS and peripheral immune function during PD could provide valuable insights and potential therapeutic targets or adjunctive therapies for the treatment of PD and LID.

#### 3. Neuropsychiatric Conditions

##### a. Schizophrenia

Schizophrenia is a debilitating chronic psychiatric disorder that is generally characterized by positive or psychotic symptoms (hallucinations and paranoid delusions), negative symptoms (decreased motivation and impaired social interaction), and cognitive deficits involving executive functioning and memory. Schizophrenia has a complex etiology but is thought to involve genetic and environmental factors that promote alterations in the dopaminergic system (Marder and Cannon, 2019; Slifstein et al., 2015). The dopamine hypothesis of schizophrenia posits that the disease is caused by an imbalance in dopaminergic transmission, with hyperactivity in mesolimbic areas such as the striatum and hippocampus and hypoactivity in the PFC (Howes and Kapur, 2009; Slifstein et al., 2015; Weinstein et al., 2017; McCutcheon et al., 2020). There may be enhanced sensitivity of postsynaptic D2 (Seeman, 2013), and subcortical D2 hyperactivity may contribute to positive symptoms, while cortical D1 hypofunction contributes to negative symptoms (Howes and Kapur, 2009; Stahl, 2017). D3 in the midbrain may also modulate negative symptoms and cognitive deficits by enhancing dopaminergic neurotransmission to the PFC and NAc (Sokoloff and Le Foll, 2017; Stahl, 2017). The role of D4 is less clear, but this receptor may impact schizophrenia by regulating GABA and glutamate transmission (Mrzljak et al., 1996; Yuen et al., 2010).

Neuroinflammation and abnormal immune responses, particular changes in the activity of T-cells, microglial cells, and peripheral monocytes, may also drive the pathogenesis of schizophrenia (Miller et al., 2011; Frydecka et al., 2018). These changes involve both inflammatory and anti-inflammatory cytokines and depend on disease duration and pharmacologic treatment (Miller et al., 2011; Müller et al., 2013), and these aberrant inflammatory processes can contribute to dopamine abnormalities (Howes et al., 2013; Purves-Tyson et al., 2017). Antipsychotic drugs, which are the standard pharmacological treatment of schizophrenia, may also directly regulate inflammation, as these drugs target dopamine receptors that are expressed on immune cells. These drugs are classified as typical or atypical, and atypical drugs are generally used at the initiation of treatment (Aringhieri et al., 2018). Older typical antipsychotics, such as chlorpromazine and haloperidol, mainly antagonize D2, while newer atypical drugs, such as clozapine and risperidone, antagonize both D2 and serotonin receptor 2A (Slifstein et al., 2015; Marder and Cannon, 2019). However, these drugs are often not specific, and many also act on other neurotransmitter receptors, including histamine, norepinephrine, GABA, and acetylcholine receptors (Li et al., 2016). There are also new drugs that preferentially target D3 over D2 (Nakajima et al., 2013), but many patients are refractory to all current treatments, and there is still considerable morbidity and mortality in this population.

**Fig. 7:**
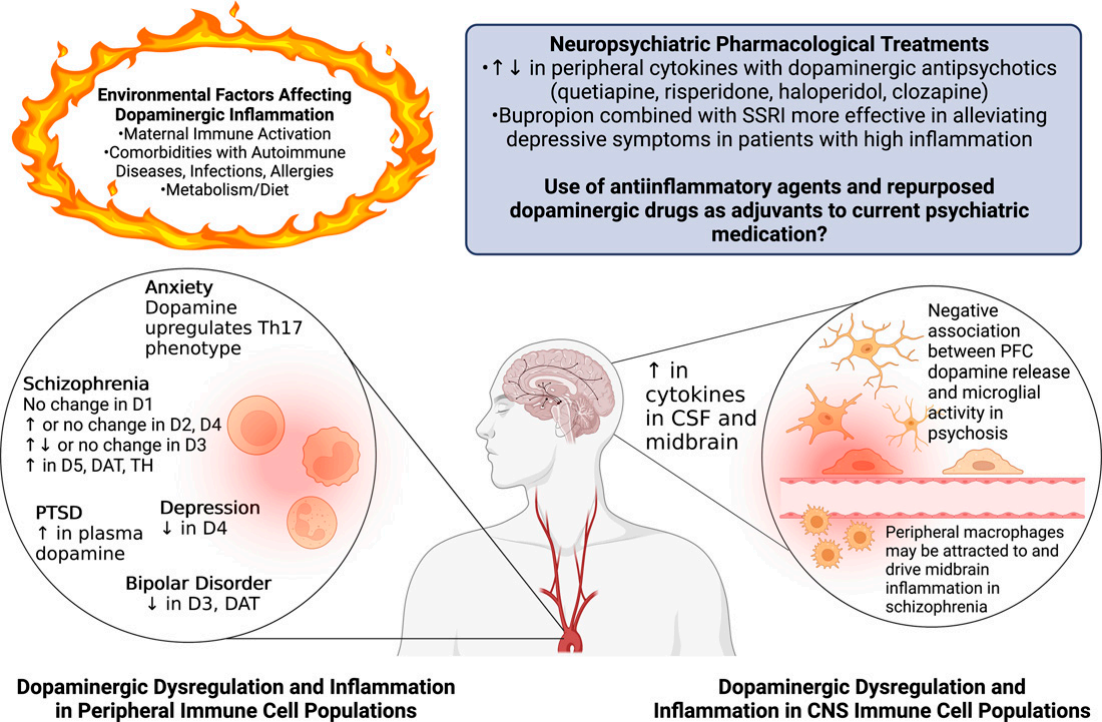
Dopaminergic inflammation in neuropsychiatric diseases. Changes in dopaminergic activity in neuropsychiatric diseases may drive pathogenesis by influencing immune function. As depicted, there are many interactions between pathologic changes in dopaminergic regulation and dopamine-mediated immunomodulatory changes associated with the development of several neuropsychiatric disorders, including schizophrenia, bipolar disorder, depression, and anxiety disorders. In peripheral immune cells such as T-cells and monocytes, alterations in the expression levels of dopamine receptors and associated dopamine proteins are observed and dependent on symptom severity and medication status. In the CNS, increases in inflammation are found in dopaminergic regions such as the midbrain, and dysregulation of CNS dopamine may drive microglial activity and, in turn, regulate peripheral immune responses. In addition, environmental factors associated with predicting neuropsychiatric disease, such as MIA, comorbidities with immune-related disorders, metabolism, and diet appear to be shared among neuropsychiatric disorders. This may contribute to and influence dopaminergic inflammation through common pathways. Finally, research regarding neuropsychiatric pharmacological treatments that are used as first-line treatments for numerous disorders suggests that these drugs, which can target the dopamine system, directly modify inflammation. Thus, they may be preferred in treating certain individuals depending on their baseline inflammatory status. More nuanced usage of novel and repurposed anti-inflammatory and/or dopaminergic drugs as adjuvant therapies could be relevant for many neuropsychiatric disorders and could provide novel targets and substantial improvements in treatments and symptomatology. Created with BioRender.com.

As both schizophrenia and its therapeutic agents target the dopaminergic system, dopaminergic changes in immune activity could drive disease progression (Fig. 7). Expression levels of dopamine receptors and associated dopaminergic proteins in peripheral immune cells from schizophrenic patients, as well as the effects of disease status and medication on these proteins, are not uniform across studies. However, the most examined receptors in peripheral immune cells are D2-like receptors. In PBMCs or PBLs, studies show no changes (Yao et al., 2008; Ahmadian et al., 2014; Cui et al., 2015) or significant increases in D2 in drug-naïve, drug-free (Zvara et al., 2005), and medicated schizophrenia patients (Wysokinski et al., 2021). Relationships between clinical symptoms, positive antipsychotic effects, and D2 expression on PBLs has also been observed (Singh et al., 2003; Brito-Melo et al., 2012), and there are differences between patients experiencing their first episode and chronic patients (Liu et al., 2013).

Studies of D3 expression have shown increases (Kwak et al., 2001; Ilani et al., 2004; Vogel et al., 2004; Boneberg et al., 2006; Fernandez-Egea et al., 2016), decreases (Vogel et al., 2004), or no differences (van der Weide et al., 2003; Zvara et al., 2005; Rodrigues et al., 2005; Urhan-Kucuk et al., 2011) in patients with schizophrenia relative to healthy controls. Negative schizophrenic symptoms and more severe psychiatric symptoms seem to correlate with higher D3 levels (Kwak et al., 2001; Vogel et al., 2004), although these discrepancies may depend on the intake time or the time needed for a positive medication effect (Kirillova et al., 2008). Antipsychotics can cause D3 expression to peak during the second week of treatment, decrease at the eighth week, and then stabilize at a level similar to that of healthy controls for up to 3 years (Kwak et al., 2001; van der Weide et al., 2003; Ilani et al., 2004; Vogel et al., 2004; Kawano et al., 2011). Most studies show no differences in D4 expression in any peripheral immune population (Ilani et al., 2004; Rodrigues et al., 2005; Kawano et al., 2011; Ahmadian et al., 2014), although one study showed an increase in Drd4 mRNA in medicated patients with schizophrenia (Wysokinski et al., 2021). There are also several clinical symptom scores that are positively and inversely correlated with D4 expression (Rodrigues et al., 2005; Boneberg et al., 2006; Kawano et al., 2011; Brito-Melo et al., 2012).

The expression of D1-like receptors and their involvement in schizophrenia is less well-studied, and D1 expression was no different in PBLs from healthy controls, treated patients, and drug-naïve patients (Ahmadian et al., 2014). However, in drug-free patients who had not taken antipsychotics for more than 3 months, Drd5 mRNA expression in PBLs increased compared with that in PBLs from medicated patients (Kwak et al., 2001). The protein ADAM metallopeptidase with thrombospondin type 1 motif 2, which is specifically associated with the activation of D1-like receptors, may be linked to key biologic mechanisms in schizophrenia and the clinical response to antipsychotics (Ruso-Julve et al., 2019). ADAM metallopeptidase with thrombospondin type 1 motif 2 is highly overexpressed in PBMCs at the onset of schizophrenia and is downregulated after 3 months of treatment. The activity of D1 may be related to negative symptoms in schizophrenia (Davidson et al., 1990; Stenkrona et al., 2019), but this has not been explored as it relates to the immune system. There is also limited research on the expression of other components of the dopaminergic system in immune cells and schizophrenia, although increases in DAT mRNA expression in lymphocytes (Liu et al., 2013) and TH expression in PBMCs (Liu et al., 2010) were seen in schizophrenic patients.

Data also show changes in the relative composition of PBMCs in patients with schizophrenia (Kelly et al., 2018), which may correlate with changes in dopamine receptor expression (Brito-Melo et al., 2012; Fernandez-Egea et al., 2016). Increased frequencies of NK cells, monocytes, B-cells, and CXCR5^+^ memory T-cells and reduced percentages of DCs, CD4^+^ memory T-cells, and HLA-DR^+^ regulatory T-cells have been found in the blood of patients who are resistant to clozapine treatment. These CD4^+^ T-cells showed significantly increased D3 expression, which correlated with a reduced frequency of T_regs_ (Fernandez-Egea et al., 2016). This finding supports studies showing that D3-signaling in CD4^+^ T-cells promotes inflammatory responses, including T_h_1- and T_h_17-mediated immunity (Franz et al., 2015; Contreras et al., 2016). Studies also show increases in D2 and D4 expression on both CD4^+^ and CD8^+^ cells in long-term hospitalized patients receiving antipsychotics (Brito-Melo et al., 2012). In addition, recent data suggest that peripheral macrophages may be attracted to the dopamine-rich midbrain and drive inflammation in the CNS in schizophrenic patients (Purves-Tyson et al., 2020). These cell type–specific changes in dopamine receptor expression could explain the lack of significance seen in previous studies that examined PBMCs as a single population and highlights the need for more cell type–focused analyses of dopamine receptor expression and function.

Inflammation-related abnormalities have been found in brain areas with dysfunctional dopaminergic signaling in individuals with schizophrenia, including the PFC, striatum, and SbN, defining distinct immune biotypes based on transcriptional profiling (Howes et al., 2013; Purves-Tyson et al., 2017; Comer et al., 2020). Data show increases in IL-6, IL-1*β*, TNF-*α*, and acute-phase protein (SERPINA3) mRNA in the schizophrenic midbrain (Purves-Tyson et al., 2020) and cerebral cortex (Fillman et al., 2013; Zhang, Catts et al., 2016), with higher levels in the midbrain. In patients on the psychosis spectrum, examining stress-induced PFC dopamine release using [11C]FLB457 PET showed a negative association between PFC dopamine release in response to acute psychosocial stress challenge and the expression of hippocampal translocator protein (TSPO) (Schifani et al., 2019), a surrogate for microglia activity (Sandiego et al., 2015). These data indicate a connection between dopaminergic signaling and neuroinflammation in schizophrenia and further suggest that stress can exacerbate inflammation. The data also show positive correlations between the dose of chlorpromazine and immune markers in the midbrains of individuals with schizophrenia, suggesting that antipsychotic treatment, as well as disease, can influence immune profiles in the brain (Cotel et al., 2015; Baumeister et al., 2016; Trepanier et al., 2016).

These hypotheses are supported by human studies examining prenatal immune activation and animal studies using the maternal immune activation (MIA) model of schizophrenia. This model shows an elevated risk of schizophrenia following prenatal exposure to maternal infection and subsequent immune-mediated disruption of early brain development (Winter et al., 2009; Meyer, 2014). Animal studies show a broad spectrum of behavioral, physiologic, and molecular alterations in the offspring of mothers exposed to inflammatory stimuli such as polyinosinic:polycytidylic acid or LPS (Meyer, 2014; Brown and Meyer, 2018). These changes are associated with both neurons and CNS immune cells (Boksa, 2010; Bergdolt and Dunaevsky, 2019) and depend on the timing of the inflammatory stimuli and the age of the offspring (Juckel et al., 2011; Mattei et al., 2014). MIA can lead to behavioral and molecular alterations in offspring relevant for the function of the dopaminergic system (Aguilar-Valles et al., 2020). For example, data show increases in striatal dopamine synthesis capacity in late-adolescent nonhuman primates (Bauman et al., 2019). Data also show increases in the levels of TH, dopamine, and dopamine-derived metabolites and decreases in D1 and D2 in the brains of adult mice and rats (Meyer et al., 2008; Oh-Nishi et al., 2022). Further, MIA offspring exhibited biphasic changes in TH and DAT, as well as deficits in prepulse inhibition behavior that was rescued by dopamine receptor antagonism (Vuillermot et al., 2010). Animals exposed to MIA show inflammatory changes in dopaminergic brain regions during adulthood, and mice showed increased expression of several midbrain immune markers that were similar to changes seen in the human midbrain (Purves-Tyson et al., 2020). MIA can even induce transgenerational effects on brain and behavior, modifying dopaminergic activity across multiple generations, possibly through DNA methylation of the dopamine-specific factor nuclear receptor-related 1 protein (Weber-Stadlbauer et al., 2021). These data suggest that interactions between dopamine and inflammation during the embryonic stage may create a bidirectional cycle of dysregulated dopaminergic transmission and immune activation that could contribute to the etiology of schizophrenia later in life and even across generations.

While the immune dysregulation associated with schizophrenia seems to be both a cause and consequence of disease progression, it is not clear whether correlations with inflammatory markers following treatment are mediated by the drugs or the psychiatric symptoms. In vivo and in vitro studies show that antipsychotics can be both inflammatory and anti-inflammatory (Himmerich et al., 2011; Krause et al., 2013; Cotel et al., 2015; Holmes et al., 2016), but studies also show that baseline inflammation predicts treatment efficacy (Mondelli et al., 2015). Anti-inflammatory treatments can partially attenuate or even fully prevent the development of schizophrenia in animal models (Mattei et al., 2014, 2017), and treatment of patients with schizophrenia with anti-inflammatory drugs in combination with antipsychotics leads to better cognitive outcomes than treatment with antipsychotics alone (Chaudhry et al., 2012; Chaves et al., 2015; Xiang et al., 2017). Many studies have used minocycline, which exerts anti-inflammatory, antimicrobial, and neuroprotective effects (Yrjanheikki et al., 1998) and has been suggested to treat schizophrenia by acting on the dopaminergic system (Du et al., 2001; Meulendyke et al., 2012). It is likely that crosstalk between the immune and dopaminergic systems could explain some of these contradictory findings, although this will require examining the specific dopamine receptors on different immune cell types and how therapeutics interact with them.

In the context of schizophrenia, it has been hypothesized that lower dopamine levels may selectively stimulate high-affinity dopamine receptors (D3 and D5), triggering inflammation, while higher dopamine levels could also stimulate low-affinity dopamine receptors (D1 and D2), inducing an anti-inflammatory effect (Pacheco, 2017; Vidal and Pacheco, 2020). Thus, in drug-free schizophrenic patients with elevated expression of high-affinity dopamine receptors (D3 and D5), dopamine induces inflammatory effects. Conversely, medicated patients show changes in dopamine receptor expression, favoring the expression of low-affinity receptors (D2) in immune cells, thus acquiring anti-inflammatory profiles in response to dopamine. A recent study indicated that patients with schizophrenia demonstrated varying dopamine receptor expression ratios (Wysokinski et al., 2021), suggesting that evaluating the totality of dopamine receptors expressed in each cell might be useful when assessing disease severity and subsequent treatment. Additionally, further evaluation of the relationship between peripheral and central dopamine receptors and the specific immunologic changes they mediate is critical, as the connections are still unclear. However, as peripheral immune cells are easily accessible in blood samples, these cells could potentially be used to monitor the status of homologous brain dopamine receptors (Buttarelli et al., 2011; Tomasik et al., 2016). They may also be useful tools with which to monitor changes in dopamine receptor expression with respect to the efficacy of therapeutic interventions (Buttarelli et al., 2011; Penedo et al., 2021). Using these types of data could enable a more nuanced usage of novel and repurposed anti-inflammatory and/or dopaminergic drugs as adjuvant therapies for schizophrenia and could provide novel targets and substantial improvements in the treatment and symptomatology involved in this disorder.

##### b. Bipolar Disorder

Bipolar disorder is a chronic, recurring psychiatric disorder characterized by mood shifts from acute depression to mania and hypomania, with intermittent periods of stable mood states (euthymia) (Müller and Leweke, 2016). Clinical and physiologic manifestations of bipolar disorder are heterogeneous and include psychomotor, metabolic, and neurovegetative alterations, but severe mood changes are the most evident sign (Benedetti et al., 2020). Bipolar disorder is linked to dysregulated dopamine signaling, and it is hypothesized that changes in dopaminergic function are associated with switches between depressive (hypodopaminergic) and manic (hyperdopaminergic) episodes in patients (Ashok et al., 2017). Inflammation and immune activation are important in the etiology and pathophysiology of bipolar disorder (Benedetti et al., 2020; Jones et al., 2021), and bipolar individuals exhibit a persistent, low-grade inflammatory state. Inflammation increases during manic episodes and decreases during depressive episodes (Modabbernia et al., 2013; Lu et al., 2019). These changes can be observed as fluctuations in peripheral cytokine levels in bipolar patients that depend on the state of mood during episodes (Ortiz-Dominguez et al., 2007; Brietzke et al., 2009). Abnormalities in the immune system have also been linked to brain region–specific activation states (Felger, 2018), symptom severity (Goldstein et al., 2009) and treatment efficacy (Goldstein et al., 2009; Benedetti et al., 2017). This suggests that dopaminergic immunomodulation could contribute to the development of this disorder (Fig. 7).

There is relatively little literature on dopamine and immune function in bipolar individuals, but studies examining patients diagnosed with schizophrenia, bipolar (Vogel et al., 2004), and psychosis (Marazziti et al., 2010) showed decreased Drd3 and DAT mRNA in PBLs. However, polymorphisms in dopamine receptors that can affect function and signaling in T-cells (Cosentino et al., 2015) are not associated with bipolar disorder (Wong et al., 2000). Postmortem examinations of the CNS of patients diagnosed with bipolar showed that DAT was decreased or unchanged (Lee et al., 2009), D2 was upregulated (Zhan et al., 2011; Kaalund et al., 2014), and D1-like receptors were upregulated (Knable et al., 2004; Pantazopoulos et al., 2004; Kaalund et al., 2014). These CNS studies did not specifically examine immune cells, and there are several confounding factors such as medication status, the method of collection, and mood state at the time of collection/death. Still, these findings suggest differences in the way that dopamine signaling affects bipolar disorder in the CNS relative to the periphery.

While bipolar disorder has not been specifically studied in animal models of MIA, some of the experimentally induced phenotypes, such as deficits in sensorimotor gating (Meyer and Feldon, 2009; Meyer, 2014) and depression-like behaviors (Khan et al., 2014; Ronovsky et al., 2017), suggest that dopaminergic dysfunction may be involved in bipolar disorder (Brown and Meyer, 2018). In a rat model with alternating mania- and depression-like behaviors, viral D1 overexpression in glutamatergic neurons in the medial PFC (mPFC) resulted in mania-like behavior, and termination of viral D1 overexpression alone was sufficient to induce depression-like behavior (Sonntag et al., 2014; Freund et al., 2016). Although the behavioral effects were relatively mild, D1 overexpression and its subsequent termination in mPFC also correlated with increased IL-6 in the hippocampus, linking the modulation of D1 expression to neuroinflammation.

Dopamine antagonists and partial agonists are widely used in the pharmacological treatment of bipolar disorder (Reynolds, 2011; Azorin and Simon, 2019), as is lithium, which also modulates the dopaminergic system (Beaulieu et al., 2004; Can et al., 2016; Carlson and Dixon, 2018). As in schizophrenia, adjunctive treatment with some anti-inflammatory agents seems to alleviate mania and improve depressive symptoms (Goldsmith et al., 2016; Rosenblat, 2019). Notably, lumateperone, an antagonist for D1, D2, D4, and serotonin receptor 2A and transporters (Kumar et al., 2018), improved depressive symptoms in bipolar depression patients in the first clinical trials (Calabrese et al., 2021), although it also showed potential inflammatory effects (El-Haroun et al., 2021). Overall, these data suggest a relatively understudied connection between dopaminergic modulation of immune function and bipolar disorder and indicate that the combination of anti-inflammatory and dopaminergic drugs could provide more beneficial alleviation of bipolar symptomatology than individual treatment.

##### c. Depression

Depression is a psychiatric disorder characterized by low mood, anhedonia, feelings of guilt or low self-worth, disturbed sleep or appetite, low energy, and suicidal ideation. It is a major cause of disability worldwide with over 322 million people living with depression (World Health Organization, 2017). Standard pharmacological treatments for depression include antidepressants that predominantly act on serotonin but also other monoamines. These include selective serotonin reuptake inhibitors (SSRIs), tricyclic antidepressants, and monoamine oxidase inhibitors, as well as typical and atypical antipsychotics for more treatment-resistant patients. Antidepressant therapies are effective for many patients with major depression, especially when paired with evidence-based psychotherapy such as cognitive-behavior therapy. However, even with initial treatment, over two-thirds of patients continue to have significant depressive symptoms (Rush et al., 2006; Trivedi et al., 2006). Further, there are no clinical variables that identify subgroups of patients who respond differently to the currently available antidepressants (Arnow et al., 2015; Zeier et al., 2018).

There are many hypotheses regarding the etiology of depression, including hyperactivation of the HPA axis (Horowitz et al., 2013), nutritional deficiencies (Rao et al., 2008), and imbalances in neurotransmitters (serotonin, norepinephrine, dopamine, and glutamate) that contribute to neural circuit dysfunction (Delgado, 2000; Dunlop and Nemeroff, 2007; Belujon and Grace, 2017). Data also indicate that increased inflammation in both the CNS and periphery is a critical contributor to and target for treatment in depression (Miller and Raison, 2016; Wohleb et al., 2016; Kaufmann et al., 2017; Beydoun et al., 2020). Inflammatory cytokines and acute phase proteins are increased in depressed patients, with consistent increases in IL-6, TNF-*α*, and C-reactive protein (CRP) in blood and CSF (Howren et al., 2009; Miller et al., 2009; Dowlati et al., 2010; Kohler et al., 2017). Further, low-grade inflammation associated with aging, chronic infections such as HIV, and autoimmune diseases such as MS and RA are all risk factors for developing depression, and patients with depression with increased inflammation may represent a relatively treatment-resistant population with increased risk for SUDs and suicide (Alexopoulos and Morimoto, 2011; Raison et al., 2013; Brundin et al., 2017; Pryce and Fontana, 2017; Furman et al., 2019).

It is increasingly appreciated that depressive-like behaviors associated with infection and the related inflammatory response may be direct consequences of the impact of inflammatory cytokines on dopamine signaling. Both animal and human systems have examined the relationships between dopamine, inflammation, and depression, showing that immune activation, specifically production of inflammatory cytokines, can disrupt activity in dopaminergic brains regions and dopamine neurotransmission. This includes dysregulation of dopamine synthesis, release, and reuptake, which are associated with depressive symptoms such as disrupted reward-seeking behavior, motivation, and psychomotor slowing (Felger et al., 2007; Brydon et al., 2008; Capuron et al., 2012; Felger et al., 2016; Felger and Treadway, 2017). Studies have shown specifically that D3 modulates LPS-induced depressive-like behaviors and that pretreatment with pramipexole, a preferential D3 agonist, showed antidepressant effects on these behaviors. These effects were driven by preventing changes in the expression LPS-induced inflammatory cytokines, brain-derived neurotrophic factor, and ERK1/2-CREB signaling pathway components in the VTA and NAc. Conversely, treatment with the D3 selective antagonist NGB 2904 alone rendered mice susceptible to depression-like effects and caused changes similar to the LPS-induced alterations in the mPFC and NAc (Wang, Xu et al., 2018). Another study showed that chronic social stress, which is a major risk factor for depression, precipitated immune activation in the periphery and dopaminergic regions of the CNS, and reduced dopamine-dependent reward-directed behavior. In the presence of the DAT inhibitor GBR12909, chronically stressed mice also exhibited less reward-directed behavior and attenuation of dopamine-mediated stimulatory effects on locomotor activity and NAc activation (Bergamini et al., 2018). Outside the CNS, changes in insulin levels can affect CNS dopaminergic signaling, and insulin resistance may further contribute to dopamine-mediated inflammation and depressive symptoms (Kleinridders et al., 2015; Stouffer et al., 2015; Kleinridders and Pothos, 2019). In the gut microbiome, alterations in patients with depression (Cheung et al., 2019; Zheng et al., 2016) could contribute to dysregulated inflammatory responses through changes in dopamine levels, as patients with depression are deficient in several species of gut bacteria (Valles-Colomer et al., 2019), such as *Coprococcus*, which are associated with dopamine pathway activity.

Immunometabolic changes could also drive the connection between dopamine and depression, as a subset of depressed patients with high CRP levels and anhedonia showed increased glucose and low tyrosine metabolism. The authors of one study posit that the anhedonic state results from immunometabolic shifts away from dopamine synthesis pathways that lead to reduced dopamine precursor availability (Bekhbat et al., 2020). Thus, the interactions between inflammation and metabolism on dopaminergic reward pathways may represent one pathophysiologic mechanism of depressive symptoms such as anhedonia (Swardfager et al., 2016; Pan, Rosenblat et al., 2017). This correlates with a recent conceptual framework hypothesizing that the immunometabolic demands of chronic low-grade inflammation could induce a transient reduction in CNS dopamine to shift effort-discounting behavior (Treadway et al., 2019). This suggests communication between the immune system and dopamine is crucial in shaping effort allocation as a function of peripheral immunometabolic states (aging, obesity, etc.). Further, this shows how the dopaminergic response to increased immunometabolic demands during chronic inflammation could contribute to motivational impairments in psychiatric disorders such as depression (Treadway et al., 2019; Lucido et al., 2021).

Despite a limited understanding of the specific inflammatory processes and comorbidities driving depression, the importance of inflammation is supported by the well-documented immunomodulatory effects of antidepressants. In vivo, significant decreases in peripheral inflammatory markers have been found after initiation of antidepressant treatment (Hannestad et al., 2011; Hiles et al., 2012; Cattaneo et al., 2013; Alcocer-Gomez et al., 2014; Dahl et al., 2014; Strawbridge et al., 2015). Reductions in inflammatory cytokines and myeloid activation has also been observed after antidepressant treatment in vitro in primary human peripheral immune cells (Xia et al., 1996; Maes et al., 1999; Diamond et al., 2006; Munzer et al., 2013; Nazimek et al., 2017). The in vivo effects on inflammation may result from both the direct impact of the bioactive drug on immune cells and changes in the concentration of neurotransmitters to which these cells are exposed, including dopamine (Fig. 7). Supporting this hypothesis, PBMCs from patients with untreated depression had significantly lower levels of Drd4 mRNA than healthy control subjects. Further, treatment with the SSRI paroxetine not only improved depressive symptoms but also increased levels of Drd4 mRNA in their PBMCs (Rocc et al., 2002). Thus, the therapeutic effect of SSRIs may be mediated, at least in part, through an increase in dopamine receptor sensitivity in these cells, which may modulate inflammation.

Patients with depression with higher baseline inflammation also respond more poorly to serotonergic antidepressants such as SSRIs compared with nonserotonergic antidepressants that modulate dopamine neurotransmission, such as nortriptyline and bupropion (Uher et al., 2014; Jha et al., 2017). Higher baseline levels of IL-17 were associated with greater reductions in depression severity in response to combined bupropion-SSRI treatment but not SSRI monotherapy or the combination of venlafaxine and mirtazapine (Jha et al., 2017). In a case series of patients with treatment-resistant depression, pramipexole (D3 agonist) treatment was particularly effective in patients with higher levels of IL-17 (Fawcett et al., 2016). These studies suggest that the biologic mechanism underlying the differential improvements in patients with elevated IL-17 may be related to dopaminergic modulation of CNS inflammation, either directly or through modulation of dopamine synthesis and neurotransmission. Thus, drugs that modulate dopaminergic neurotransmission may be preferred in treating individuals with depression with high inflammation. However, stimulant medications that increase dopamine release and/or block dopamine reuptake have shown limited efficacy in the treatment of depressive symptoms in patients with inflammation-associated medical illnesses (Mar Fan et al., 2008; Hardy, 2009; Moraska et al., 2010). This could be due to the inflammatory impact of increased dopamine concentrations, as discussed in the prior section. Considered alongside data showing inflammation can affect dopamine function in the context of depression, this indicates a need to explore new antidepressant strategies focused on inflammatory and/or dopaminergic signaling, including compounds that modulate dopamine synthesis, packaging, or receptor signaling.

One example of this is tetrahydrobiopterin (BH4), an essential enzyme cofactor that is required to produce tyrosine and dopamine, that has been discussed as a potential treatment target (Sperner-Unterweger et al., 2014). In acute inflammation, dopamine synthesis can be stimulated by the upregulation of BH4 production, while chronic inflammation leads to oxidative loss of BH4 and consequent reduction in dopamine synthesis (Fanet et al., 2021). There are several compounds that can boost BH4 availability or activity, which may facilitate dopamine synthesis. The administration of BH4 itself, as well as folic acid, L-methylfolate, or S-adenosyl-methionine, all of which have roles in the synthesis and/or regeneration of BH4 (Shintaku, 2002; Stahl, 2007), have demonstrated efficacy as adjuvants to antidepressants (Papakostas et al., 2010; Ginsberg et al., 2011). Other alternative therapeutic targets could be inhibition of the indoleamine 2,3-dioxygenase pathway or glutamate, which both can modulate dopamine release (Rassoulpour et al., 2005; Hutchison et al., 2018). The indoleamine 2,3-dioxygenase antagonist 1-methyl tryptophan can mitigate the impact of inflammatory stimuli such as LPS on depressive-like behavior (O’Connor, Lawson, André, Briley et al., 2009, O’Connor, Lawson, André, Moreau et al., 2009). The administration of glutamate receptor antagonists, such as the N-methyl-D-aspartate antagonist ketamine, has potent antidepressant effects in patients with treatment-resistant depression who exhibit increased inflammation (aan het Rot et al., 2010; Chen et al., 2018). Therefore, alternative strategies such as augmenting BH4 activity, blocking the kynurenine pathway, or modulating glutamate neurotransmission may be beneficial in restoring dopamine function and treating depression in patients with increased inflammation (Felger et al., 2016).

Finally, strategies that directly inhibit the inflammatory effects of dopamine could also be considered. Inhibiting inflammatory cytokines, such as TNF-*α*, has been shown to reduce depressive symptoms including anhedonia and psychomotor slowing in patients with inflammatory disorders and in depressed patients with increased inflammation (Tyring et al., 2006; Raison et al., 2013), although it is unclear whether the improvement is due, at least in part, to their effects on the comorbid inflammatory diseases. Recently, improvement in anhedonia in response to TNF-*α* inhibition with infliximab in depressed patients with high inflammation was associated with a reduction in CD14^+^ monocyte and effector memory T-cell populations (Bekhbat et al., 2022). This suggests that multiple distinct immune cell types appear to participate in the chronic inflammatory response and subsequent behavioral changes found in patients with depression. Therefore, specific immunotherapies that target cytokines that activate specific immune cell subpopulations (Rider et al., 2016; Hu et al., 2019) could have greater efficacy and safety than available anti-inflammatory therapies such as infliximab. As dopamine has varying effects on different immune cell populations, investigation of cell type–specific regulation of dopamine’s effects on inflammation with these targeted drugs is warranted and could lead to novel therapeutics for depression. Further, NLRP3 activation, which we and others have shown to be affected by dopamine, is implicated in depressive behaviors, and can be modulated by current antidepressant therapies (Alcocer-Gomez et al., 2014, 2017; Wong et al., 2016; Syed et al., 2018). This finding suggests that dopaminergic strategies could act on this mechanism and play roles in the antidepressant response, and drugs that modify the inflammasome could also be considered as novel therapeutic agents for depression.

##### d. Anxiety Disorders

Along with depression, anxiety disorders are among the most common psychiatric diseases, affecting 264 million people globally (World Health Organization, 2017). Anxiety is a complex feeling of uneasiness, fear, and worry, and in individuals with anxiety disorders, anxiety is more frequent, intense, and persistent. These disorders include but are not limited to generalized anxiety disorder (GAD), social anxiety disorder (SAD), post-traumatic stress disorder (PTSD), panic disorder, and phobias. First-line treatment of anxiety disorders consists of SSRIs, serotonin, norepinephrine reuptake inhibitors (SNRIs), and pregabalin, with benzodiazepines such as diazepam as second-line options. Many patients do not respond to first-line treatments, or they continue to have residual symptoms, putting them at high risk of experiencing disorder chronicity and a lower quality of life. New and repurposed pharmacological agents, such as mood stabilizers and atypical antipsychotics (especially quetiapine), have been introduced as monotherapies or combined with SSRIs/SNRIs, but these treatments are not recommended as first-line options (Maron and Nutt, 2017).

Anxiety research has been somewhat confounded by its high comorbidity with depression and SUDs. However, data show that dopaminergic signaling plays a critical role in the regulation of anxiety behaviors (Zweifel et al., 2011; Falco et al., 2014; Zarrindast and Khakpai, 2015), and both D1- and D2-like receptors, as well as DAT, can mediate these effects (de la Mora et al., 2010; Bananej et al., 2012; Perez de la Mora et al., 2012). Different genetic predispositions to dopaminergic reactivity may contribute to the occurrence of anxiety disorders, such as PTSD (Segman et al., 2002), suggesting that variations in dopaminergic neurotransmission may mediate the pathologic response to trauma and general vulnerability to the effects of stress. Further, dopaminergic dysfunction may be more pronounced in some anxiety disorders than in others. Unlike in other anxiety disorders, in GAD, serotonin reuptake site density is unchanged (Maron et al., 2004) while dopamine reuptake site density in the striatum is lower in GAD patients than healthy controls (Lee, Tsai et al., 2015). A PET study showed increased serotonin transporter/DAT coexpression in SAD patients in the amygdala and NAc, and SAD diagnosis was predicted by the interaction between serotonin transporter and DAT availability (Hjorth et al., 2021). These data suggest that interactions between dopamine and other monoamines causes dysregulation that may underlie the differences in anxiety disorder symptomology.

While depression is frequently associated with immune dysregulation, there is much less research on the relationship between anxiety disorders and inflammation. A few studies have correlated anxiety symptoms with increased cytokine levels, including CRP and IL-6 (Pitsavos et al., 2006; Liukkonen et al., 2011). Evidence from clinical studies suggests increased inflammatory activation in patients with panic disorder (Hoge et al., 2009) and GAD (Bankier et al., 2008) and decreased inflammatory activation in patients with social phobia (Vogelzangs et al., 2013). Research has mainly focused on PTSD, in which high levels of inflammatory markers have been found (Gill et al., 2009; Spitzer et al., 2010). These markers have been correlated with PTSD symptom levels (von Kanel et al., 2007), and both psychiatric medication and comorbid depression were important moderators of symptomology (Passos et al., 2015). Peripheral immune activation in PTSD was associated with deficient brain microglial activation as measured by prefrontal-limbic TSPO availability. TPSO availability was negatively associated with PTSD symptom severity and was decreased relative to controls (Bhatt et al., 2020). Additionally, higher CRP levels were associated with lower TSPO availability and higher PTSD severity. Interestingly, greater changes in IL-6 after LPS administration in humans significantly and independently predicted a more pronounced LPS-induced anxiety response (Lasselin et al., 2016). Further, higher pre-existing subclinical anxiety symptoms significantly predicted a lower increase in anxiety after LPS administration, which was mediated by TNF-*α* (Lasselin et al., 2016). Significant immune dysregulation has also been found in individuals with late-onset anxiety disorder, suggesting the presence of a specific late-onset anxiety subtype with a distinct etiology that could benefit from more personalized treatment strategies (Vogelzangs et al., 2013).

There has been little research on immune dysregulation and anxiety, which has predominantly focused on acute and chronic stress and HPA axis dysregulation, but limited evidence suggests that dopamine plays a role. In rodents, modafinil, a wake-promoting drug that mediates dopamine-related psychostimulant action, prevented LPS-induced anxiety-like and depressive-like behaviors, as well as the LPS-induced increase in CD11b^+^CD45^high^ cells and IL-1*β* gene expression in the brain (Zager et al., 2018). Blocking D1-like receptors with SCH-23390 specifically counteracted the effect of Modafinil on anxiety-like behaviors, suggesting the effects were mediated via D1-like receptor activity. Human studies show that peripheral dopamine levels, including plasma and urinary dopamine, are altered in anxiety disorders and correlate with symptom severity (Yehuda et al., 1992; Hamner and Diamond, 1993; Glover et al., 2003; LeBlanc and Ducharme, 2007). Platelet MAO-B activity, which modulates dopamine metabolism (Fig. 2), was higher in veterans with psychotic PTSD than healthy individuals or veterans without PTSD (Pivac et al., 2007). In individuals with GAD, T-cells proliferated less following mitogen-induced T-cell activation, while dopamine reduced the proliferative response of phytohemagglutinin-activated T-cells from healthy subjects but not those from GAD individuals. The cytokine profiles of GAD individuals revealed T_h_1 and T_h_2 deficiencies associated with a dominant T_h_17 phenotype, which was enhanced by dopamine (Ferreira et al., 2011). In trauma-exposed women, dysfunction in corticostriatal reward circuitry correlated with inflammation in association with anhedonia symptoms and PTSD (Mehta et al., 2020). Together, these data indicate that inflammation contributes to anxiety disorders and that dopamine contributes to this process.

It is difficult to clearly extrapolate the mechanisms associated with dopamine and inflammation in anxiety disorders, as many studies on dopamine or inflammation alone are inconsistent and confounded due to overlapping disorder etiologies. Whether an anxiety subgroup is defined by the type of disorder, the severity or duration of the disorder, the comorbidity with depression, or age of onset has yet to be examined. Further research is needed to distinguish the roles of dopamine and inflammation in anxiety from other mental disorders and to provide new biologic insights into anxiety pathogenesis and treatment. As first-line treatments mainly include antidepressants and antipsychotics, and other dopamine modulating drugs are indicated, the potential interactions and strategies for treating depression and schizophrenia (discussed in previous text) may be relevant and should be considered.

#### 4. Neurodevelopmental Diseases

##### a. ADHD

ADHD is a neurologic disorder that typically manifests early in development and is characterized by a persistent pattern of inattention, disorganization, and/or hyperactivity-impulsivity. While ADHD is usually thought of as a childhood disorder, it is now considered a chronic condition. Around 50% of children with ADHD have continued impairment into adolescence, with 30% to 60% of those continuing into adulthood (Salmeron, 2009), substantially impacting quality of life (Danckaerts et al., 2010). Historically, ADHD was considered a hypodopaminergic disorder (Levy, 1991; Genro et al., 2010) in which synaptic dopamine deficiency leads to clinical symptoms. Imaging studies indicate reduced volumes in dopaminergic brain regions such as the basal ganglia, caudate, and putamen (Nakao et al., 2011; Hart et al., 2013; Roman-Urrestarazu et al., 2016; Hoogman et al., 2017) and polymorphisms in genes encoding DAT, D4, D5, and forkhead box protein P2, which regulates dopamine and neurodevelopment, are associated with the development of ADHD (Demontis et al., 2019; Faraone and Larsson, 2019). The role of dopamine in ADHD is also supported by the effectiveness of pharmacological interventions such as methylphenidate and amphetamines that increase dopamine levels in the synaptic cleft (Volkow, Wang et al., 2009). These stimulants directly target the dopaminergic system and are the current first-line pharmacological treatments for ADHD, in addition to nonstimulants (atomoxetine, guanfacine, and clonidine) that also modify dopamine levels (Murai et al., 1998; Bymaster et al., 2002; Arnsten and Pliszka, 2011). Although drugs that target dopaminergic pathways are the first-line treatments for ADHD and their short-term effectiveness has been widely demonstrated (Posner et al., 2020), up to 30% of ADHD patients do not respond to these treatments (Kolar et al., 2008).

Recent research shows a clear role for inflammation in ADHD pathophysiology (Anand et al., 2017; Dunn et al., 2019). ADHD is highly comorbid with inflammatory and autoimmune disorders such as asthma, RA, and type 1 diabetes (Chen et al., 2017; Instanes et al., 2018) and changes in CSF and peripheral cytokines are associated with ADHD in children and adults (Oades et al., 2010; Darwish et al., 2019). As in other neurologic disorders (schizophrenia, bipolar disorder), early-life exposure to environmental risk factors may increase the risk of ADHD via inflammation (Leffa et al., 2018), and animal models of MIA demonstrate behavioral and neural outcomes that are consistent with ADHD. The environmental insults associated with ADHD appear to be shared among other neuropsychiatric disorders, suggesting that perinatal insults may act through a common inflammatory pathway, which is consistent with high comorbidity with neuropsychiatric disorders (Katzman et al., 2017).

Accumulating evidence from human studies and animal models supports a role for crosstalk between dopamine and inflammation in ADHD pathophysiology. In humans, increased levels of antibodies against the basal ganglia (Toto et al., 2015) and DAT (Giana et al., 2015) have been detected in individuals with ADHD. Decreased D1 expression in the anterior cingulate cortex of ADHD individuals was associated with severe hyperactivity, activated microglia in the dorsolateral prefrontal and the orbitofrontal cortices, and deficits in attentional ability and processing speed (Yokokura et al., 2021). In rodents deficient in nuclear receptor-related 1 protein, a transcription factor essential for the development of dopaminergic neurons and implicated in ADHD, exposure to MIA induced behavioral impairments in sustained attention and attentional shifting, as well as additive effects on spontaneous locomotor hyperactivity (Vuillermot et al., 2012). Another study showed that this behavioral phenotype was ameliorated by treating MIA mothers with nonsteroidal anti-inflammatory drugs and was associated with alterations in D1 and D2 (Bronson and Bale, 2014).

Dopamine-mediated changes in the choroid plexus may link the peripheral and CNS immune systems in a DAT-deficient mouse ADHD model (Mereu et al., 2017; Castellani et al., 2019). In mice with DAT hypofunction, there were increased T_regs_ in the thymus and spleen, reduced cyclo-oxygenase 2, TNF-*α*, and Irf7 expression in microglia, and decreased monocyte infiltration into the brain. DAT hypofunction was associated with weaker choroid plexus activation, which was attributed to increased expression of I*κ*B*α* and reduced nuclear translocation of NF-*κ*B p65, and was associated with decreased mRNA expression of proinflammatory markers in the choroid plexus. Amphetamine reversed and stimulated p65 nuclear translocation in the choroid plexus in DAT-deficient mice. This study highlights the potential importance of the choroid plexus in neurologic disorders (Ghersi-Egea et al., 2018). This region expresses dopamine receptors (Boyson et al., 1986; Mignini et al., 2000), and it has been suggested that dopamine could modulate blood flow in the choroid plexus (Townsend et al., 1984). Thus, dopaminergic immunomodulation of choroid plexus function could serve as a promising target for treating neurodevelopmental disorders such as ADHD, as well as psychiatric disorders, and should be explored in more detail. For example, the direct effects of dopamine on choroid plexus-associated macrophages, known as epiplexus cells, should be examined (Meeker et al., 2012; Kaur et al., 2016).

Research also suggests that stimulants can exacerbate inflammation associated with ADHD, supporting other work on the inflammatory effects of stimulant-increased dopamine levels. In rodents, chronic oral exposure to clinically relevant, high-dose methylphenidate results in microglial activation and neuroinflammation in the cerebral cortex, hippocampus, thalamus, and basal ganglia (Carias et al., 2018) and blood–brain barrier hyperpermeability (Coelho-Santos et al., 2019). Likewise, dexamphetamine induces neuroinflammation in rodents (Thomas and Kuhn, 2005; Valvassori et al., 2019). Children taking current ADHD medications had higher plasma levels of the inflammatory adhesion molecules soluble intercellular adhesion molecule-1 and soluble vascular cell adhesion molecule-1 than children without ADHD medication, suggesting that increased inflammation is a consequence of medication (Yang et al., 2020). PET imaging using the dopamine D2 antagonist tracer ^11^C-raclopride showed that methylphenidate-induced D2 activation in the striatum was significantly higher after LPS administration than placebo (Petrulli et al., 2017).

Several repurposed non-stimulant drugs with dopaminergic properties have been investigated for treating ADHD (Pozzi et al., 2020) and compared with either methylphenidate or placebo. In many cases, these drugs are as efficacious as methylphenidate and/or more effective than placebo in children and adults. These include bupropion (Jafarinia et al., 2012; Hamedi et al., 2014), dasotraline (Koblan et al., 2015; Findling et al., 2019), centanafadine (Wigal et al., 2020), and venlafaxine (Zarinara et al., 2010; Amiri et al., 2012). However, all of these compounds also show inflammatory effects (Vollmar et al., 2008; Hajhashemi and Khanjani, 2014; Zhang et al., 2019), suggesting that the common effect on dopamine could be involved in inflammation. Alternatively, treatment of ADHD involves use of drugs that target serotonin, such as SSRIs, SNRIs, and tricyclic antidepressants (Park et al., 2014). However, even alterations in serotonin can modulate the dopaminergic system, as serotonergic terminals can take up exogenous L-DOPA and convert it to dopamine (Stansley and Yamamoto, 2013), and several serotonin receptor subtypes also influence dopamine transmission in the mesolimbic pathway (Yan and Yan, 2001; Valentini et al., 2013). Of note, coexposure to stimulant medication for ADHD and SSRI antidepressants is quite common; it is prescribed in the treatment of ADHD/depression comorbidity, which occurs in up to 40% of pediatric ADHD cases (Spencer, 2006), used as augmentation therapy in depression despite limited efficacy (Nelson, 2007; Ishii et al., 2008), or occurs via accidental coexposure in patients on antidepressants who use stimulants recreationally or as cognitive enhancers (Benson et al., 2015). A growing body of research suggests methylphenidate/SSRI combinations may potentiate addiction-related signaling and subsequent behaviors (Van Waes et al., 2010; Warren et al., 2011; Moon et al., 2021). Many additional neurotransmitter systems, including glutamate and GABA (Whitton, 1997; Lopes et al., 2019), may also interact in complex ways that may coalesce on a common pathway to cause aberrant dopamine transmission in ADHD, and this should be considered when determining optimal treatments of not only ADHD but also common neurologic comorbidities.

##### b. Epilepsy

Epilepsy is a multifaceted, chronic neurologic disease characterized by a predisposition for spontaneous and recurrent bursts of neuronal hyperactivity known as seizures. Seizures may remain confined to one brain region of origin (focal or partial seizures) or spread to entire cerebral hemispheres (generalized seizures). The development of epilepsy, epileptogenesis, derives from a diverse array of factors, including genetic predisposition, developmental dysfunction, and neurologic insult, which contribute to synaptic changes and hyperexcitable neurotransmission (Rakhade and Jensen, 2009). Seizures have been traditionally characterized as an imbalance in excitatory (glutamatergic) and inhibitory (GABAergic) transmission (McNamara et al., 2006; Ben-Ari et al., 2012). Despite the efficacy of current antiepileptic drugs, almost 30% of patients with epilepsy are refractory to medical treatment, have progressive cognitive impairment, and require neurosurgical resection of the epileptic tissue to ameliorate recurring seizures (Laxer et al., 2014). Current antiepileptic drugs mainly address symptoms, blocking seizures but not affecting the underlying pathology or the progression of the disorder (Rogawski and Loscher, 2004), thus highlighting a critical need to develop new therapeutics and strategies to prevent epileptogenesis in at-risk individuals.

While clinical and experimental studies implicate many neurotransmitters in epileptogenesis, the dopaminergic system plays a prominent role in the modulation of seizures. Drugs that increase dopamine, such as apomorphine, amphetamines, L-DOPA, and anti-parkinsonian drugs (pergolide and bromocriptine), have antiepileptic and anticonvulsant effects. Seizures involving the limbic system are most affected by modulating dopaminergic signaling (Starr, 1996; Clinckers et al., 2005). Seizure-modulating effects also depend on the dopamine receptor subtypes involved and the brain regions in which they are activated. Studies in a wide variety of animal models and humans show the opposing actions of D1- and D2-like receptor signaling in limbic epileptogenesis. Signaling through D1-like receptors generally supports epileptogenesis by reducing seizure threshold and increasing severity (Starr and Starr, 1993; Gangarossa et al., 2014). In addition, seizures are absent or significantly reduced in Drd1 or Drd5 knockout mice treated with the D1-like agonist SKF83822 (O'Sullivan et al., 2008). In humans, the neocortex of patients with temporal lobe epilepsy has high D1 expression, and D1 binding is positively correlated with epilepsy duration (Rocha et al., 2012).

In contrast, D2-like receptor signaling is generally considered to have antiepileptogenic effects, and antagonizing D2-like receptor signaling reduces seizure thresholds (de Toffol et al., 2018). Patients with temporal lobe epilepsy showed reduced D2/D3 binding in extrastriatal and striatal regions (Bernedo Paredes et al., 2015), as did patients with juvenile myoclonic epilepsy in the bilateral posterior putamen (Landvogt et al., 2010). Reduced expression of D2 receptors in epileptogenic regions is also found in different animal models of epilepsy. Moreover, pharmacological or genetic inactivation of D2 increases seizure susceptibility (Bozzi and Borrelli, 2013; Svob Strac et al., 2016). It is hypothesized that stimulation of D1-like receptors and blockade of D2-like receptor signaling activates neuronal cell death pathways involving the PKA/ERK/Fos/Jun pathway and the mammalian target of rapamycin pathway, causal factors of limbic epileptogenesis (Bozzi and Borrelli, 2013; Henshall and Engel, 2013). Most studies demonstrating dopaminergic modulation of seizure onset and spread were performed on animal models of acute but not chronic seizures, although some studies showing dopamine–seizure connections used pharmacological or limbic kindling models of epileptogenesis, which do lead to chronic seizures (Starr, 1996; Morimoto et al., 2004). Still, because dopamine and dopamine metabolite concentrations vary depending on the type of epilepsy and animal model, it is difficult to draw concrete conclusions about dopamine receptor activity.

Inflammatory processes arising from neuronal damage, gliosis, and microgliosis are also a likely common mechanism in the pathophysiology of seizures and epilepsy (Morin-Brureau et al., 2018; Rana and Musto, 2018; Vezzani et al., 2019). Steroids and other anti-inflammatory treatments exhibited anticonvulsant effects in some drug-resistant epilepsies (Riikonen, 2004; Wirrell et al., 2005), and febrile seizures are often caused by an increase in inflammation (Dube et al., 2007; Choy et al., 2014). There is a high incidence of seizures in autoimmune diseases (Geis et al., 2019) and in several clinical trials, PET imaging showing increased TSPO expression linked seizures in temporal lobe epilepsy, frontal lobe epilepsy, and focal cortical dysplasia to neuroinflammation (Gershen et al., 2015; Butler et al., 2016; Dickstein et al., 2019). Models of systemic or CNS infections suggest pre-existing brain inflammation increases seizure predisposition. This is associated with alterations in neuronal excitability and enhanced seizure-induced neuropathology, and recurrent seizures perpetuate chronic inflammation (Sayyah et al., 2003; Ravizza et al., 2008). Further, seizure activity can also induce neuroinflammation, although seizure-associated cell loss can contribute to but is not a prerequisite for neuroinflammation (Turrin and Rivest, 2004; Ravizza et al., 2008).

Most studies examining inflammation in epilepsy focus on neuroinflammatory pathways, but peripheral immune cells, and the effects of dopamine on those cells, may also contribute to epilepsy pathophysiology (Fabene et al., 2013; Cerri et al., 2016). Seizures that occur in epilepsy may increase circulating dopamine levels (Nass et al., 2019), which may modulate leukocyte release of inflammatory mediators, which could be a regulatory mechanism of neuroimmune pathways in epilepsy. Increased expression of D1, D2, D4, and D5 were found on monocytes and granulocytes of patients with temporal lobe epilepsy, while D2 expression was increased in lymphocytes (Vieira et al., 2021). Additionally, higher seizure frequency was associated with lower D5 expression on lymphocytes. The biologic significance of these data needs further examination, but these findings support our prior statements on the importance of immune cell phenotyping and the role of peripheral dopamine biomarkers in defining the mechanisms and/or prognosis of neuropsychiatric/neurodevelopmental disorders.

Currently, dopaminergic drugs are not used to treat epilepsy, but antiepileptic effects of dopaminergic agents have been reported in epileptic patients (Starr, 1996). Bromocriptine (D2-like receptor agonist) has been examined to treat some forms of epilepsy (Saie and Sills, 2005), lisuride (D2-like receptor agonist) may reduce seizures after traumatic brain injury (Zweckberger et al., 2010), and various D2-like receptor agonists have neuroprotective effects against kainic acid–induced brain damage (Micale et al., 2006). The primacy of D2-like receptor stimulation in these effects correlates with the D1/D2 dichotomy in seizure regulation and warrants further investigation of the antiepileptic efficacy of dopaminergic agents. It also suggests that seizures might be a consequence of treating other neurologic disorders with D2 antagonists (schizophrenia) or D1 agonists (PD).

#### 5. NeuroHIV

HIV is a retrovirus that attacks the immune system and causes AIDS. While HIV was initially a terminal diagnosis for the majority of infected individuals, the development of effective antiretroviral therapy (ART) has revolutionized HIV treatment and significantly improved the lifespan and quality of life of people living with HIV (PLWH). Today, more than 37 million people are living with HIV globally (UNAIDS, 2021). Importantly, ART can suppress viral replication and ameliorate the progression of infection (Cihlar and Fordyce, 2016; Saylor et al., 2016; Eggers et al., 2017; Boender et al., 2018), but it does not cure HIV. Further, long-term ART promotes chronic issues such as cardiovascular disease, metabolic symptoms, and neurologic deficits (Purohit et al., 2011). The constellation of neuropathologic, behavioral, cognitive, and motor symptoms resulting from HIV infection in the CNS are collectively known as neuroHIV, and these symptoms persist despite full viral suppression with ART (Zayyad and Spudich, 2015; Saylor et al., 2016).

The pathogenesis of HIV infection in the CNS is distinct from that in the periphery, as infection is primarily established and maintained by myeloid-lineage cells, such as macrophages and microglia (Koenig et al., 1986; Burdo et al., 2013; Saylor et al., 2016), rather than T-cells, which largely drive peripheral pathogenesis (Deeks et al., 2015). HIV can be found in the CNS as early as eight days after infection (Valcour et al., 2011) and CNS infection is thought to be mediated by the transmigration of infected CD14^+^/CD16^+^ monocytes (Williams et al., 2014; Leon-Rivera et al., 2021) and possibly infected T-cells (Honeycutt et al., 2018; Joseph and Swanstrom, 2018), across the blood–brain barrier. Within the CNS, infected monocytes mature into macrophages, and infected cells produce new virions that primarily target perivascular macrophages and microglia (Koenig et al., 1986; Burdo et al., 2013). Astrocytes may also be infected (Li et al., 2020; Lutgen et al., 2020; Eugenin et al., 2011), although there is controversy as to whether this infection is productive or whether it occurs at all (Russell et al., 2017; Ko et al., 2019). Infected myeloid cells and perhaps astrocytes can also serve as HIV reservoirs, maintaining active viral gene expression even in the presence of suppressive ART and allowing the virus to evade immune surveillance (Lamers et al., 2016; Ko et al., 2019; Wallet et al., 2019; Wong et al., 2019).

In addition to providing a platform for viral rebound (Avalos et al., 2017; Honeycutt et al., 2017; Abreu et al., 2019) and/or escape mutations (Guerveno et al., 2019; Ferretti et al., 2020), persistently infected myeloid populations are also the central drivers of neuroHIV (Navia et al., 1986; Minagar et al., 2002; Crowe et al., 2003; Yadav and Collman, 2009; Williams et al., 2013; Joseph et al., 2015; Rappaport and Volsky, 2015; Avalos et al., 2017; Clayton et al., 2017; Mallard and Williams, 2018). Infected and uninfected myeloid cells respond to HIV infection by releasing inflammatory cytokines and other factors, and infected cells also release viral proteins (Roberts et al., 2003; Burdo et al., 2013). These factors contribute to a neurotoxic environment, leading to neuronal dysfunction that continues even in the presence of ART. While ART can reduce and shift the magnitude and localization of these effects, it does not stop neurologic disease, and current research indicates that progressive neuroinflammation is central to neuroHIV in both ART-naïve and ART-treated individuals (Vera et al., 2016; Carroll and Brew, 2017; Duffau et al., 2018; Hsu et al., 2018; Alakkas et al., 2019; Winston and Spudich, 2020; Gisslen et al., 2021).

HIV-associated neurologic disturbances prominently affect dopaminergic circuits and brain regions, and the impact of dopamine on neuroHIV has been extensively discussed in recent reviews from our group and others (Nolan and Gaskill, 2019; Nickoloff-Bybel et al., 2020; Thomas Broome et al., 2020; McLaurin et al., 2021). In brief, in ART-naïve individuals, several dopaminergic regions show prominent HIV-associated neuropathology relative to nondopaminergic areas, particularly in the SbN, PFC, and striatal substructures including the caudate nucleus, putamen, and NAc (Navia et al., 1986; Aylward et al., 1993; Fujimura et al., 1997; Wiley et al., 1998; Itoh et al., 2000; Kumar et al., 2007; Casas et al., 2017; O'Connor et al., 2018). These regions have a higher viral DNA load (Aylward et al., 1993; Kieburtz et al., 1996; Fujimura et al., 1997), suggesting a correlation between HIV infection and dopaminergic brain regions. While ART reduces these effects, treated individuals still show substantial pathology in dopamine-rich regions including striatal dysfunction, increased myeloid activation, the accumulation of infected myeloid cells, and neuronal dysfunction (Anthony et al., 2005; Becker et al., 2011; Valcour et al., 2011; Alakkas et al., 2019).

These data are corroborated by studies in nonhuman primates. In simian immunodeficiency virus (SIV)-infected rhesus macaques, treatment with selegiline (monoamine oxidase inhibitor) and L-DOPA during peak viremia significantly enhanced viral infection and neuropathology in the basal ganglia, frontal cortex, and hippocampus at 6 to 8 weeks postinfection (Czub et al., 2001, 2004). In contrast to the other studies using selegiline, recent data show that selegiline treatment of macaques prior to and during acute (1–2 weeks postinfection) SIV infection reduced inflammatory and antiviral gene expression in the CNS, also reducing markers of peripheral inflammation. This correlates with earlier discussions noting that the impact of dopamine on inflammation changes depending on the environment and suggests that dopamine mediated impairment in the inflammatory and antiviral response early in infection could predispose these animals to a more robust disease course (Emanuel et al., 2022). In SIV-infected rhesus macaques that were treated with methamphetamine during chronic infection, they showed increased viral load in the caudate and enhanced expression of inflammatory factors in CNS myeloid cells (Marcondes et al., 2010; Najera et al., 2016), although chronic cocaine administration in pig-tailed macaques did not alter neuroinflammation and slightly reduced CSF viral RNA levels late in infection (Weed et al., 2012). Examination of CNS myeloid populations in methamphetamine-treated macaques using single-cell RNA sequencing showed a significant increase in the proportion of SIV-infected myeloid cells in these animals (Niu et al., 2020). These data broadly show a strong connection between HIV infection and dopaminergic dysfunction, and further suggest that these interactions are mediated by CNS myeloid populations.

The increased numbers of SIV-infected myeloid cells in methamphetamine-treated macaques correlates with in vitro data showing that exposure to elevated dopamine levels (10^−6^–10^−8^M) significantly increased viral replication in primary hMDMs (Gaskill et al., 2009) by increasing their susceptibility to HIV entry. These effects were mediated by the activation of both D1- and D2-like dopamine receptors on hMDMs and were blocked by the pan-dopamine receptor antagonist flupentixol and an inhibitor of the HIV coreceptor C-C chemokine receptor type 5 (CCR5; TAK779) (Gaskill et al., 2014). This process was not induced by other monoamine receptors and seems to be mediated by dopamine receptor activation via a noncanonical signaling pathway in which dopamine receptors trigger PKC activation and calcium release by activating D1-like receptors (Nickoloff-Bybel et al., 2019). Further studies show that D1-like transcript levels correlate with CCR5 transcript levels, and that dopamine exposure significantly increases the amount of CCR5 on the surface of hMDMs and in the human C06 microglial cell line. Further, dopamine increased HIV replication in both human microglial cell lines and induced pluripotent stem cell–derived human microglia (Matt et al., 2021). Together, these data indicate that dopamine increases viral replication by activating a noncanonical signaling pathway mediated by both types of dopamine receptors to induce changes in CCR5 that increase HIV entry into myeloid cells.

This hypothesis is supported by other studies showing that dopamine upregulates CCR5 transcription in human THP-1 macrophages, but this may be mediated by D4 signaling and could be due to the increased level of D4 on this cell type (Basova et al., 2018). A similar effect was seen in rhesus macaques, as methamphetamine increased CCR5 expression on uninfected and infected macaque microglia in vitro (Najera et al., 2016). Cocaine also upregulated CCR5 gene expression in the NAc and VTA in uninfected rats (Nayak et al., 2020). Defining the interaction between dopamine with this coreceptor is particularly important because dopamine could interfere with the effectiveness of antiretroviral drugs like maraviroc, which target CCR5 (Matt et al., 2021).

Dopamine has also been shown to affect HIV infection in CD4^+^ T-cells, although this has been less well studied and seems to be mediated by different mechanisms. Data from both primary human T-cells and the Jurkat T-cell line showed that high levels of dopamine (10^−4^M) significantly increased HIV replication by activating NF-*κ*B and Sp1 binding sites in the HIV long terminal repeat (Rohr, Sawaya et al., 1999). This process was mediated by CREB and Coup-TF (Rohr, Schwartz et al., 1999), suggesting G*_α_*_s_ activity and distinguishing it from NF-*κ*B activation in macrophages, in which G*_α_*_s_ is not responsive to dopamine receptor activation (Nickoloff-Bybel et al., 2019). A separate study showed that dopamine could activate HIV replication in the chronically infected ACH-2 lymphoblast line. These effects seemed to be mediated by oxidative stress, as they were blocked by the antioxidants glutathione and N-acetyl cysteine (Scheller et al., 2000). While the role of CD4^+^ T-cells in HIV neuropathogenesis is not well defined, these cells are capable of transmigrating and bringing HIV into the CNS (Honeycutt et al., 2018; Joseph and Swanstrom, 2018), suggesting that dopamine could affect this cell type and exacerbate disease.

These data indicate that dopamine can increase the number of infected cells in the CNS, which would accelerate the spread of HIV, increase the size of the viral reservoir, and exacerbate disease. Furthermore, as both dopamine and HIV infection can dysregulate cellular functions, the increased spread of HIV in response to changes in dopamine levels could exacerbate neuroinflammation (Nickoloff-Bybel et al., 2020). Although ART can decrease inflammation, it does not return myeloid activation to baseline levels in either the periphery or CNS (Garvey et al., 2014; Wada et al., 2015; Merlini et al., 2016; Vera et al., 2016; Galvao-Lima et al., 2017; Sereti et al., 2017; Temu et al., 2021), and myeloid inflammation still drives neuropathogenesis and disrupts neuronal function in PLWH who are using ART (Becker et al., 2011; Williams et al., 2014; Alakkas et al., 2019). Further, ART has no effect on dopamine-mediated changes in macrophage cytokine secretion (Nolan et al., 2019).

The inflammatory environment resulting from increased myeloid activity is thought to promote synaptic damage and neuronal dysfunction, contributing to the development of cognitive deficits in PLWH (Mocchetti et al., 2014; Saylor et al., 2016; Irollo et al., 2021). This finding is supported by PET scans for TSPO, indicating that increased microglial activation correlates with reduced cognitive indices (Coughlin et al., 2014; Rubin et al., 2018). PET imaging studies also showed that reductions in putamen DAT levels in PLWH were associated with poorer performance on neuropsychological tests (Chang et al., 2008), and postmortem studies showed that abnormal synaptic expression of dopamine receptors and DAT in the PFC of PLWH was linked to neuroimmune activation and increased disease severity (Gelman et al., 2006, 2012). Furthermore, disruptions in activity in dopaminergic regions diminish frontostriatal connectivity even in the presence of ART (Ipser et al., 2015; Ortega et al., 2015; Janssen et al., 2017) and promote the development of mood disorders such as depression or apathy (Fitting et al., 2015; Denton et al., 2019; Matt and Gaskill, 2019; McLaurin et al., 2021), which are highly comorbid with HIV (Nanni et al., 2015; Remien et al., 2019; Camara et al., 2020) and associated with dysregulated dopamine signaling (Dunlop and Nemeroff, 2007; Dong et al., 2020).

Changes in the dopaminergic activity and the expression of dopamine-related proteins may reflect direct adaptation to aberrant dopamine levels. The neuroinflammatory impact of dopamine, as well as its impact on myeloid cell infection, depends on the concentrations of dopamine to which immune cells are exposed in the CNS in PLWH. As summarized by McLaurin et al. (2021), there are significant changes in dopamine and dopamine metabolites in the CSF and several brain regions in response to HIV, SIV, or treatment with viral proteins such as Tat. Many of these studies showed reductions in dopamine and metabolite levels in response to HIV, although several showed increases in dopamine and metabolites during acute infection or acute Tat exposure, and these changes were inversely correlated with disease progression (Horn et al., 2013; Scheller et al., 2010). Additionally, a recent study from the Translational Methamphetamine AIDS Research Center cohort showed slight, nonsignificant increases in dopamine and HVA in the CSF of chronically infected PLWH on ART (Saloner et al., 2020). A separate study in rhesus macaques showed that SIV infection significantly increases dopamine, DOPAC, serotonin, and HVA levels in the frontal cortex, and dopamine levels in the caudate during acute infection (Emanuel et al., 2022). The conflicting results of these studies suggest that the impact of HIV on CNS dopamine is regulated by many factors, including HIV and viral proteins, disease stage, ART use, and substance use. Resolving these conflicts is critical to understanding the role of dopamine in the etiology of neuroHIV.

The mechanisms by which HIV affects dopamine levels and how dopamine levels are impacted by immune function are also unclear. Some studies suggest that the Tat protein is involved in this process, as dopaminergic neurons are sensitive to Tat-mediated neurotoxicity (Aksenova et al., 2006; Schier et al., 2017); however, neuronal loss is not common in ART-treated individuals (Gelman, 2015; Saylor et al., 2016). Tat can also disrupt DAT activity in the PFC and striatum (Zhu et al., 2016, Zhu, Ananthan et al., 2018), suggesting that this protein can modulate extracellular dopamine concentrations by regulating DAT. Additionally, as DAT can regulate phagocytosis and cytokine production in human macrophages (Mackie et al., 2022), Tat could also promote immune dysfunction and neuroinflammation through interactions with DAT. However, it is not clear that the amount of Tat secreted by infected cells is sufficient to induce these effects (Gaskill et al., 2017). A second possibility is the manipulation of dopamine catabolism, as SIV infection increases the expression and activity of MAO-B in the CNS, which correlates with increases in oxidative stress, disease severity, neuroinflammation, and levels of the myeloid cell marker CD68. This effect was also seen in the striatum of HIV-infected, encephalitic brains (Meulendyke et al., 2014) and suggests that accelerated dopamine breakdown could produce ROS to increase myeloid cell activation and drive neuroinflammation. However, this finding conflicts with other studies showing that the inhibition of MAO-B during peak viremia increased myeloid activation, neuroinflammation and viral load (Czub et al., 2001, 2004), as well as recent data showing that inhibiting MAO during acute infection was anti-inflammatory (Emanuel et al., 2022). These data also conflict with clinical trials testing the selegiline transdermal system to treat cognitive impairment in PLWH. This trial (ACT5090) showed no change in oxidative stress or disease progression and no significant benefit to cognition; however, neuroinflammation was not examined (Evans et al., 2007; Schifitto et al., 2007, 2009).

It is also possible that HIV infection alters CNS dopamine levels by dysregulating CNS metabolism. This is supported by the research showing acute SIV infection increases dopamine, DOPAC, and HVA levels in the frontal cortex of rhesus macaques (Emanuel et al., 2022), suggesting increased dopamine synthesis and subsequently increased metabolism. Other studies show that both SIV and HIV infection increase CSF dopamine and metabolite levels (Koutsilieri et al., 1997; Scheller et al., 2010), and HIV may induce CNS hypermetabolism in subcortical regions such as the basal ganglia in early infection and subcortical hypometabolism in later stages of the disease (Rottenberg et al., 1987; van Gorp et al., 1992; Rottenberg et al., 1996; Georgiou et al., 2008). Changes in dopamine and metabolite levels due to hyper- and hypometabolism at different disease stages could explain the increases and decreases in dopamine and metabolite levels seen over time during HIV infection and/or exposure to viral proteins. Further, glucose hypermetabolism was identified by PET scans with 18F-FDG in the basal ganglia, and this correlated with several forms of encephalitis and is often coupled to hypometabolism in other brain regions (Rey et al., 2012; Wegner et al., 2014; Zhao et al., 2021). In rodents, a dual tracer PET analysis of glucose (18F-FDG, 18F-GE) and microglial activation (TSPO) correlated hypermetabolism with microglial activity and neuroinflammation (Brendel et al., 2017). Further, changes in glucose metabolism regulated ROS production and inflammation, particularly inflammasome-mediated inflammation, in both human and rodent myeloid cells (Zhou et al., 2011; Garaude et al., 2016; Gleeson et al., 2016; Mills et al., 2016; Fleetwood et al., 2017; Prochnicki and Latz, 2017; Van den Bossche et al., 2017; Hughes and O'Neill, 2018). These data suggest a connection between HIV-mediated changes in dopamine metabolism and inflammation in the basal ganglia through dopamine-mediated changes in myeloid inflammation. However, it is not clear that any of these mechanisms accurately depicts the effects of HIV infection on CNS dopamine levels, and further data are needed to fully define the mechanisms by which dopamine and HIV interact to promote neurologic disease.

In addition to defining the impact of changes in the endogenous dopaminergic system on the development of neuroHIV, it is also critical to note the impact of exogenous increases in CNS dopamine on this process. SUDs are highly comorbid with HIV infection (Mimiaga et al., 2013; Degenhardt et al., 2017; Hartzler et al., 2017; Shiau et al., 2017; Leung et al., 2019), and all addictive substances increase CNS dopamine levels in mesocorticolimbic structures such as the striatum and NAc, either through direct interactions with the dopaminergic system or through indirect effects on the reward pathway (Di Chiara and Imperato, 1988; Pierce and Kumaresan, 2006; Baik, 2013). Just as important, PLWH are increasingly afflicted with chronic diseases that use dopaminergic therapeutics for treatment. Therapeutics for depression (D’Aquila et al., 2000; Zhou et al., 2005; Wood and Reavill, 2007; Moreira, 2011; Cohen et al., 2012; Vandenberg, 2012; Leggio et al., 2013; Bijlsma et al., 2014; Agius and Bonnici, 2017), Alzheimer’s disease (Zhang et al., 2004; Seeman et al., 2008; Mitchell et al., 2011), diabetes (Defronzo, 2011; Lamos et al., 2016) and non-AIDS–defining cancers such as lung and liver cancer (Lee et al., 2008; Hoeppner et al., 2015; Huang et al., 2016; Lan et al., 2017; Li et al., 2017) all act on the dopaminergic system and could increase dopamine concentrations or activate immune dopamine receptors similarly to addictive substances.

While the precise dopamine concentrations induced by addictive substances and therapeutics are not well defined in the human CNS, rodent studies of substance of misuse suggest concentrations of approximately 10^−7^M to 10^−5^M, depending on the brain region and substance (Matt and Gaskill, 2020). Because the volume of CNS tissue and number of cells exposed to dopamine is directly proportional to the dopamine concentration (Matt and Gaskill, 2020), drug-mediated amplification of dopamine release could increase the number of immune cells exposed to increased dopamine levels (Peters and Michael, 2000; Venton et al., 2003; Spuhler and Hauri, 2013) The data also suggest that SUDs may have synergistic effects with HIV on cerebral metabolism, increasing subcortical hypermetabolism and inducing the premature emergence of cortical hypometabolism, which could further amplify the effects of SUDs on dopamine concentrations (Georgiou et al., 2008). As dopamine has substantial impacts on cytokine production, migration, and other immune functions, exposure to elevated dopamine is likely to dysregulate these functions. As the mesocorticolimbic regions, which generally show increased dopamine, also show elevated levels of neuropathology and dysfunction during neuroHIV, the use of addictive or therapeutic substances could accelerate or exacerbate the progression of neuroHIV.

The specific effects of elevated dopamine and SUDs on neuroHIV has been discussed in recent reviews (Chilunda et al., 2019; Nickoloff-Bybel et al., 2020), and many studies have examined the effects of specific substances, such as methamphetamine and cocaine, on different types of immune cells. While progress in this area has slowed because of the challenges involved in HIV infection in rodents and limited access to nonhuman primates, the development of new rodent model systems and sophisticated cocultures and organoids has the potential to rapidly advance these important studies. Overall, the data in this section show that the progression of neuroHIV is connected to changes in the dopaminergic system, and these changes can mediate specific effects on immune cells that could accelerate or exacerbate disease. Unfortunately, the mechanisms by which these effects occur are still unclear, and significantly more resources and studies are needed to define these effects so that they can be specifically targeted and ameliorated in PLWH, particularly those vulnerable populations of PLWH with SUD or who are using dopaminergic therapeutics.

### B. The Periphery

#### 1. Introduction

Dopamine is present in the bloodstream and most peripheral organs (Matt and Gaskill, 2020), suggesting a role for dopaminergic regulation of many systemic functions, and indicating that the effects of dopamine are not limited to the CNS. This section reviews the activity of dopamine in a number of peripheral compartments and discusses the potential role of dopaminergic immunoregulation in the development and progression of diseases affecting each region. As in the CNS, many of these diseases are also treated with therapeutics that impact the dopaminergic system. Thus, precisely defining the mechanisms by which dopamine signaling regulates pathologic immune activity in the periphery could provide new understanding for a number of diseases, generate valuable contraindication data, and greatly improve overall therapeutic efficacy.

#### 2. Gastrointestinal System

The GI system is a major source of peripheral dopamine, accounting for nearly half of all dopamine produced in the body (Eisenhofer et al., 1997). Dopamine concentrations in various GI tissues and fluids in humans and rodents are in the nanomolar to micromolar range (Matt and Gaskill, 2020). Local supplies of dopamine within the GI muscularis and epithelium are modulated by enteric neurons (Li et al., 2004), stomach and intestinal epithelial cells (Mezey et al., 1998; Tian et al., 2008; Li et al., 2019), gut-resident immune cells (Mezey and Palkovits, 1992), and the gut microbiome (Lyte, 2013; Strandwitz, 2018). The ubiquity of TH throughout the GI tract also suggests a high capacity for dopamine biosynthesis, although it is unclear how much of the dopamine in the GI tract is an intermediate of epinephrine and norepinephrine production (Mittal et al., 2017). Dopamine concentrations are heterogeneous across the GI tract, ranging from 10^−9^M in the small intestine to as high as 10^−4^M in the colon (Matt and Gaskill, 2020).

**Fig. 8:**
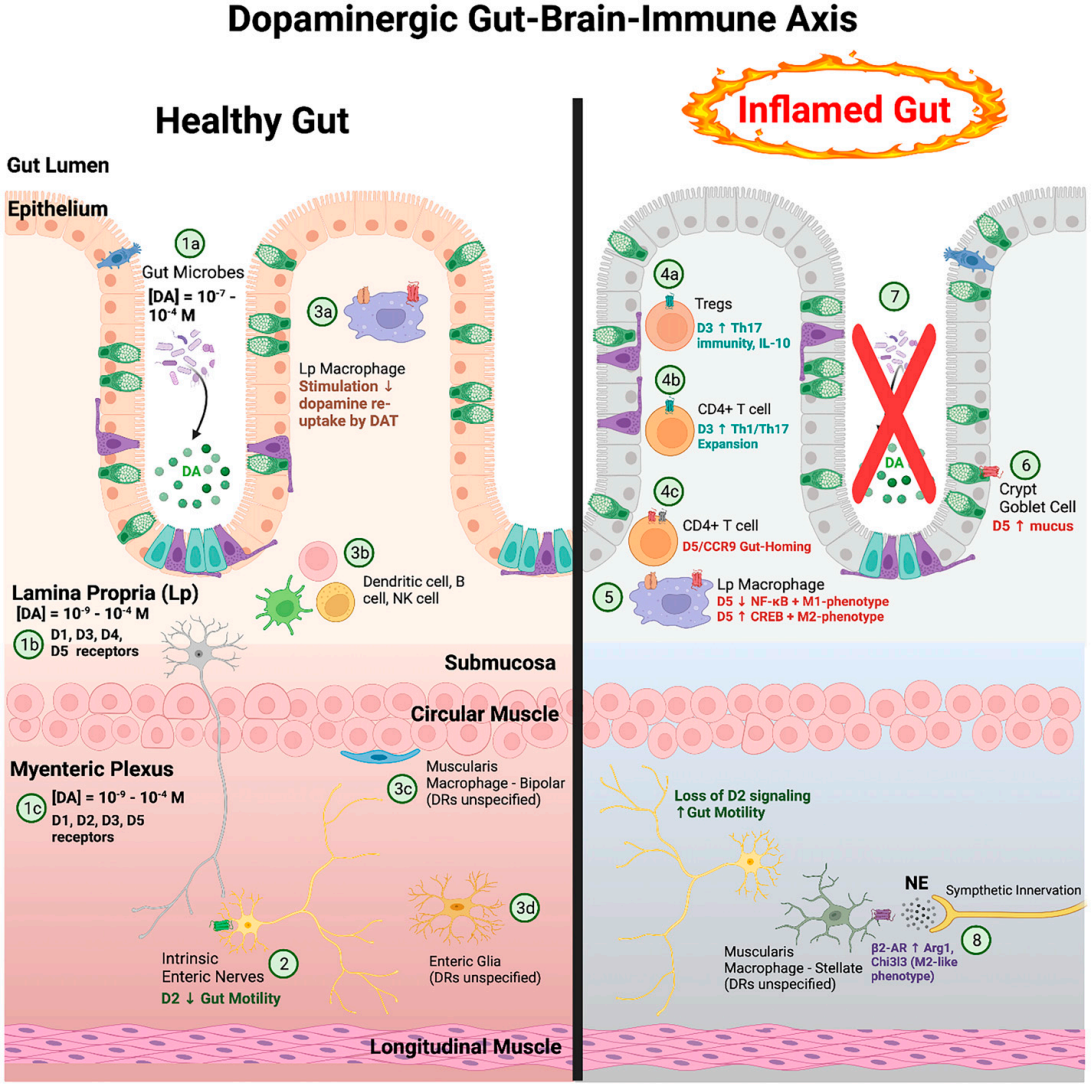
Dopaminergic gut–brain–immune axis. In the healthy gut (left), a significant portion of dopamine is produced by intestinal microbes (1a) in the gut lumen, which may be shuttled into the lamina propria (Lp) and submucosa. Dopamine concentrations vary from the nanomolar to the micromolar range in the Lp and submucosa of the intestine (1b), which express all dopamine receptors except D2. Dopaminergic neurons in the myenteric plexus produce similar concentrations of dopamine locally (1c), and this layer has immunoreactivity for all dopamine receptors except D4. Activation of D2 in the myenteric plexus reduces GI motility (2). Stimulation of macrophages in the mucosal layer reduces their capacity for dopamine reuptake by DAT (3a) and may increase extracellular dopamine concentrations for autocrine/paracrine signaling. The impact of dopamine in GI homeostasis is not well understood in several other cell types such as dendritic cells, B-cells, and NK cells (3b); bipolar macrophages (3c); enteric glia (3d); and stellate macrophages (8). Several models of inflammatory bowel disease (right) have shown an inflammatory role of dopamine, primarily through D3 and D5 on T_regs_ (4a) and CD4^+^ T-cells (4b and 4c). D5 stimulation is associated with polarization of Lp macrophages to M2-phenotype (5) and increased mucus production by intestinal goblet cells (6). Depletion of the microbiome reduces dopamine levels in the mucosa and exacerbates gut inflammation, which may be attenuated by D1-like agonists (7). Stellate macrophages (8) express the *β*2-adrenergic receptor, which may be activated by high concentrations of dopamine, and form neuroimmune connections with extrinsic catecholaminergic neurons and intrinsic enteric neurons. Created with BioRender.com.

Several studies and reviews suggest that the microbiome is involved in maintaining dopamine homeostasis in the GI tract (Lyte, 2013; Sampson and Mazmanian, 2015; Strandwitz, 2018; González-Arancibia et al., 2019). Supporting this conclusion, depletion of the gut microbiota decreases dopamine synthesis or increases dopamine turnover in the brain and intestine (Asano et al., 2012; Diaz Heijtz et al., 2011), and GI inflammation associated with microbial depletion can be ameliorated by D1-like agonists (Xue, Zhang et al., 2018). The GI tract is also sensitive to dopaminergic disruption in other regions, as 6-hydroxy-dopamine-mediated destruction of nigrostriatal dopamine pathways can disrupt gastric and intestinal motility, as well as increase colonic inflammation (Toti and Travagli, 2014; Pellegrini et al., 2016). Studies show that intestinal motility in the GI tract can be regulated by D2 activity (Kusunoki et al., 1985; Takahashi et al., 1991; Dive et al., 2000; Tonini et al., 2004; Li, Schmauss et al., 2006), and genetic polymorphisms in Drd2 that reduce receptor expression are linked to refractory ulcerative colitis in humans, suggesting an important role of GI motility in maintaining gut immunity (Magro et al., 2006). A summary of the impact of dopamine on the gut during homeostatic and pathologic conditions can be seen in Fig. 8.

Although the effects of dopamine on GI motility seem to be primarily mediated by activation of D2, all five dopamine receptor subtypes can be found along the GI tract, with variable prevalence depending on the tissue layer and cell type. Expression of D2 is found only on enteric neurons within the myenteric plexus; D4 is only found in the mucosal layer; and D1, D3, and D5 can all be found in both the mucosal layer and the myenteric layer of the intestine (Sclafani, 2001; Li, Schmauss et al., 2006). Dopaminergic neurons in the enteric nervous system also express high levels of TH and the DAT, but they lack the dopamine *β*-hydroxylase enzyme that converts dopamine into norepinephrine (Li et al., 2004). Within the myenteric plexus of the intestine, dopaminergic neurons are localized near muscularis macrophages, a specialized immune cell that is thought to regulate GI motility by communicating with enteric neurons and autonomic neurons from outside the GI (Li, Schmauss et al., 2006; Bogunovic et al., 2009; Gabanyi et al., 2016). Motility is further regulated by enteric glial cells, which are comparable to CNS astrocytes in morphology (Reichenbach et al., 1992) and produce similar phenotypic markers (Ferri et al., 1982; Jessen and Mirsky, 1983; Jessen et al., 1983; McClain et al., 2014). Like astrocytes, enteric glia are activated by intracellular calcium via GPCR signaling, which drives GI contractility in mice (McClain et al., 2015). While enteric glial cells express several receptors for neurotransmitters and neuromodulators (Grubišić and Gulbransen, 2017), their dopamine receptor profile is poorly defined. Interestingly, reactive glia have been shown to contribute to inflammatory responses in the GI through activation of NF-*κ*B pathways in both mouse models of dextran sulfate sodium–induced colitis (Esposito et al., 2014; MacEachern et al., 2015) and humans with ulcerative colitis (Cirillo et al., 2011; Esposito et al., 2014). Alterations in dopaminergic tone within the myenteric layer could alter both calcium and NF-*κ*B signaling in enteric glia, thus serving as a potential modulator of GI motility and inflammation.

These findings suggest that dopaminergic neurons, muscularis macrophages, enteric neurons, and enteric glia share a microenvironment that enables dopaminergic neuroimmune signaling. There are a number of distinct subpopulations of macrophages in the gut that can respond to dopamine, and the heterogeneity of these populations is partially governed by their anatomic niche within the intestinal wall (Hadis et al., 2011; Asano et al., 2015; Gabanyi et al., 2016; De Schepper et al., 2018). Macrophages in the outermost layer of the mucosa, muscularis macrophages, are associated with tissue maintenance and protection and can be further classified into two distinct morphologies: stellate and bipolar (Phillips and Powley, 2012; Gabanyi et al., 2016). Stellate muscularis macrophages exhibit a morphology similar to microglia, enabling their dendriform processes to form neuroimmune connections with several enteric neurons, glia, and possibly dopaminergic neurons to regulate GI motility and health (Muller et al., 2014). These cells highly express *β*2 noradrenergic receptors (Gabanyi et al., 2016), to which dopamine binds at a lower affinity than norepinephrine, suggesting a potential mechanism by which high dopamine levels facilitate muscularis macrophage functions (Freddolino et al., 2004). A recent study showed subpopulations of Iba1^+^ macrophages expressed DAT in the lamina propria and submucosa but not in the muscularis. A population of Iba1^+^/DAT^+^ cells were also found near lymphoid-like follicles or MAP2^+^ ganglia. This study showed that DAT activity modulates macrophage immune functions, further suggesting the possibility of neuroimmune communication (Mackie et al., 2022). In addition to these specific data, it is critical to consider that the gut mucosa contains relatively high concentrations of dopamine compared with most other tissue compartments and is under constant surveillance by large numbers of immune cells, including DCs, NK cells, T-cells, and B-cells, which all express dopamine receptors and respond to dopamine. It is likely that these cells regularly encounter dopamine concentrations that influence immune function as previously described and suggests that dopamine may play a significant role in the homeostatic regulation of mucosal immunity.

##### a. Inflammatory Bowel Disease

Inflammatory bowel disease (IBD) is an umbrella term for Crohn’s disease (CD) and ulcerative colitis, which are two distinct disorders that are characterized by chronic, progressive inflammation in the GI tract. Inflammation in ulcerative colitis is localized to the mucosal and submucosal layers of the colon and rectum, whereas inflammation in CD can be found throughout the GI tract and affects all layers of the intestinal wall (transmural inflammation). The etiology of IBD is not well understood and is thought to involve multiple mechanisms that initiate and support an inflammatory phenotype, including microbiome dysregulation, neuroimmune signal dysregulation, and immune cell overactivation (Plichta et al., 2019; Kobayashi et al., 2020; Roda et al., 2020). As noted in the prior section, the GI system contains a sophisticated immune repository, including T-cells, DCs, and macrophages, as well as a variety of granulocytes, and each cell type differs in its role based on location and gene expression (Wu and Wu, 2012; Matt and Gaskill, 2020; Ugalde et al., 2020). Well-regulated interactions between these discrete immune cell populations, as well as with the enteric nervous system, intestinal walls, and the gut microbiota, are critical for proper gut homeostasis, and disruptions in these interactions play important roles in numerous gut diseases, including IBD.

During IBD, both serotonin and dopamine levels are elevated in the mucosa and decreased in deeper gut tissue relative to those in healthy individuals, which may be due to impaired synthesis and cellular storage of dopamine in the enteroendocrine cells and enteric nervous system. These changes in dopamine concentrations can influence the immune signals that lead to pathology (Magro et al., 2002; Coates et al., 2004), as indicated in Fig. 8. Decreased dopamine levels correlated with the development of colitis in both humans and rodents, and rodent studies showed that dopamine signaling through D5-mediated changes in macrophage phenotype that decreased inflammation (Magro et al., 2004; Liu, Wu et al., 2021). However, other rodent studies showed that treatment with the pan-dopamine receptor antagonist berberine decreased colitis, conflicting with the potential anti-inflammatory effects of D5 signaling. Berberine reduced the production of inflammatory cytokines such as IL-1*β*, IL-6, and TNF-*α*, increased the anti-inflammatory cytokine IL-10 and increased the ratio of M1 and M2 macrophages to levels seen in healthy rodents. However, berberine also reduced IL-17 expression, increased IL-22 expression and suppressed T_h_1 and T_h_17 cell expansion (Hong et al., 2012; Kawano et al., 2015; Li et al., 2015).

D3 may also be an important regulator of IBD, as D3 deficiency in murine regulatory T-cells diminished inflammatory manifestations of colitis by attenuating IL-10 production and reducing the acquisition of gut-tropism in these cells. Mice lacking D3 were also resistant to dextran sulfate sodium–induced colitis in a cohousing paradigm, suggesting that environmental microbiota transfer may not play a role in the D3-deficient phenotype (Ugalde et al., 2021). Studies have shown a role for D2 in regulating inflammation, as both the D2 agonists quinpirole and cabergoline (Tolstanova et al., 2015), prodrugs derived from the D2 antagonists amisulpride (Kim, Kim et al., 2019) and metoclopramide (Yang et al., 2018) ameliorated colitis. A separate study showed that dopamine protected against indomethacin-induced colitis but that antagonizing D2 with sulpiride or domperidone blocked this effect, increasing IL-10 production and blocking indomethacin-induced intestinal hypermotility (Miyazawa et al., 2003). These data clearly show that dopamine signaling impacts IBD development (Mittal et al., 2017), but the effects of specific dopamine receptor signaling are unclear, possibly due to discrete effects of dopamine signaling that depend on the species and/or colitis model used in the experiment.

These data also suggest that dopamine likely influences inflammation through activation and intercellular communication in several cell types. In DCs, D5 signaling induces RAR-related orphan receptor gamma and increases IL-23 and IL-12 (Prado et al., 2012; Prado et al., 2018), which are key cytokines that regulate adaptive immunity in CD (Aggeletopoulou et al., 2018). In mice, signaling through D5 combined with increased D3 expression led to T_h_1/T_h_17 differentiation, which contributed to the persistence of gut inflammation (Contreras et al., 2016). In a separate study using mouse and human systems, an increase in inflammatory CD4^+^ effector T-cell migration into the intestinal mucosa was mediated by D5 heteromerization with the chemokine receptor CCR9. Inflammation was also driven by D3, as Drd3 deficiency improved the immunosuppressive capacity of T_regs_, and D3 signaling inhibited the migration of these cells into the lamina propria region of the gut (Ugalde et al., 2021). While D5 signaling did not alter the expansion of T_h_1 and T_h_17 cells among CD4^+^ T-cells, the loss of D5 reduced the severity of GI inflammation (Osorio-Barrios et al., 2018, 2021).

In addition to regulating IBD, dopamine signaling in the gut and dopamine-induced changes in gut immune function and inflammation can impact pathology in other organ systems. MPTP-mediated ablation of enteric dopaminergic neurons, which reduced dopamine release in the gut, increased the production of inflammatory cytokines by invariant NK T-cells during hepatitis by preventing dopamine-induced repression of inflammatory signaling via the D1-like receptor-PKA pathway (Xue, Zhang et al., 2018). Gut inflammation also seems to influence CNS dopamine signaling and associated neuroinflammation, as patients with IBD had a 46% increased risk of developing PD (Zhu et al., 2019). In mouse models, colitis increased CNS CD8^+^ T-cell infiltration and enhanced the inflammatory and deleterious effects of LPS and MPTP on dopaminergic neurons within the nigrostriatal pathway (Villaran et al., 2010; Houser et al., 2021). These data show that dopamine impacts both IBD and secondary pathologies associated with IBD, indicating a fertile area for future research.

These data indicate that different dopamine receptor subtypes mediate both pro-and anti-inflammatory activity in the gut and may act differently on discrete types of immune cells, as D5 activation was shown to both decrease colitis by altering M1/M2 ratios and increase T_h_1/T_h_17-mediated inflammation. These data indicate that future studies in these areas, as well as therapeutics targeting dopamine-mediated immune activity, should carefully evaluate not only which dopamine receptors/transporters are involved but also which cell type(s) are being affected by signaling through those receptors. These studies will be critical in effectively leveraging the dopaminergic pathway to treat IBD, as they define the diverse impacts of dopamine receptor and transporter activity on different immune cell populations. Despite the gaps in knowledge, the effects of dopamine signaling on gut inflammation and pathologies make it a strong target for new IBD therapeutics, as well for studies examining the specific receptors and cell types that drive the immunomodulatory properties of this neurotransmitter.

#### 3. Skin

The skin plays a vital role in regulating a homeostatic environment and has innate and adaptive immune roles that are still being defined. The skin consists of three main layers—the epidermis, dermis, and hypodermis—and contains numerous cell types throughout these layers that contribute to homeostatic maintenance and immune regulation (Kabashima et al., 2019; Nguyen and Soulika, 2019). Keratinocytes are the most prominent cell type in the epidermis and play important roles in pathogen detection and wound healing. Data on dopamine signaling in keratinocytes during wound healing suggest regional specificity and indicate discrete roles for D2 and D4 (Fuziwara et al., 2005). Autocrine and paracrine signaling of dopamine and other catecholamines and neuropeptides allows keratinocytes to mediate the local environment (Ramchand et al., 1995; Parrado et al., 2017). Although the pathway by which dopamine is produced in the skin is not clear, several cell types may be capable of producing dopamine or its precursors.

**Fig. 9:**
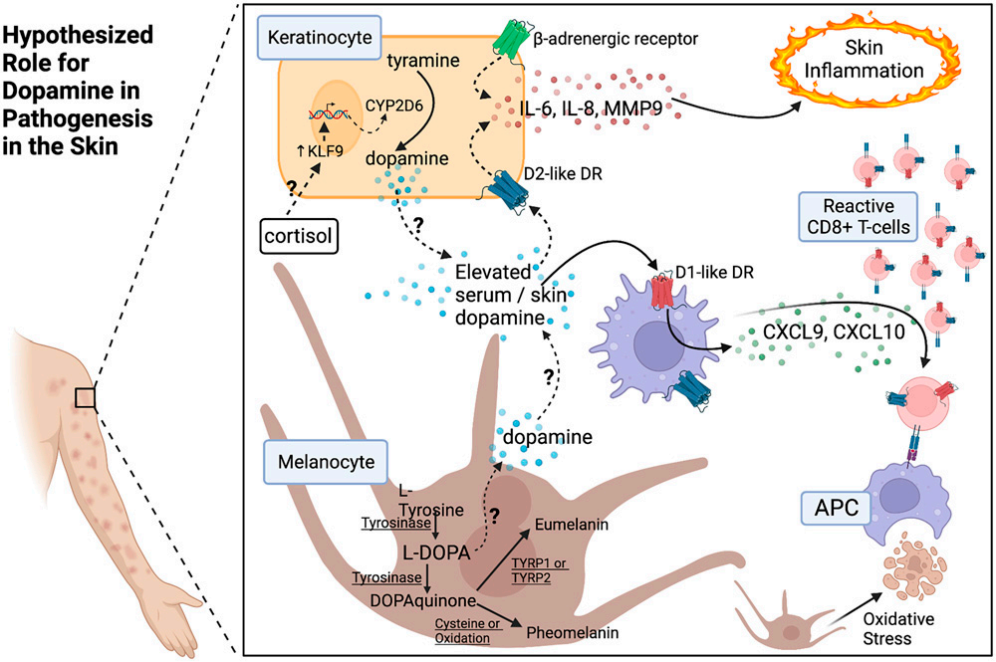
Hypothesized role for dopamine in pathogenesis in the skin. Dopaminergic signaling has been hypothesized to drive pathogenesis in the skin by immune regulation. Keratinocytes, the most abundant cell type in the epidermis, mediate inflammation in the local environment from autocrine and paracrine signaling of dopamine through *β* adrenergic or D2 receptors. Although an unclear pathway (depicted by dashed arrows), stress hormones such as cortisol can increase Kruppel-like factor 9 transcription factor expression to then induce the upregulation of cytochrome P450 2D6 (CYP2D6). CYP2D6 within keratinocytes can then metabolize tyramine to dopamine which can contribute to the elevated serum/skin dopamine observed in pathology. Melanin synthesis is connected to dopamine synthesis as melanocytes convert L-tyrosine to L-DOPA using the rate limiting enzyme tyrosinase which also converts L-DOPA to DOPAquinone in these cells. DOPAquinone is metabolized to eumelanin and pheomelanin by tyrosinase-related proteins (TYRP1, TYRP2) or cysteine and oxidation reactions, respectively. As depicted by the dashed lines, there is an unclear mechanism of how dopamine is produced in these cells to contribute to the elevated serum dopamine in pathology. Elevated levels of dopamine in the serum and skin in pathology has been shown to act on D1-like receptors on macrophages to increase production of chemokines CXCL9 and CXCL10, which recruits reactive CD8^+^ T-cells. In the presence of oxidative stress, melanocytes undergo apoptosis and are phagocytized by antigen presenting cells that interact with the recruited CD8^+^ T-cells. Dashed lines represent pathways that have been hypothesized by some groups and solid arrows represent more established pathways. Created with BioRender.com.

One such pathway may be mediated by cytochrome P450 2D6 (CYP2D6), which can metabolize both p- and m-tyramine to dopamine in the brain and liver (Wang et al., 2009; Wang, Li et al., 2014). Expression of CYP2D6 is readily detected in human skin biopsies (Oesch et al., 2007), and in keratinocytes, cortisol can induce Kruppel-like factor 9 expression, which upregulates CYP2D6 in other model systems (Pan, Ning et al., 2017). This synthesis pathway could explain the increases in skin dopamine levels in response to tyramine perfusion and microdialysis measurements (Leis et al., 2004) and suggests a mechanism by which stress and inflammation could modulate the immune environment within the skin through dopamine signaling. An alternative dopamine synthesis pathway may be mediated by melanocytes, which are found primarily within hair follicles or the basal layer of the epidermis. These cells are the source of melanin and pigmentation within the skin, delivering it to keratinocytes through dendritic projections. Melanin synthesis is intimately connected with the metabolic pathway of dopamine synthesis (Cichorek et al., 2013), as tyrosinase, the rate limiting enzyme in melanin production, converts L-tyrosine to L-DOPA, as does TH. Tyrosinase then utilizes L-DOPA to generate the oxidized intermediate DOPA quinone, which is used to generate eumelanins or pheomelanin by tyrosinase-related proteins (TYRP1, TYRP2) or cysteine and oxidation mechanisms, respectively (Slominski et al., 2004). This is summarized in Fig. 9.

While melanocytes can produce L-DOPA, their capacity to generate dopamine has not been directly shown, and the expression of AADC or CYP2D6 have not been observed in these cells (Meiser et al., 2013). However, tyrosinase-dependent mechanisms in melanocytes do regulate D1 activation through sensory neurons, which can mediate mechanical sensitivity through transient receptor potential cation channel subfamily V member 1 and Piezo2 channels (Ono et al., 2017). Melanocyte contributions to dopamine pools in the skin may be dependent upon L-DOPA secretion and subsequent conversion to dopamine through AADC extracellularly or in other cell types (Eisenhofer et al., 2003). Additional research is needed to provide clearer insight into the mechanisms mediating dopamine metabolism and signaling in the skin and how these processes contribute to cutaneous disease and inflammation (Slominski et al., 2012).

##### a. Psoriasis and Vitiligo

Psoriasis is as an autoimmune disease that involves keratinocyte hyperproliferation and hyperkeratosis. It affects an estimated 2% of the global population and is thought to have genetic and psychologic components, particularly involving the neuroendocrine stress response (Tampa et al., 2018; Torales et al., 2020). The etiology of psoriasis is poorly understood; however, it is significantly associated with neurologic diseases involving dopamine dysregulation, suggesting a role for dopamine in disease pathogenesis. The risk of schizophrenia is 41% greater in patients with psoriasis, potentially involving dysregulation in T_h_17 cells, which are implicated in both psoriasis and schizophrenia (Ungprasert et al., 2019) and are strongly affected by dopamine. This connection is supported by a 5-year prospective study showing that the rates of psoriasis in schizophrenic patients were estimated to be 2.82%, compared with 1.17% in controls (Yu et al., 2017). Multiple studies have also shown an increased risk of PD among psoriasis patients (Sheu et al., 2013; Ungprasert et al., 2016). Interestingly, a case study described a 74-year-old woman who had been diagnosed with PD, developed inverse psoriasis, and saw a remission of her psoriatic symptoms when treatment with L-DOPA was initiated (Rojo Suarez et al., 2017).

One of the strongest connections between dopamine and psoriasis is found in data showing patients with psoriasis had nearly threefold higher serum levels of dopamine. The diagnostic potential of increased serum dopamine levels was evaluated in psoriatic patients and had a sensitivity of 87% and specificity of 90% compared with nonpsoriasis patients (Wardhana et al., 2019). The role of dopamine in psoriasis pathogenesis is further supported by multiple case studies reporting the worsening of psoriasis in patients treated with the atypical antipsychotic olanzapine, which acts as a high-affinity antagonist at both dopamine and serotonin receptors (Ascari-Raccagni et al., 2000; Latini and Carducci, 2003; Chepure and Ungratwar, 2017). The rs4680 single nucleotide polymorphism (SNP) in COMT, which causes a three- to fourfold decrease in the dopamine metabolizing activity of this enzyme (Lachman et al., 1996), increases the risk of psoriasis (Sobolev et al., 2019), although variable changes in COMT levels in response to different treatments suggest increases in systemic or local dopaminergic tone are only a part of the disease process (Souteiro et al., 2013).

Preclinical data also indicate a role for dopamine, as topical formulations of D1 agonist fenoldopam reduced psoriatic lesions in an imiquimod-induced psoriasis model (Doppalapudi et al., 2020). Studies in keratinocyte cell lines suggest that activation of D2-like receptors drives keratinocyte inflammatory activity and dopamine can alter the activity of matrix metalloproteinases and wound healing, potentially affecting inflammation (Fig. 9). However, many of these effects seem to be mediated via *β*-adrenergic receptors (Parrado et al., 2012, 2017), and with the contrasting findings on the role of D1- and D2-like activation, the specific processes by which dopamine could influence keratinocyte activity to drive psoriasis remain unclear. Further studies are needed to better understand the role of dopamine in psoriasis etiology and to define the common immune mechanisms connecting psoriasis with neurologic disorders such as schizophrenia and PD.

Vitiligo is one of the most common skin depigmentation disorders, resulting in the selective loss of melanocytes in the skin and creating well-demarcated regions that are devoid of melanin. Vitiligo affects all ethnicities and skin types, and the causes of this disease are currently unknown. Current theories suggest a combination of several mechanisms, including autoimmune, genetic, inflammatory, and oxidative stress components (Xie et al., 2016; Bergqvist and Ezzedine, 2020).

Tyrosinase and several other components involved in the melanin biosynthetic pathway act as autoantigens in some cases of vitiligo, in part due to ROS-induced oxidative damage derived from the production of dopamine quinones during melanin synthesis (Niu and Aisa, 2017). These ROS species can inflict oxidative damage on the cell, especially at the membrane level, and induce stress responses. The interface between the cellular stress response and the innate immune response is thought to initiate the vitiligo. Specifically, Langerhans cells and macrophages present melanocyte proteins generated via oxidative damage and melanocyte cell death. This induces the recruitment of adaptive immune cells, including autoreactive CD8^+^ T-cells specific to melanocyte-derived proteins. These CD8^+^ T-cells appear to be dependent on IFN-*γ* signaling, as CXCL9 and CXCL10 were necessary for the recruitment of CD8^+^ T-cells to melanocytes and were present at significantly increased levels in vitiliginous skin. The persistence of these autoreactive CD8^+^ T-cells seems to be the underlying cause for ongoing disease and relapse after treatment (Xie et al., 2016; Bergqvist and Ezzedine, 2020).

A recent study demonstrated the specific importance of CD8^+^ T resident memory (T_rm_) cells in vitiligo. These are resident populations of T-cells that establish themselves in a specific site, such as the skin, and are removed from circulation, proliferating and expanding within the site and have extended lifespans, on the magnitude of years in humans. Selective elimination of the CD69^+^CD103^+^CD8^+^ T-cells (markers for T_rm_ cells) within the epidermis in vitiliginous mice durably reverse vitiligo (Mackay et al., 2015; Richmond et al., 2018). Given the broad influence of dopamine on T-cell maturation, dopamine may influence vitiligo by mediating T_rm_ cell development and T_reg_ exhaustion. In skin samples from vitiliginous patients, studies show increased levels of D1-like dopamine receptors, AADC, and MAOs (Reimann et al., 2012), while elevated levels of dopamine itself have been found in vitiligo patient skin, peripheral blood, and urine (Morrone et al., 1992; Basnet et al., 2018). This dysregulation of dopamine homeostasis could occur via elevated L-DOPA due to impaired tyrosinase activity in melanocytes during early disease stages. As it is not clear melanocytes can convert L-DOPA to dopamine, other cell types may be involved. Keratinocytes could be producing dopamine through the activity of CYP2D6, although the elevation of AADC suggests the canonical dopamine synthesis pathway (Fig. 2) is involved.

The increased dopamine levels in vitiliginous skin could increase T-cell numbers by increasing monocyte transmigration and subsequent macrophage activity, including dopamine mediated production of CXCL9 and CXCL10 (Fig. 9) recruiting more CD8^+^ T-cells to the melanocytes. Activation of D3 on naïve CD8^+^ T-cells also increases migration and the expression of integrin *α*4*β*1 (very late antigen 4) and integrin *α*4*β*1 (very late antigen 5), cellular adhesion molecules (Watanabe et al., 2006), potentially increasing CD8^+^ T-cell numbers in skin. Activation of D4 could also influence T_rm_ numbers, as this receptor can regulate expression of Kruppel-like factor 2 (Sarkar et al., 2006), a transcription factor whose downregulation is essential for T_rm_ development. Increase in skin dopamine levels could also enhance CD8^+^ activity indirectly, by mediating exhaustion in T_regs_. Dopamine can suppress T_reg_ activity via D1-like receptors, increasing CD8^+^ T-cell proliferation (Kipnis et al., 2004), and T_reg_ can also store and release dopamine to promote autocrine regulation of IL-10 and TGF-*β* production (Cosentino et al., 2007). Indeed, decreases in T_reg_ numbers (Klarquist et al., 2010) and immunoregulatory function (Lin et al., 2014) have been reported in the skin of vitiligo patients. Increases in expression of the immune checkpoint gene CTLA4 and immunoregulatory gene PTPN2 have been observed in patient skin alongside increases in COMT (Tanwar et al., 2022).

While these activities are likely important in balancing and regulating the immune response, in environments with aberrant dopaminergic regulation such as vitiliginous skin, they may foster increased inflammation through inappropriate downregulation of the T_reg_ immunosuppressive response. Further study is needed to elucidate whether there is a direct role of dopamine dysregulation on immune dysregulation reported in patient skin. Overall, these data indicate that dysregulation of local dopamine pathways increases dopamine levels in the skin, disrupting immune homeostasis and triggering sustained, aberrant reactions that promote the develop of both vitiligo and psoriasis. The mechanisms by which this occurs are not clear, but the connection between dopamine and several facets of these diseases suggests that targeting these dopamine pathways could be a novel strategy to ameliorate these skin diseases.

#### 4. Bone and Joints

Dopamine is present in the bone marrow (Marino et al., 1997; Maestroni et al., 1998), but until recently, there was relatively little interest in the interactions between dopamine and bone activity. However, recent data indicate that there is bidirectional communication between bone and the nervous system (Gerosa and Lombardi, 2021), which supports the observation of a direct interaction between bone health and neurologic disease (Kelly et al., 2020), many of which are affected by dopamine. Bone growth is regulated by specialized cell types known as osteoblasts, which produce matrix products and transport minerals to form and reshape bone, and osteoclasts, which are macrophage-like cells that are responsible for breaking down bone tissue. Numerous studies show that both osteoblasts and osteoclasts express all subtypes of dopamine receptors, although the receptor profile varies between the two cell types (Hanami et al., 2013; Lee, Tsang et al., 2015; Yang et al., 2016; Motyl et al., 2017; Handa et al., 2019; Wang, Han et al., 2021; Zhu et al., 2022). For example, RNA sequencing analyses of human and murine osteoblasts and osteoclasts confirmed gene expression of all dopamine receptors and showed increased expression of Drd1, Drd2, and Drd3 during osteoclastogenesis, while mouse osteoblast precursors and mature osteoblasts expressed higher levels of Drd3 and Drd4 (Handa et al., 2019). Dopamine synthesis proteins are also important in healthy bone development, as a homozygous DAT gene deletion in adult C57/B6J mice produced osteopenia (Bliziotes et al., 2000).

**Fig. 10:**
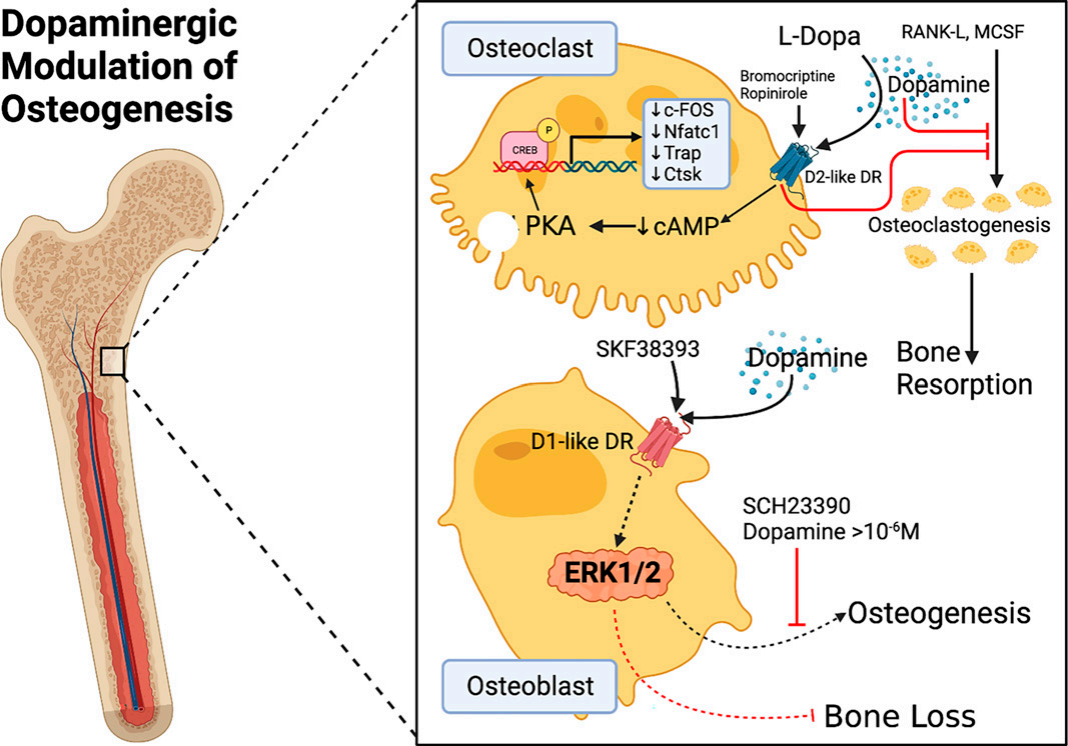
Dopaminergic modulation of osteogenesis. Dopamine activity may drive osteogenesis by regulation of osteoclasts and osteoblasts. The macrophage-like osteoclasts respond to dopamine and dopaminergic agents such as bromocriptine and ropinirole at the D2-like receptors. Increased D2-like signaling leads to a reduction in osteoclastogenesis via reduction of cAMP and PKA activity leading to inhibition of CREB phosphorylation and subsequent transcription of osteoclastic markers c-FOS, Nfatc1, tartrate-resistant acid phosphatase, and Ctsk. Dopamine in the bone and through D2-like signaling on osteoclasts inhibits RANK-L and M-CSF induced osteoclastogenesis and therefore bone resorption. Osteoblast activity in response to dopamine appears to be regulated by D1-like receptor signaling. Dopamine or the D1-like receptor agonist SKF38393 act on the D1-like receptors to mediate signaling through ERK1/2, which induces osteogenesis and inhibits bone loss. SCH23390, a D1-receptor antagonist, and high concentrations of dopamine can both inhibit this process. Dashed lines represent pathways that have been hypothesized and solid arrows represent more established pathways. Red arrows depict inhibitory pathways and black arrows are stimulating/activating pathways. Created with BioRender.com.

Several studies have shown that dopamine receptor activity can regulate both osteoblast and osteoclast differentiation and function (Fig. 10). Most experiments studying osteoclast differentiation induce osteoclastogenesis in human or rodent myeloid cells via treatment with macrophage colony-stimulating factor (M-CSF) and receptor activator of nuclear factor kappa-Β ligand (RANKL), a process that is inhibited with dopamine treatment. Osteoclastogenesis was reduced by dopamine in human CD14^+^ monocytes (10^−10^–10^−6^M) (Hanami et al., 2013), rodent BMDM (10^−8^M–10^−10^M) (Hanami et al., 2013; Yang et al., 2016), primary murine bone marrow stromal cells (10^−9^–10^−6^M) (Motyl et al., 2017), and the murine RAW264.7 macrophage cell line (10^−4^M–10^−5^M) (Wang, Wang et al., 2021, 2021). Treatment with L-DOPA, as well as ropinirole and bromocriptine (D2-like receptor agonists), also suppressed proliferation and RANKL-induced osteoclastogenesis in BMDM (Handa et al., 2019). The dopamine-mediated reduction in osteoclast differentiation was also shown in vivo in mice with titanium particle–induced osteolysis (Yang et al., 2016). In contrast with the previously cited studies, in vivo treatment with L-DOPA increased bone loss, while treatment with D2 agonists reduced osteoclastogenesis but did not increase bone mass (Handa et al., 2019).

In all these studies, the reduction in osteoclastogenesis was accompanied by a dopamine-mediated reduction in osteoclastic markers (c-Fos, Nfatc1, Trap, Ctsk) that was generally found to be mediated through D2-like receptor signaling. The effects of dopamine could be replicated with D2-like receptor agonists, such as pramipexole, and blocked with D2-like receptor antagonists, such as haloperidol. Treatment with D1-like agonists and antagonists had no effect on osteoclastogenesis. Analysis of the D2-like receptor signaling mediating this process showed that activation of D2 downregulated cAMP and PKA activity, inhibiting CREB phosphorylation and subsequent transcription (Hanami et al., 2013; Wang, Han et al., 2021). These data clearly show that D2-like receptors can have direct effects on osteoclastogenesis and bone mineralization. However, the activity of L-DOPA in vitro (where it would need to be converted to dopamine to activate dopamine receptors), as well as the L-DOPA–mediated bone loss and lack of effectiveness of D2 agonists on in vivo bone loss (Handa et al., 2019) indicate that the role of dopamine is more complex than just D2-signaling driving osteoclastogenesis and is likely affected by local changes in dopamine availability.

While D2-like dopamine receptor signaling seems to primarily regulate osteoclastogenesis and subsequent bone resorption, signaling via D1-like receptors was found to influence osteoblast activity. Treatment with dopamine (5 × 10^−5^M) increased the proliferation of the immature murine osteoblast cell line MC3T3-E1 (Lee, Tsang et al., 2015), although in another study using this cell line, dopamine suppressed osteoblast mineralization and osteoblast marker expression that was not seen in primary murine osteoblasts (Motyl et al., 2017). Treatment with SKF38393 (D1-like receptor agonist) increased osteoblast differentiation and expression of osteogenic genes in both bone marrow–derived stem cells and MC3T3-E1 (Zhu et al., 2022). In both studies these effects were blocked by treatment with the D1-like antagonist SCH23390, either alone or in combination with the D3 receptor antagonist GR103691. The osteogenic effect of D1 activation was also shown by increased osteogenesis in vivo in dexamethasone-treated mice, where treatment with SKF38393 reduced bone loss. The effects of dopamine on osteoblasts were mediated via ERK1/2 signaling, as inhibiting ERK1/2 signaling blocked SKF38393-induced osteogenesis in MC3T3-E1 cells (Zhu et al., 2022). In contrast, in osteoclasts, blocking ERK1/2 had no impact on osteoclastogenesis mediated by D2-like receptors (Wang, Han et al., 2021). Osteoblasts also show sensitivity to higher levels of dopamine, as concentrations above 5 × 10^−6^M inhibited the osteogenic activity of bone mesenchymal stem cells (Wang et al., 2020) and concentrations of 10^−4^M dopamine were cytotoxic (Lee, Tsang et al., 2015). These data indicate important roles for dopamine in bone homeostasis, with D2-like receptor activation reducing bone loss by diminishing osteoclast activity and D1-like receptor activation promoting osteogenesis by promoting osteoblast differentiation and activity.

In addition to these homeostatic interactions, many neurologic disorders associated with changes in dopamine concentration or metabolism, including PD (Bezza et al., 2008; Dobson et al., 2013; Torsney et al., 2014), depression (Bab and Yirmiya, 2010; Cizza et al., 2010), and schizophrenia (Renn et al., 2009; Lin et al., 2015), are also associated with lower bone mineral density (osteoporosis). In the MPTP model of PD, mice with dopaminergic degeneration had lower bone mass, lower mineralized area of bone surface, and lower bone formation rate. This was due to changes in regulation of osteoblasts and osteoclasts mediated by dopamine neurons via dysregulation of serum prolactin levels (Handa et al., 2019). In another study, exposure to serum from MPTP-treated rodents with significantly reduced striatal dopamine was cytotoxic to osteoblasts, negatively affecting their proliferation, differentiation, and mineralization, while also enhancing osteoclast differentiation (Ali et al., 2019). These data indicate that the pathologic disruption of dopamine regulation, possibly through alterations in prolactin release, could dysregulate osteoblast and osteoclast function and influence bone resorption and mineralization. As noted in the prior sections, domperidone, a selective D2/D3 antagonist, also increases prolactin and alters phagocytic and oxidative burst activity in rodent macrophages (Carvalho-Freitas et al., 2008, 2011). This suggests changes in prolactin mediated by dopamine signaling could also influence bone health through indirect effects on immune function.

Dopamine-mediated increases in osteoporosis are also associated with the treatment of neurologic diseases, and several groups have examined the impact of dopaminergic therapeutics on bone health and found sex differences (Di Somma et al., 1998; Motyl et al., 2017; Uddin et al., 2018). Several of these effects are also associated with prolactin, as many typical and atypical antipsychotics induce dose-dependent hyperprolactinemia and hypogonadism, resulting in bone loss (Brown and Mezuk, 2012). This is mediated via changes in dopamine signaling, as many antipsychotics antagonize D2-like receptors, and antagonism of D2 receptors on lactotroph cells in the pituitary releases prolactin into the serum (Gudelsky, 1981). Reducing the use of D2 antagonists can prevent these effects, as use of clozapine, a nonprolactin-raising antipsychotic that does not have high affinity for D2 dopamine receptors, increased bone mineral density in schizophrenic patients (Lin et al., 2019) and reduced development of osteoporosis when used alongside prolactin-raising antipsychotics in schizophrenics (Qiu et al., 2020). However, the use of D2 receptor agonists to treat men with prolactinemia did not restore bone mineral density, although it did stop additional losses (Di Somma et al., 1998). This suggests changes in dopamine signaling that alter prolactin levels may only be part of the mechanism by which dopamine regulates bone health.

Indeed, while MPTP-induced dopaminergic degeneration resulting in bone loss was associated with elevated prolactin, ovariectomy-induced bone loss was significantly ameliorated by MPTP treatment (Handa et al., 2019). Similarly, treatment with the D2-like receptor antagonist risperidone increased both prolactin levels and bone loss in rats, but in ovariectomized rats, where prolactin levels are not changed, risperidone still caused bone loss (Motyl et al., 2017). Treating rats with methylphenidate, which blocks dopamine reuptake and increases extracellular dopamine levels, led to a methylphenidate- and sex-dependent decrease in bone integrity due to increased osteoclast activity (Uddin et al., 2018). Studies show that that polymorphisms in D2 and D4 are associated with reduced bone mineral density (Yamada et al., 2003; Chiang et al., 2020). These data indicate that hyperprolactinemia-induced hypogonadism is not the sole mechanism by which dysregulation of dopaminergic signaling could regulate bone health and that other mechanisms may play a significant role in the pathogenesis of dopamine-mediated bone loss. Further, considering the divergent functions of dopamine in regulation of both osteogenesis and osteoclastogenesis, receptor-specific activation of the dopaminergic system could be a useful therapeutic approach for stimulating bone mineralization and/or reducing bone resorption in bone pathology. Additional investigation of other immune cell populations within bone such as osteal macrophages, which are distinct from osteoclasts (Sinder et al., 2015) and are increasingly considered important to bone remodeling, and lymphocytes may elucidate a deeper contribution of dopamine in bone homeostasis.

##### a. Rheumatoid Arthritis

RA is an autoimmune disorder of the bones and joints characterized by irreversible destruction of the joints that results in progressive disability. Studies indicate that the progression of RA is significantly impacted by the dopaminergic system (Pacheco et al., 2014; Capellino, 2020). Synovial fibroblasts, the resident cells of the intimal lining layer of synovial tissue, express all five subtypes of dopamine receptors, as well as DAT and TH (Capellino et al., 2010, 2014). There are some differences in dopamine receptor expression across synovial layers, but all subtypes were expressed in the invasion zone, with higher levels of D1, D2, and D5 staining seen in this region than in other layers of synovial tissue in RA (van Nie et al., 2020). Patients with RA showed higher levels of D1-like receptors on synovial fibroblasts relative to patients with osteoarthritis, and agonist-mediated activation of both D1-like (fenoldopam) and D2-like (ropinirole) receptors increases the migration of these cells in patients under the age of 75. In older RA patients, dopamine receptor expression, but not responsiveness, was reduced, and in patients of all ages dopamine moderately decreased the release of IL-6 and IL-8 (Capellino et al., 2014; van Nie et al., 2020). The number of synovial fibroblasts expressing D3 is positively correlated with increased levels of the antioxidants superoxide dismutase and catalase in RA patients, while there is a negative correlation between the numbers of D3-expressing mast cells in synovial fluid and disease severity (Xue, Li et al., 2018). The expression of D2 in B-cells is also negatively correlated with disease activity in RA patients (Wei et al., 2016). These findings suggest that dopamine signaling is largely anti-inflammatory in RA, promoting resolution of joint inflammation.

However, other studies have found that dopamine signaling can exacerbate RA. In a murine model of RA, cartilage destruction was exacerbated via an IL-6-dependent mechanism initiated by dopamine release from DCs, which induced the differentiation of T_h_17 cells and the release of inflammatory cytokines. This effect was blocked by the D1-like receptor antagonist SCH23390 (Nakano et al., 2011). Antagonizing D1-like receptors with SCH23390 also suppressed the severity of collagen-induced arthritis in DBA/1 mice. Blockade of D1-signaling also inhibited RANKL induced in vitro osteoclastogenesis in BMDM exposed to low levels of dopamine (10^−7^M), an effect that was blocked by activating D1-like receptors with A68,930 (Nakashioya et al., 2011). Antagonizing D2-like receptors with haloperidol restored biomarkers of RA, including serum rheumatoid factor, matrix metalloprotinease-3, serum IgG, and antinuclear antibody to normal levels in adult female albino rats (Fahmy Wahba et al., 2015), suggesting activation of D2-like receptors dysregulates these factors. These data show that dopamine can affect the development of RA and suggest that dopamine has anti-inflammatory effects on synovial cells. However, the systemic effects of dopamine in vivo seem to potentially promote RA despite the activity in individual cell types, and it is likely that the interaction between resident and infiltrating cells may drive the overall impact of dopamine on this disease.

#### 5. Kidney

Dopamine is critical to healthy kidney function, regulating fluid homeostasis and electrolyte balance within the body (Jose et al., 1998; Armando et al., 2011; Cuevas et al., 2013). Several groups have identified dopamine receptors in all regions of the nephron, the functional unit of the kidney. While all five subtypes of dopamine receptors have been identified in the nephron, the distribution of the five subtypes varies in each region (Katayama et al., 1989; Gao et al., 1994; Grupp et al., 1998; Ricci et al., 2002; Shin et al., 2003; Nurnberger et al., 2004; Armando et al., 2011; Han et al., 2015), suggesting that individual areas of the nephron respond differently to dopamine stimulation.

Much of the literature examining the function of renal dopamine focuses on the renal proximal tubules. These epithelial cells enable the secretion of waste products and resorption of nutrients from glomerular filtrate (Aperia, 2000; Wang and Kestenbaum, 2018). Renal proximal tubules are also the major source of dopamine in the kidney, extracting L-DOPA in the filtrate from the glomerulus and converting it to dopamine using the enzyme AADC (Fig. 2), which is highly expressed in this region of the nephron (Goldberg, 1977; Wang et al., 1997; Daubner et al., 2011). Notably, renal proximal tubule cells do not express dopamine beta hydroxylase or TH and cannot synthesize dopamine from tyrosine nor convert dopamine to norepinephrine (Lewis et al., 1990; Catelas et al., 2020). Locally produced dopamine can act as an autocrine regulator of these processes through activation of D1-like receptors, which regulates transporter uptake and release of ions and other nutrients (Carey, 2001; Pedrosa et al., 2004). These cells also possess some intrinsic immune characteristics that allow them to function as immune responders in the case of kidney insult (Nakhoul and Batuman, 2011).

Nephritis, or kidney inflammation, is often associated with autoimmune illnesses but can arise secondarily as an adverse reaction to medication or in response to other pre-existing conditions such as diabetes or obesity. Dopamine is linked to renal inflammation and acute renal injury through D2, and downregulation of this receptor increases susceptibility to renal inflammation, which was independent of dopamine-mediated blood pressure regulation in a mouse model (Jiang et al., 2014; Konkalmatt et al., 2016). SNPs in D2 are associated with this increase in renal inflammation, and human renal proximal tubule cells with D2 SNPs showed decreased D2 expression and function (Jiang et al., 2014). In humans, the TaqI polymorphism in Drd2 genes is associated with kidney pathologies such as hypertension, as well as obesity (Thomas et al., 2000; Fang et al., 2005). There was an increase in the inflammatory cytokines TNF-*α* and IL-6 and the profibrotic factor TGF-*β*1 when D2 was silenced in proximal renal tubule cells (Jiang et al., 2014; Zhang, Jiang et al., 2016), suggesting that D2 signaling is anti-inflammatory in renal proximal tubules and protects against renal inflammation.

Activation of D2-like receptors on these cells may also affect inflammation, downregulating production of ROS via regulation of paraoxonase 2 and Sestrin2 (Yang et al., 2012; Yang, Cuevas et al., 2014). D2 may modulate renal inflammation through the Akt pathway. Downregulation of D2 in the kidney increased Akt phosphorylation and cyclin D1 expression, which are downstream targets of Akt but did not affect phosphatidylinositol 3-kinase, which is upstream of Akt (Zhang, Jiang et al., 2016). These data suggest dopamine acts through D2 to decrease Akt signaling, preventing the upregulation of downstream inflammatory signals to protect the kidney against inflammation-induced injury. This is supported by mouse studies in which D2 was silenced by small interfering RNA, and renal-specific rescue of D2 reduced the expression of inflammatory factors, normalized blood pressure, and preserved renal function (Konkalmatt et al., 2016). However, selective antagonism of D2-like receptors, specifically D3, can reverse diabetes-induced glomerular hyperfiltration (Luippold et al., 2000), and D3 antagonism also mediated beneficial changes in renal morphology and albuminuria in a model of type 2 diabetes (Gross et al., 2006). Further, the D4 agonist, PD168077, inhibits Na^+^/K^+^-ATPase activity in rat renal proximal tubule cells (Tang et al., 2017). These data suggest that D2-like receptors regulate renal inflammation and subsequent changes in kidney function but that different types of D2-like receptors have different effects on renal homeostasis.

There has been less investigation into the role of D1-like receptors in renal inflammation and immune function. In renal proximal tubule cells, D1 signaling is linked to the inhibition of sodium transporters, Na^+^–K^+^–ATPase and Na^+^/H^+^ exchangers (Aperia, 2000), and abnormalities in D1-like receptors contribute to increased blood pressure and hypertension, as discussed in the following text. This may be mediated by effects on infiltrating immune cells rather than directly on the kidney, as treating mice with the D1 agonist A68930 ameliorated renal dysfunction and markedly reduced macrophage and T-cell infiltration in a mouse model of acute ischemic kidney injury (Cao et al., 2020). These effects were mediated by decreased renal and serum inflammatory cytokine production (TNF-*α*, IL-6, and IL-1*β*), which was partially due to inhibition of the NLRP3 inflammasome (Cao et al., 2020). These anti-inflammatory effects on cytokine production and NLRP3 correlate with similar results in other models using rodent macrophages but differ from those found in human macrophages. Treatment of rodent renal proximal tubules also reduces production of ROS (Li et al., 2009). Further, rats treated with LPS and older rats with increased oxidative stress had reduced D1 function in renal proximal tubule cells (Asghar et al., 2008, 2009), supporting an interaction between D1 activity and inflammation / immune activity. Together, these findings suggest that dopamine receptor signaling is required for maintaining homeostasis and that both types of dopamine receptors can be anti-inflammatory in renal proximal tubule cells, but that the signaling mechanisms mediating the effects of each type of dopamine receptor are likely distinct.

##### a. Hypertension

Intrarenal dopamine can increase with dietary sodium intake (Bertorello et al., 1988; Harris and Zhang, 2012), although exogenous dopamine can produce a biphasic blood pressure pattern associated with renal regulation in humans. In a human dose-response study, higher doses of dopamine (7.5, 10, and 12.5 *μ*g/kg/min) increased cardiac output and mean arterial pressure, while lower doses (1–2 *μ*g/kg/min) decreased mean arterial pressure (Olsen et al., 1997). As mentioned in prior sections, at high doses, dopamine can interact with adrenergic receptors, including the *α*-adrenergic receptors that mediate the renal vasodilatory effects (Olsen et al., 1997). Dopamine also influences hypertension directly through dopamine receptors, and activation of D1-like receptors can induce renal vasodilation, diuretic, and natriuretic effects (Yatsu et al., 1997; Banday and Lokhandwala, 2008). Signaling through D1-like receptors decreases Na^+^ entry by inhibiting the Na^+^/H^+^ exchanger 3 and the Na^+^/K^+^–ATPase(Lee, 1989; Aperia, 1994; Banday and Lokhandwala, 2008; Wang et al., 2017; Banday et al., 2019).

Defects in D1-like receptors and renal dopaminergic signaling are highly correlated with the development of hypertension (Jose et al., 1996; Zeng et al., 2005; Banday and Lokhandwala, 2008; Yang, Villar et al., 2021). In spontaneously hypertensive rats, carbidopa, which decreases peripheral dopamine by inhibiting peripheral dopamine synthesis, significantly accelerated the development of hypertension, decreased urinary sodium excretion, and decreased urinary and renal dopamine levels (Yoshimura et al., 1987). In humans, blockade of D1 signaling by treatment with the long-lasting D1 antagonist ecopipam induced significant increases in blood pressure (Haney et al., 2001), while treatment with the D1 agonist fenoldopam lowered blood pressure (Tumlin et al., 2000; Hammer et al., 2008), potentially by acting on renal D1 receptors to mediate renal vasodilation and natriuresis (Armando et al., 2011). Similarly, silencing D1 in mice in the kidney increased blood pressure, which was normalized after renal tubule-specific rescue with wild-type D1 (Tiu et al., 2020). These outcomes may be mediated by the interaction of D1 receptors with lipid rafts, which is necessary for D1 signaling in the kidney (Yu et al., 2004; Martinez et al., 2020; Tiu et al., 2020). These data indicate that dopamine signaling through D1-like receptors can regulate secondary hypertension due to changes in the kidney mediated by the interaction between dopamine and the Na^+^ channels in the nephron.

As noted in the previous section, D2-like receptor signaling is associated with the regulation of inflammation and ROS in renal tubule cells. Recent reviews have highlighted a central role for oxidative stress in dopaminergic changes associated with the development of hypertension (Cuevas et al., 2013; Banday and Lokhandwala, 2015; Olivares-Hernandez et al., 2021; Qaddumi and Jose, 2021; Yang, Villar et al., 2021). This suggests that activation or antagonism of these receptors could influence hypertension by regulating the inflammatory state in the proximal tubule. There are also studies suggesting that D2-like receptor-mediated changes in kidney function can directly influence hypertension, but these have mostly focused on D3. While there is little to no change in D2-like receptor expression in hypertensive rats compared with normotensive rats (Luippold et al., 2003; Shin et al., 2003), mice with homozygous or heterozygous knockout of Drd3 showed elevated blood pressure as a result of elevated renal renin production and sodium retention (Asico et al., 1998; Staudacher et al., 2007). Expression of D3 was increased in the PBMC of hypertensive individuals (Ricci et al., 1997) and treating rats with the D3 agonist 7-OH-DPAT enhanced kidney function, which was blocked by pretreatment with a D3 specific antagonist (U-99194A) but not with the D2-like antagonist sulpiride (Luippold et al., 1998). Further studies are needed to dissect out the specific signaling pathways and mechanisms (inflammation vs. changes in renin production) that mediate the effects of D2-like receptors in the kidney. Overall, these data show that dopamine clearly influences the development of hypertension and likely other kidney diseases and suggest that continued investigation of dopaminergic agents is a promising direction for the treatment of hypertension.

#### 6. Cardiovascular System

Dopamine plays a central role in blood pressure regulation and cardiovascular function. Within the cardiovascular system, circulating dopamine originates from both CNS-dependent sources and peripheral neurons (Lokhandwala and Barrett, 1982). Interestingly, a large proportion of dopamine in the cardiovascular circulation is conjugated as dopamine sulfate (Yoneda et al., 1983). As discussed previously, dopamine sulfate is biologically inactive, and the role of dopamine sulfate in cardiovascular function remains unclear. It is possible that fluctuations in active and inactive dopamine in the circulation influence the state of various immune cells in the circulation and/or contribute to cardiovascular homeostasis, which may uncover mechanisms and aid in therapeutic discovery for pathogenic states. Within the cardiovascular system, dopamine can act through both D1- and D2-like receptors expressed in the human heart and in renal, coronary, mesenteric, and cerebral arteries in various animal models, although expression levels vary between cell types and species (Berkowitz, 1983; Missale et al., 1988; Berkowitz and Ohlstein, 1984; Ricci et al., 1994; Amenta et al., 1990; Gómez de Jesus, 2002; Cavallotti et al., 2010; Zingales et al., 2021). Substantial data across several decades shows that dopaminergic agents affect vasodilation and vasoconstriction in vitro and in humans, dogs, and mice (Brodde, 1982; Lokhandwala and Barrett, 1982; Zhao et al., 1990). Moreover, D1 receptors were localized to the postjunctional regions due to their sensitivity to sympathectomy, while D2 receptors were localized to the adventitia, adventitial–medial border, and intimal layer (Amenta et al., 1990). Interestingly, studies have shown that there is no D1 or D2 receptor expression in the veins (Okamura and Toda, 1995; Kim et al., 1999), indicating that the effects of dopamine on the vasculature are primarily centered around arterial vessels.

These and other data suggest that dopamine receptors are differently expressed within the layers of the blood vessels; thus, the effects of dopamine depend on the subsets of receptors and concentration of dopamine present in each region. In proximal arteries, dopamine induces dose-dependent contraction, while in distal arteries, low concentrations of dopamine led to dilation and high concentrations led to contraction (Toda et al., 1989). Intravenous dopamine increased the mean arterial pressure to a greater extent than the *β*-adrenergic receptor agonist dobutamine in patients undergoing a surgical procedure, although dobutamine generated more consistent responses among all patients (Suominen et al., 2004). This may result from dopamine activation of both dopamine and *α*- and *β*-adrenergic receptors, unlike dobutamine. Thus, while dopamine can improve cardiac function, it has a larger side effect profile than dobutamine (Loeb et al., 1977; Maekawa et al., 1983; Bistola et al., 2019). This is corroborated by catecholaminergic (dopamine and norepinephrine) effects on adrenergic receptors in the vessels that mediate cardiovascular effects such as vascular remodeling (Toda et al., 1989; Erami et al., 2005; Faber et al., 2007). Therefore, dopamine signaling through both dopamine and adrenergic receptors influences the maintenance of vascular smooth muscle and changes in vascular tone.

Dopamine can also influence cardiac function, as patients treated with ibopamine, an agonist at D1-like receptors and *α*-adrenergic receptors, increased mortality in patients with heart failure (Hampton et al., 1997). Dopamine and drugs acting on dopamine receptors have long been known to have the potential to be proarrhythmic (Tisdale et al., 1995a, 1995b, 2020), although the incidence of dopamine-associated arrythmia is relatively low. In addition to being caused by therapeutics, arrythmias associated with dopamine or other catecholamines may result from Takatsubo syndrome, an acute dysfunction of the left ventricle associated with high mortality and extremely high levels of catecholamines following intravenous administration of dopamine (Nakagawa et al., 2016). The mechanism underlying the dopamine arrythmia may be dopamine signaling in cardiomyocytes, as activation of D1 in mouse cardiomyocytes is associated with ventricular arrythmia, which is mediated by calcium disruptions (Yamaguchi et al., 2020). As previously noted, dopamine mediates calcium signaling through several pathways, including G*_α_*_q_ signaling, and dopamine also increases L-type calcium channel currents in rabbit cardiomyocytes through D1 and *β*-adrenergic receptors (Ding et al., 2008). Other studies have also shown that activation of *β*-adrenergic receptors can enhance L-type calcium channel activity and cardiac myocyte cell death during heart failure (Zhang et al., 2001; Collis et al., 2007; Wang et al., 2010). As dopamine can act on adrenergic receptors at higher concentrations, changes in dopamine levels around the heart could promote these effects. In conditions with increased sympathetic nervous system activity, there is an associated increase in plasma dopamine concentrations (Matt and Gaskill, 2020), which is an underlying mechanism of Takatsubo syndrome (Bucolo et al., 2019). Therefore, within the heart, dopamine signaling via both D1 and *β*-adrenergic receptors could affect calcium flux and interfere with cardiac rhythm, creating negative effects on long term mortality.

Arrhythmias, acute heart failure, and myocardial infarction correlate with increasing age (Aronow, 2006; Ganjehei et al., 2014; Fallon, 2019), and a study of D2 in the heart and coronary vessels of rats found significant age-related decreases in these regions (Cavallotti et al., 2002). Changes in D1-like receptor expression were not seen, but dopamine receptor expression was altered with age in neurons (Hemby et al., 2003), PBLs (Barili et al., 1996), and kidneys (Chugh et al., 2013), so it is reasonable to posit that expression of other types of dopamine receptors is also reduced. A Drd3 knockout mouse was shown to mimic age related changes in cardiac dysfunction and remodeling (Johnson et al., 2013) and loss of D3 signaling in mouse cardiac fibroblasts from either antagonism or knockout significantly reduced fibroblast migration and proliferation in vitro (Kisling et al., 2021). These data indicate that both types of dopamine receptors can regulate cardiac function and suggest that changes in the expression ratio of dopamine receptor subtypes in the heart may create an imbalance in the response to endogenous and exogenous dopamine that can exacerbate underlying heart conditions. Overall, the role of dopamine in the cardiovascular system is mediated by both dopamine receptors and adrenergic receptors, particularly during treatment with drugs that induce higher concentrations of dopamine in this compartment. Dissecting the receptors and signaling pathways mediating the specific positive and negative effects of dopamine on the cardiovascular system could support the development of more specific or biased catecholamine receptor agonists with a lower side effect profile.

##### a. Sepsis

Sepsis is a life-threatening condition resulting from physiologic and biochemical changes due to infection. Sepsis leads to abnormal distribution of blood flow to tissues; symptoms include arterial hypotension, altered mental status, fever, tachycardia, and difficulty breathing, and more progressive disease results in multiorgan failure, which increases mortality risk (Peach, 2017; Zhou, Zhu et al., 2021). In addition to antibiotics and fluids, vasoactive catecholamines such as dopamine are used clinically to manage shock and refractory heart failure (Reid and Thompson, 1975; Goldberg et al., 1963; Doggrell, 2002). Intravenous administration of dopamine (the only approved route of administration) or the D1-like receptor agonist fenoldopam are positive inotropes that lead to rapid recovery in these acute conditions (Doggrell, 2002; Feketeova et al., 2018; Zingales et al., 2021). Activation of both dopamine and adrenergic receptors is thought to be involved in the positive effects during sepsis (Ruffolo et al., 1984; Carey and Jacob, 1989), although some studies find use of dopamine over a more specific adrenergic agonist may create a higher risk of side effects (Pollard et al., 2015). High doses of dopamine can increase the risk of adverse effects in the treatment of sepsis (Jentzer and Hollenberg, 2021), and a trial comparing the hemodynamic efficacy of dopamine to norepinephrine and dobutamine in elderly individuals with sepsis found that norepinephrine showed superior effects in improving hemodynamic stability and vascular elasticity and reducing inflammatory markers in the circulation (Zhou, Cui et al., 2021). Further, two different meta-analysis of observational and randomized trials of septic patients (controlled for heterogeneity) treated with dopamine or norepinephrine (varying doses) reported that dopamine administration was associated with a higher incidence of arrhythmic events and greater mortality than norepinephrine treatment (De Backer et al., 2012; Avni et al., 2015).

However, these studies did not consider differences in dopamine dose or any potential age-related effects, so it is possible that these effects are confounders. There may also be age-related differences in how immune cells and the cardiovascular system respond to dopamine, which need to be further explored. In addition, a number of studies show that dopamine treatment is as safe and effective as other treatments, and randomized trials in neonates and pediatric patients who were septic and treated with dopamine or epinephrine showed no difference in long-term mortality and comparable efficacy and safety (Baske et al., 2018; Kohn-Loncarica et al., 2020). In patients treated with norepinephrine, dopamine, or a combination of norepinephrine and vasopressin, the recommended vasoactive therapy for sepsis, dopamine shortened intensive care unit and hospital stays compared with the other therapies (Zhou, Zhu et al., 2021). Dopamine may induce these effects via regulation of the immune response in circulation (Beck et al., 2004), as dopamine can suppress inflammatory cytokines (IL-6, IL-8, and TNF-*α*) and increase production of IL-10, although, as previously noted, these effects are complex and vary with cell type, dopamine concentration, dopamine receptor ratio, and species. Increased dopamine output from the adrenal glands attenuated the increase in serum TNF-*α* levels in a mouse model of endotoxemia (Shimojo et al., 2019), and in diabetic, septic mice, treatment with the D1-like agonist fenoldopam attenuated inflammation by inhibiting p65 NF-kB phosphorylation in splenocytes (Feketeova et al., 2018). In another study, dopamine and fenoldopam, but not the D2-like agonist pergolide, attenuated systemic inflammation. These effects may be due to vagus nerve stimulation of adrenal catecholamine production (Torres-Rosas et al., 2014), as patients with sepsis often show adrenal insufficiency (Marik and Zaloga, 2003). A separate study found that very high levels of dopamine (1.5 × 10^−4^M) prevent *Staphylococcus aureus*–induced sepsis in mice through activation of D5 on macrophages, inhibiting TLR2-induced inflammation and NF-*κ*B activation (Wu et al., 2020).

These data suggest that dopamine may be as safe as other vasopressors in some populations and that the beneficial effects may be mediated by activation of D1-like receptors, which are important in the regulation of inflammation during sepsis. This correlates well with many studies discussed earlier, showing that activation of D1-like receptors in rodents and in cells treated with LPS was anti-inflammatory and supports the concept that dopamine has distinct effects in inflammatory and noninflammatory environments. Defining the mechanisms by which specific dopamine receptors affect inflammation in sepsis will be useful in the management of this condition, enabling development or repurposing of targeted agonists that leverage anti-inflammatory activity. However, until these mechanisms and the way they affect distinct populations are better understood, norepinephrine will remain the first-line vasopressor because of its more consistent effects and better safety profile across all populations.

## V. Concluding Remarks

The data discussed in this review clearly show that dopaminergic immunomodulation has substantial impacts across both the CNS and periphery, broadly affecting homeostatic function and disease pathogenesis. Although spread across a wide array of cell types, systems, and diseases, and despite very limited data in some contexts, there are clear immunoregulatory roles of dopamine in different organs and diseases, highlighting the potential for dopaminergic immunomodulation in the treatment of a wide array of pathogenic conditions. Many of the studies discussed highlight the interconnected nature of the impact of dopamine, showing, for example, that alterations in dopamine levels during neurologic diseases that can influence CNS immune functions can also drive immunologic changes in bone or gut health. Thus, dopamine, and the therapeutics that modulate dopamine signaling, facilitate bidirectional communication between the immune system and many other organ systems and cell types. This means that consideration of the immunologic effects of dopamine is a critical aspect in the studies of many different systems and diseases.

However, information about dopaminergic immunomodulation, intersystem crosstalk via dopamine and dopamine signaling, and peripheral dopamine in general can be difficult to access, making it challenging to plan and analyze these types of studies. The primary purpose of this review is to ameliorate those issues by examining and synthesizing ideas regarding dopaminergic immunomodulation across these research areas, as well as discussing the problems and considerations that are important to the execution and evaluation of this type of research. This will hopefully facilitate the identification of common underlying pathways upon which dopamine and immunomodulation converge in different contexts. For example, data that show changes in cytokines that are similar across psychiatric disorders and are similarly reduced by the same medication. More careful analyses of these overlaps could lead to considerations in the effects of prescribed dopaminergic and immune therapeutics on other diseases, as well as comorbidities within a particular individual. Indeed, the synergistic effect of immune activation and increased dopamine levels due to therapeutics could have important implications in the treatment of immune-compromised individuals and populations with dysfunctional dopamine signaling.

Overall, these data demonstrate the importance of further expanding our understanding of dopaminergic immunoregulation in the CNS and, especially, in the periphery. This is critically important for the treatment of diseases with both CNS and peripheral symptoms that may have a common, dopaminergic basis, such as GI symptomology in PD or depression in PLWH. Research that further defines and validates the crosstalk between the immune system and CNS in the development of disease is also needed. Development of this research will require collaborative efforts using a broad array of research modalities: from human trials, imaging studies, and in vivo animal systems to multiple preclinical models, including human in vitro models such as induced pluripotent stem cell mono- and coculture systems and organoids, ex vivo primary cells/tissues, and postmortem human samples, as we recently suggested in the context of neuroHIV and neuropsychiatric diseases (Nickoloff-Bybel et al., 2020; Matt, 2021). The integration of these strategies with pharmacogenetic testing, predictive modeling, and the mining of other high-dimensional proteomic and metabolomic analyses can be leveraged to help identify new candidate biomarkers, as well as develop and repurpose dopaminergic therapeutics. Ultimately, this will translate into a better understanding of the symptoms and etiology of dopaminergic diseases, enabling the development and use of more efficacious diagnostic, preventive, and therapeutic interventions.

## Abbreviations

AADC: aromatic L-amino acid decarboxylase
ADHD: attention-deficit/hyperactivity disorder
AIDS: acquired immune deficiency syndrome
Akt: protein kinase B
ART: antiretroviral therapy
BH4: tetrahydrobiopterin
BMDM: bone marrow–derived macrophages
CCL2: C-C motif chemokine ligand 2
CCR5: C-C chemokine receptor type 5
CCR9: C-C motif chemokine receptor 9
CD: Crohn’s disease
CNS: central nervous system
COMT: catechol-O-methyltransferase
CREB: cAMP response element-binding protein
CRP: C-reactive protein
CSF: cerebrospinal fluid
CXCL8: C-X-C motif chemokine ligand 8
CXCR3: CXC chemokine receptor 3
CYP2D6: cytochrome P450 2D6
DAMP: damage-associated molecular pattern
DAT: dopamine transporter
DOPAC: 3,4-dihydroxyphenylacetic acid
EAE: experimental autoimmune encephalomyelitis
ERK1/2: extracellular signal-related protein kinase 1/2
Fc*γ*: Fc-gamma receptor
GAD: generalized anxiety disorder
GI: gastrointestinal
GPCR: G-protein coupled receptors
G_s_: G_s_ heterotrimeric G protein
G*_α_*_i_: G_i_ alpha subunit of the G_i_ heterotrimeric G protein
G*_α_*_s_: G_s_ alpha subunit of the G_s_ heterotrimeric G protein
H_2_O_2_: hydrogen peroxide
HIV: human immunodeficiency virus
hMDM: human monocyte-derived macrophages
HPA: hypothalamic–pituitary–adrenal
HVA: homovanillic acid
IBD: inflammatory bowel disease
IL: interleukin
IP3: inositol triphosphate
I*κ*B: IkappaB
JNK: c-Jun N-terminal kinase
L-DOPA: levodopa
LID: levodopa-induced dyskinesia
LPS: lipopolysaccharide
MAO: monoamine oxidase
MAO-B: monoamine oxidase B
MAPK: mitogen-activated protein kinase
M-CSF: macrophage colony-stimulating factor
MHC: major histocompatibility complex
MIA: maternal immune activation
mPFC: medial prefrontal cortex
MPTP: 1-methyl-4phenyl-1, 2, 3, 6-tetrahydropyridine
MS: multiple sclerosis
NAc: nucleus accumbens
neuroHIV: neurologic human immunodeficiency virus
NF-*κ*B: nuclear factor kappa-light-chain-enhancer of activated B-cells
NK cell: natural killer cell
NLR: nucleotide-binding oligomerization domain-like receptor
NLRP3: nucleotide-binding oligomerization–like receptor family pyrin domain containing 3
NO: nitric oxide
PAMP: pathogen-associated molecular pattern
PBL: peripheral blood lymphocyte
PBMC: peripheral blood mononuclear cell
PD: Parkinson’s disease
PET: positron emission tomography
PFC: prefrontal cortex
PKA: protein kinase A
PKC: protein kinase C
PLC*β*: protein lipase C-*β*
PLWH: people living with human immunodeficiency virus
PP2A: protein phosphatase 2
PRR: pattern recognition receptor
PTSD: post-traumatic stress disorder
RA: rheumatoid arthritis
RANKL: receptor activator of nuclear factor kappa-Β ligand
ROS: reactive oxygen species
SAD: social anxiety disorder
SbN: substantia nigra
SIV: simian immunodeficiency virus
SNP: single nucleotide polymorphism
SNRI: serotonin and norepinephrine reuptake inhibitor
SSRI: selective serotonin reuptake inhibitors
SUD: substance use disorders
T_f_h: T follicular helper cell
TGF: transforming growth factor
T_h_: T-helper
TH: tyrosine hydroxylase
TLR: toll like receptor
TLR: toll-like receptor
TNF-*α*: tumor necrosis factor alpha
T_regs_: regulatory T-cells
T_rm_: CD8^+^ T resident memory cells
TSPO: translocator protein
TYRP: tyrosinase-related proteins
VMAT2: vesicular monoamine transporter 2
VTA: ventral tegmental area
